# Supplementary material for: The secreted protein Amuc_1409 from Akkermansia muciniphila improves gut health through intestinal stem cell regulation
Source: Nat Commun. 2024 Apr 6;15:2983. doi: 10.1038/s41467-024-47275-8 (PMC10998920; doi:10.1038/s41467-024-47275-8)
Supplement: Supplementary file 1 — Supplementary Information [file 41467_2024_47275_MOESM1_ESM.pdf]

Supplementary information

The secreted protein Amuc_1409 from *Akkermansia muciniphila* improves gut health through intestinal stem cell regulation

Eun-Jung Kang, Jae-Hoon Kim, Young Eun Kim, Hana Lee, Kwang Bo Jung, Dong-Ho Chang, Youngjin Lee, Shinhye Park, Eun-Young Lee, Eun-Ji Lee, Ho Bum Kang, Moon-Young Rhyoo, Seungwoo Seo, Sohee Park, Yubin Huh, Jun Go, Jung Hyeon Choi, Young-Keun Choi, In-Bok Lee, Dong-Hee Choi, Yun Jeong Seo, Jung-Ran Noh, Kyoung-Shim Kim, Jung Hwan Hwang, Ji-Seon Jeong, Ha-Jeong Kwon, Hee Min Yoo, Mi-Young Son, Yeon-Gu Kim, Dae-Hee Lee, Tae-Young Kim, Hyo-Jung Kwon, Myung Hee Kim, Byoung-Chan Kim, Yong-Hoon Kim, Dukjin Kang, and Chul-Ho Lee

Supplementary Figures 1–16 & Legends

Supplementary Tables 1–9

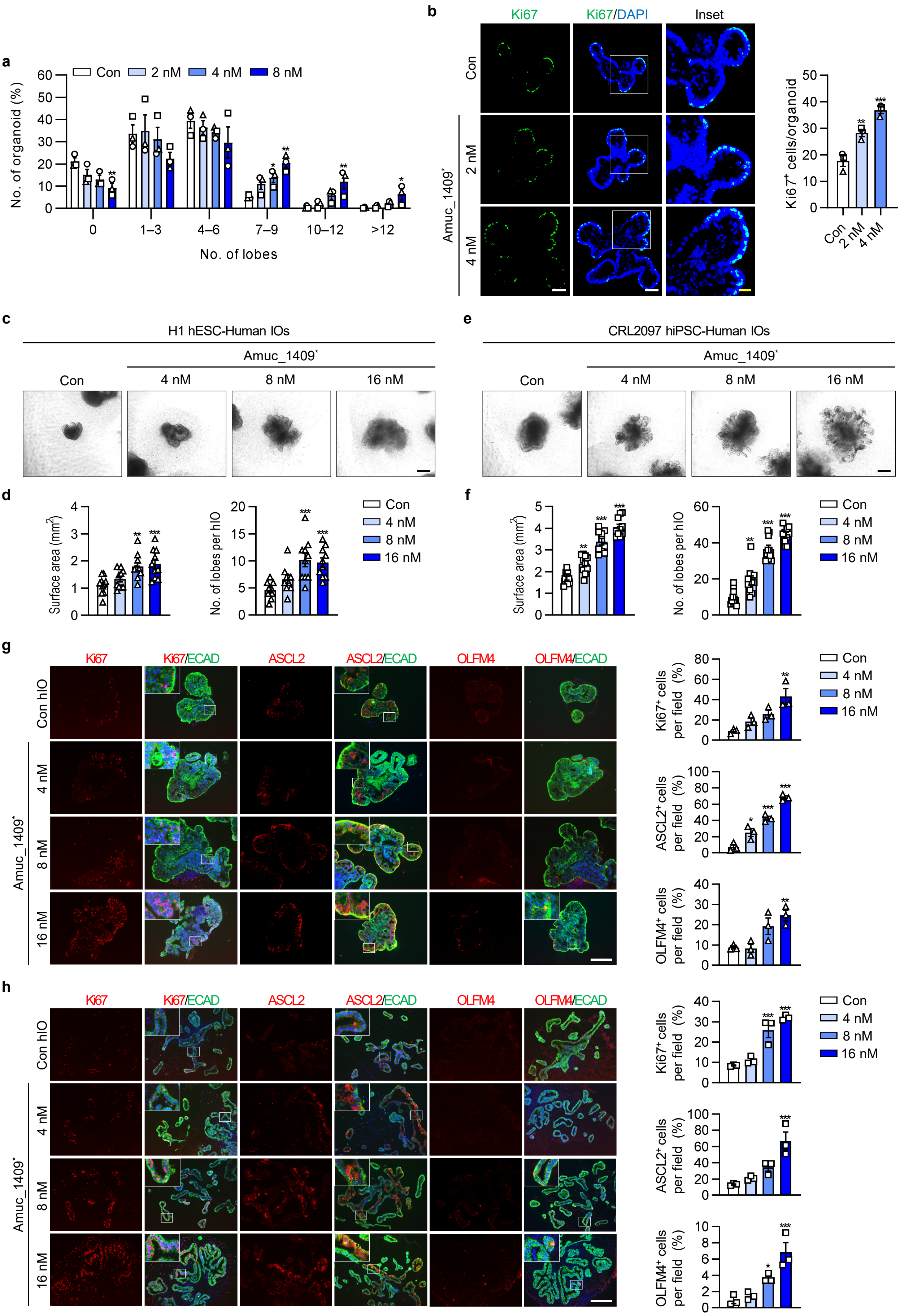

a
Con
2 nM
4 nM
8 nM
No. of organoid (%)
No. of lobes
0
1–3
4–6
7–9
10–12
>12
b
Ki67
Ki67/DAPI
Inset
Con
2 nM
4 nM
Amuc_1409*
Ki67+ cells/organoid
c
H1 hESC-Human IOs
Amuc_1409*
Con
4 nM
8 nM
16 nM
d
Surface area (mm2)
No. of lobes per hIO
e
CRL2097 hiPSC-Human IOs
f
g
Ki67
Ki67/ECAD
ASCL2
ASCL2/ECAD
OLFM4
OLFM4/ECAD
Con hIO
Ki67+ cells per field (%)
ASCL2+ cells per field (%)
OLFM4+ cells per field (%)
h

Supplementary Fig. 1 Amuc_1409 improves the proliferation and development of intestinal organoids. a The percentage distribution of organoids with the indicated number of lobes per mIO in control and Amuc_1409*-treated young mIOs. **b** Representative IF staining images (left panel) for Ki67 (green) and DAPI (nuclei, blue), and quantification of $Ki67^+$ cells per organoid in control and Amuc_1409*-treated young mIOs. White scale bar, 50 µm. Yellow scale bar, 20 µm. **c**, **d** Representative brightfield images (**c**), the surface area of hIOs (**d**, left panel), and the number of lobes per hIO (**d**, right panel) in control and Amuc_1409*-treated hIOs derived from H1 hESC line. n = 10 organoids per group. Scale bar, 200 µm. **e**, **f** Representative brightfield images (**e**), the surface area of hIOs (**f**, left panel), and the number of lobes per hIO (**f**, right panel) in control and Amuc_1409*-treated hIOs derived from CRL2097 hiPSC line. n = 10 organoids per group. Scale bar, 200 µm. **g**, **h** Representative IF staining images (**g**, **h**, left panel) for the proliferation marker (Ki67), intestinal stem cell marker (ASCL2), and intestinal maturation marker (OLFM4) in control hIOs and Amuc_1409*-treated hIOs derived from H1 hESC line (**g**) or CRL2097 hiPSC line (**h**). Quantification of the percentage of positive cells for each marker per field of view is presented (**g**, **h**, right panel, n = 3 fields per group). Intestinal epithelial cells were identified using E-cadherin (ECAD, green); Ki67, ASCL2, and OLFM4 (red); DAPI (blue). Scale bar, 275 µm. All data are presented as the mean ± SEM. In **a** and **b**, a different symbol indicates a data point representing each biological replicate from independently established organoid lines derived from distinct mouse litters (n = 3 biologically independent mice). In **b**, $Ki67^+$ cells were counted in 20 organoids per group from each biological replicate. Statistical analyses were performed via one-way ANOVA with Dunnett's multiple comparisons test (**a**, **b**, **d**, **f**, **g**, **h**) (*$p < 0.05$, **$p < 0.01$, and ***$p < 0.001$ vs control group). Source data, including the exact p-values, are provided as a Source Data file.

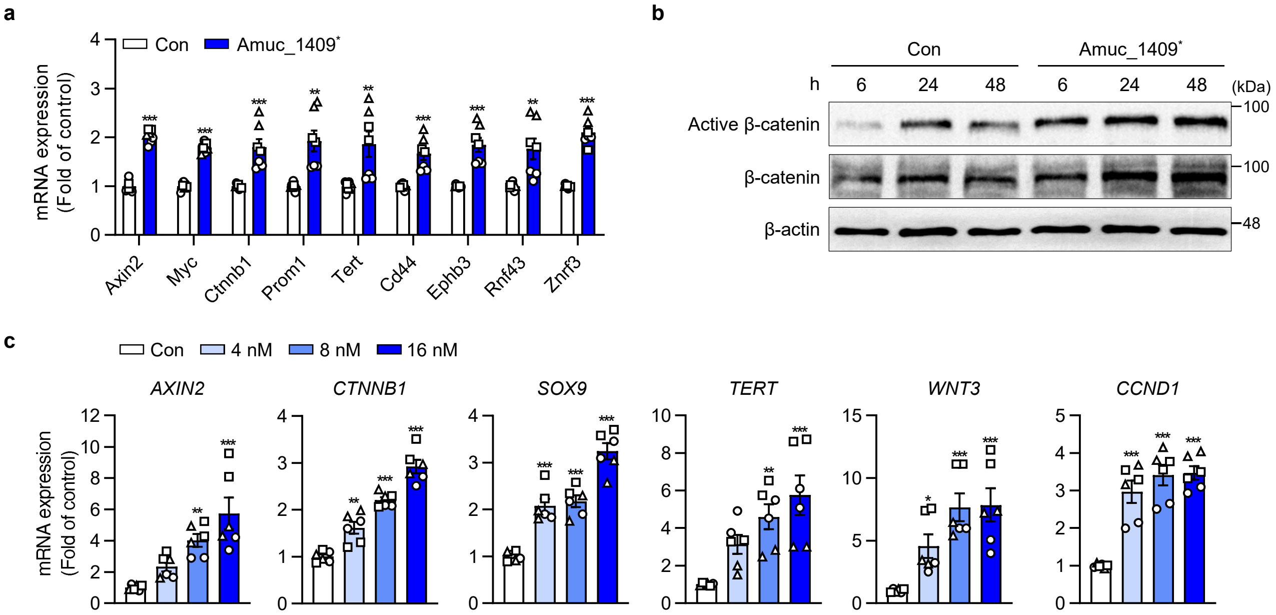

Supplementary Fig. 2 Amuc_1409 activates the Wnt/β-catenin signaling pathway in intestinal organoids. a qRT-PCR showing the relative mRNA expression of Wnt/β-catenin target genes in mIOs treated with or without Amuc_1409* (8 nM). **b** Western blot results showing the protein levels of active and total β-catenin in mIOs treated with or without Amuc_1409* (8 nM) for the indicated times point before harvest. **c** qRT-PCR results showing the relative mRNA expression of Wnt/β-catenin target genes in control hIOs and hIOs treated with the indicated concentration of Amuc_1409* (4, 8, and 16 nM). All data are presented as the mean ± SEM. In **a** and **c**, a different symbol indicates a data point representing each biological replicate from independently established organoid lines derived from distinct mouse litters (**a**: n = 3 biologically independent mice) or two hESC/one hiPSC lines (**c**: n = 3 biologically independent organoid lines). Each biological replicate includes two or three technical replicates. In **b**, data shown are representative of two independent biological replicates, each with similar results. Statistical analyses were performed using two-tailed Student's t-test (**a**) and one-way ANOVA with Dunnett's multiple comparisons test (**c**) (*$p < 0.05$, **$p < 0.01$, and ***$p < 0.001$ vs control group). Source data, including the exact p-values, are provided as a Source Data file. The uncropped western blot images are provided in Supplementary Fig. 13.

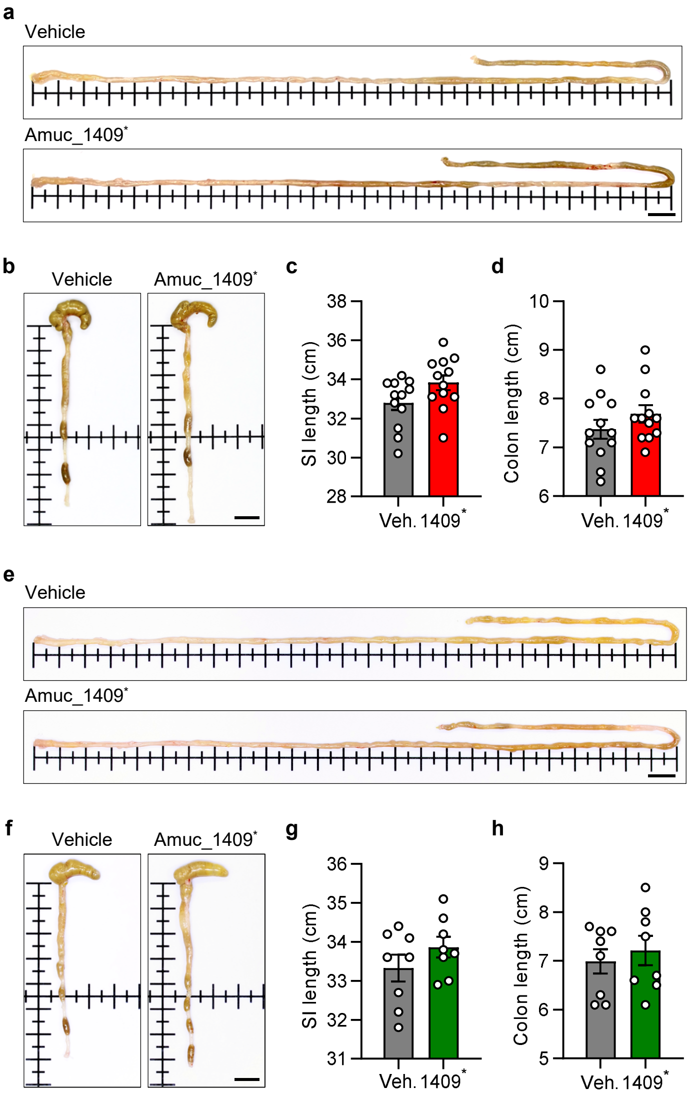

Supplementary Fig. 3 The effects of Amuc_1409 administration on changes of intestinal length in irradiated or 5-FU-treated mice. a–d Results of gross morphological analyses of the SI and colon from vehicle- or Amuc_1409*-treated mice on day 5 after irradiation (n = 12 biological replicate mice per group). Representative macroscopic images of the SI (**a**) and colon (**b**) and quantified length of the SI (**c**) and colon (**d**). Scale bar, 1 cm. **e**–**h** Results of the gross morphological analyses of the SI and colon from vehicle- or Amuc_1409*-treated mice in the 5-FU-induced intestinal damage model (n = 8 biological replicate mice per group). Representative macroscopic images of the SI (**e**) and colon (**f**) and quantified length of the SI (**g**) and colon (**h**). Scale bar, 1 cm. All data are presented as the mean ± SEM. Data shown in **a**–**h** are representative of three independent experiments, each with similar results. Each data point represents a biological replicate, corresponding to one mouse. Statistical analyses were performed using two-tailed Student's *t*-test (**c**, **d**, **g**, **h**). Source data, including the exact *p*-values, are provided as a Source Data file.

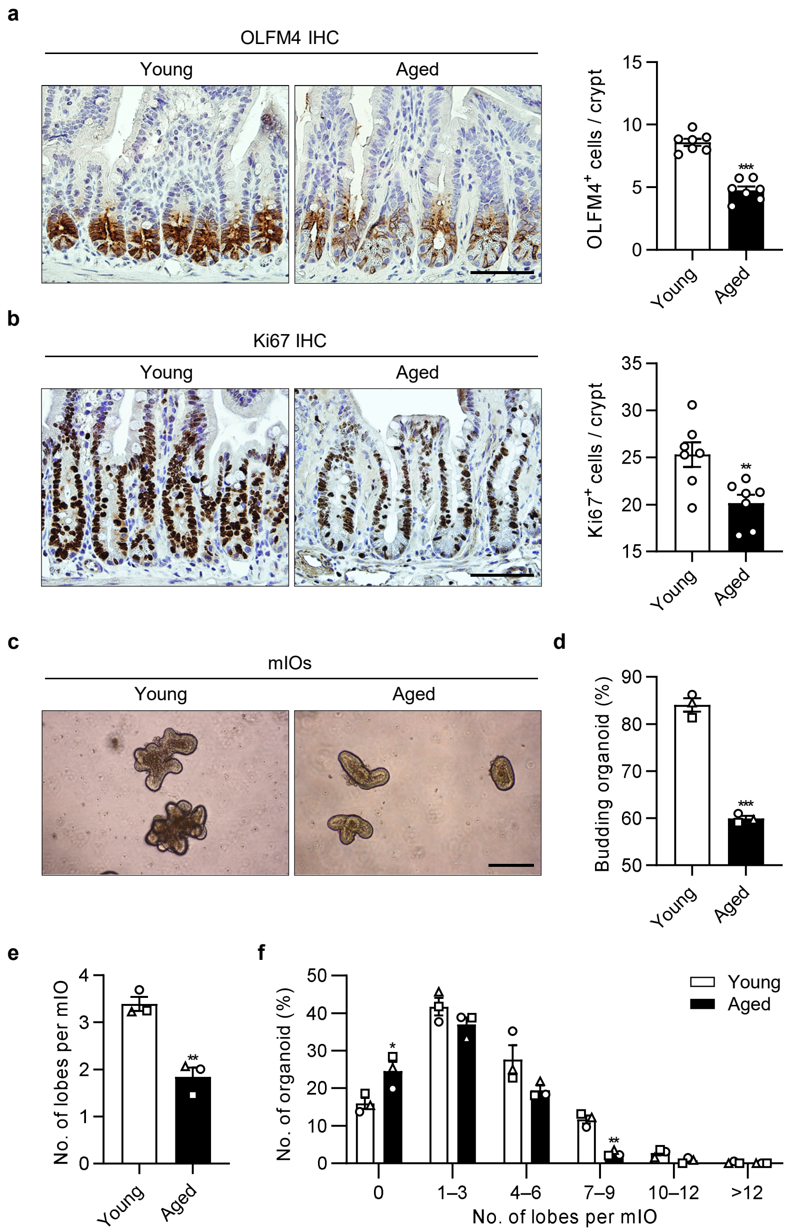

Supplementary Fig. 4 Aging induces a decline in the number and function of ISCs. **a**, **b** Comparison of the ISC number and ISC and TA cell proliferation between small intestine (SI) tissue of young (3–4-month-old) and aged (25-month-old) mice via IHC. Representative IHC images for OLFM4 (**a**, left panel) and Ki67 (**b**, left panel), and quantification of OLFM4^{+} cells (**a**, right panel) and Ki67^{+} cells per crypt (**b**, right panel). Scale bar, 50 μm. **c**–**f**, Comparison of the regenerative capacity of ISC in mIOs formed from SI crypts of young (3–4-month-old) and aged (25-month-old) mice. Representative brightfield images (**c**) and assessment of the percentage of budding organoids (**d**), the number of lobes per mIO (**e**), and the percentage distribution of organoids with indicated number of lobes per mIO (**f**) in the young and aged mIOs on day 4 after the second subculturing passage. Scale bar, 200 μm. In **a** and **b**, each data point represents a biological replicate, corresponding to one mouse (n = 7 biological replicate mice per group). In **d**–**f**, a different symbol indicates a data point representing each biological replicate from independently established organoid lines derived from distinct mouse litters (n = 3 biological replicate mice per group). All data are presented as the mean ± SEM. Statistical analyses were performed using two-tailed Student's t-test (*$p < 0.05$, **$p < 0.01$, and ***$p < 0.001$ vs young group). Source data, including the exact p-values, are provided as a Source Data file.

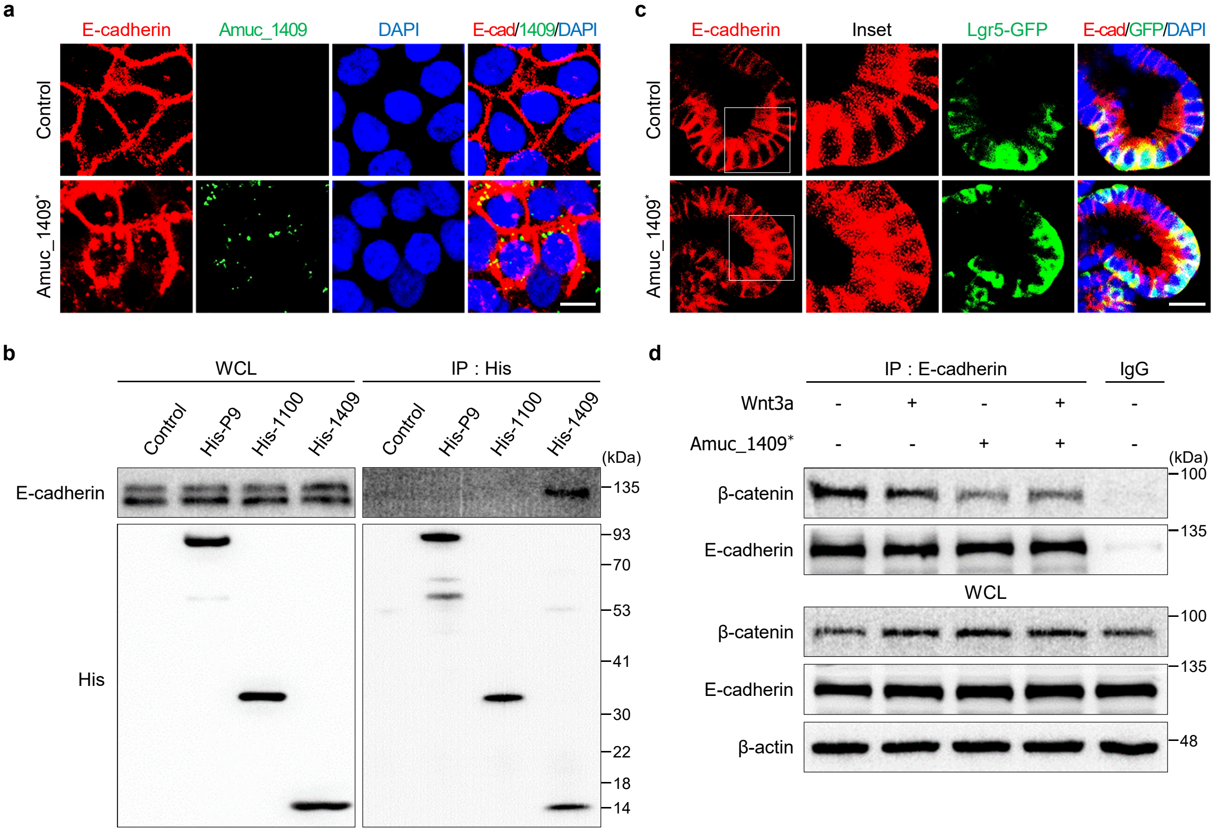

Supplementary Fig. 5 Amuc_1409 triggers E-cadherin/β-catenin complex dissociation through interaction with E-cadherin. a Representative images of IF staining for E-cadherin and Amuc_1409 in HT-29 cells treated with or without Amuc_1409* for 30 min. E-cadherin (red); Amuc_1409 (green), DAPI (nuclei, blue). Scale bar, 10 µm. **b** Results after WCLs from HT-29 cells incubated with purified His-tagged P9, His-tagged Amuc_1100 or His-tagged Amuc_1409 protein (0.5 µg) for 1 h were subjected to immunoprecipitation using His-antibodies, followed by immunoblotting with the indicated antibodies. His-tagged P9 and His-tagged Amuc_1100 proteins were used as control proteins. **c** Representative images of IF staining for E-cadherin and GFP in vehicle- or Amuc_1409* (16 nM)-treated mIOs derived from *Lgr5-EGFP-IRES-CreERT2* mice. E-cadherin (red); Lgr5-GFP^{+} ISC (green), DAPI (nuclei, blue). Scale bar, 20 µm. **d** HT-29 cells were treated with Wnt3a (150 ng/mL), Amuc_1409* (16 nM), or a combination of Wnt3a (150 ng/mL) and Amuc_1409* (16 nM) for 30 min and then, they were harvested. WCLs were prepared to perform immunoprecipitation with E-cadherin, followed by immunoblotting with the indicated antibodies. Data shown in **a**–**d** are representative of two independent experiments, each with similar results. The uncropped western blot images are provided in Supplementary Fig. 14.

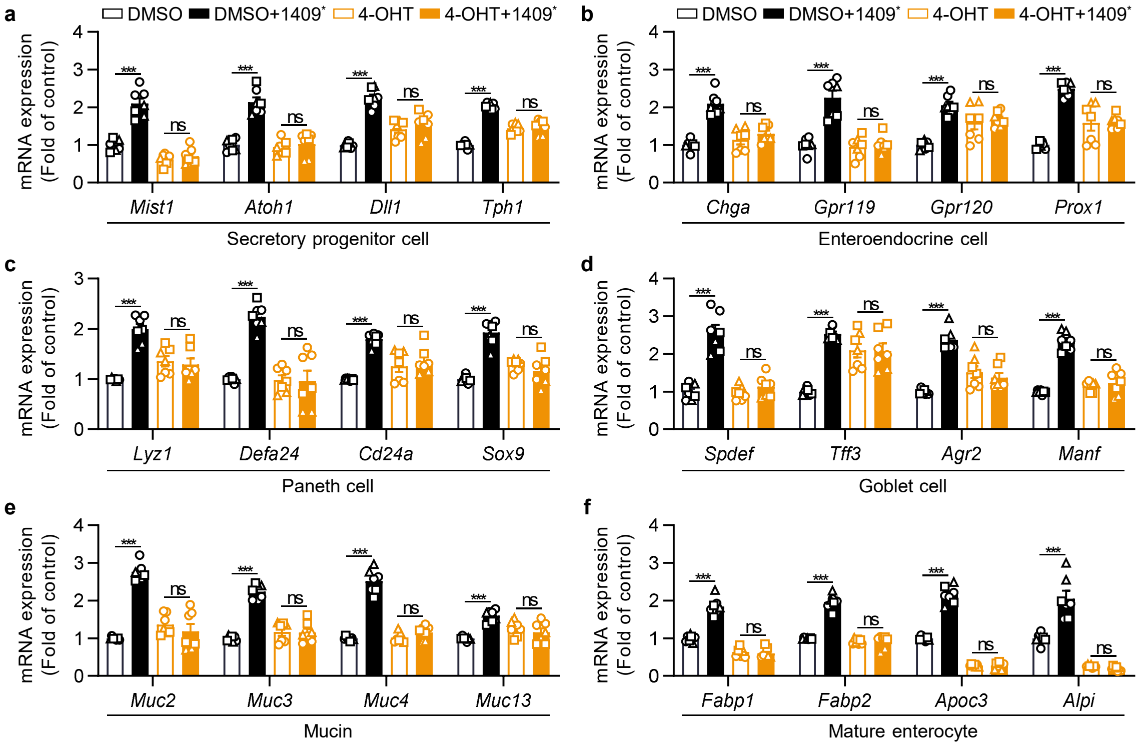

Supplementary Fig. 6 Amuc_1409 promotes ISC-mediated epithelial differentiation in an E-cadherin-dependent manner. a–**f** qRT-PCR results showing the relative mRNA expression of secretory progenitor cell markers (**a**), enteroendocrine cell markers (**b**), Paneth cell markers (**c**), goblet cell markers (**d**), mucin markers (**e**), and mature enterocyte markers (**f**) in mIOs from *Lgr5-CreERT2;Cdh1*$^{fl/fl}$ mice treated with or without Amuc_1409* (16 nM) at day 5 after either DMSO (vehicle control) or 4-OHT (1 μM) addition began. All data are presented as the mean ± SEM. A different symbol indicates a data point representing each biological replicate from independently established organoid lines derived from distinct mouse litters (n = 3 biologically independent mice). Each biological replicate includes two or three technical replicates. Statistical analyses were performed using one-way ANOVA with Dunnett's multiple comparisons test (**a**–**f**) (ns, not statistically significant, ***p < 0.001 vs control group). Source data, including the exact p-values, are provided as a Source Data file.

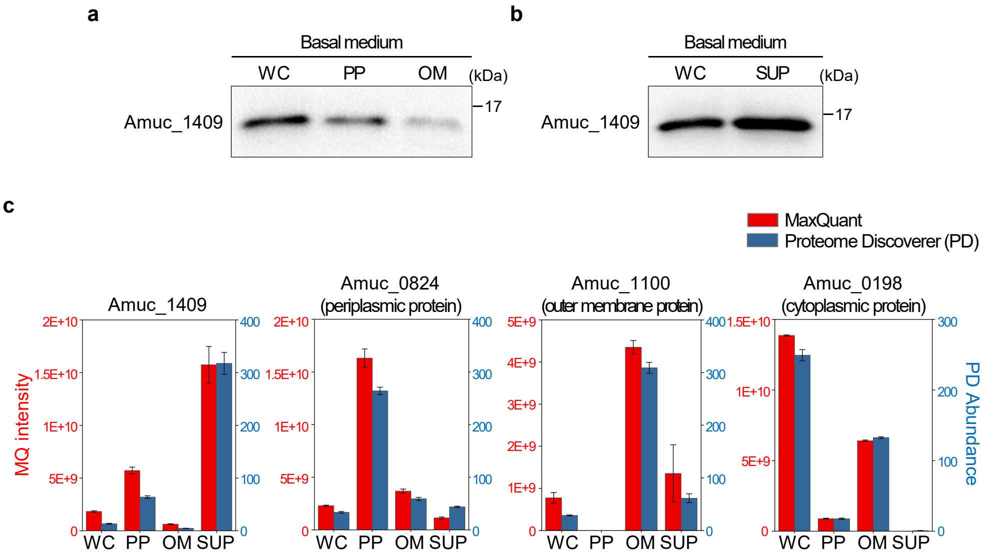

Supplementary Fig. 7 Subcellular localization of Amuc_1409 in *A. muciniphila*. *A. muciniphila* BAA-835^T was grown in basal medium under anaerobic conditions at 37°C for 48 h and cell pellets and supernatants were collected from this culture. **a** Western blot analysis of Amuc_1409 in whole cell lysate (WC), periplasmic (PP), and outer membrane (OM) fractions, loaded with 5 µg protein per fraction sample. **b** Western blot analysis of Amuc_1409 secreted into the cell-free culture supernatant (SUP). **c** Relative quantitative analysis of Amuc_1409, Amuc_0824 (PP protein), Amuc_1100 (OM protein), and Amuc_0198 (cytoplasmic protein) detected in four samples (WC, PP, OM, and SUP) were carried out via two MS1-based label-free quantitation (LFQ) methods (i. MaxQuant, MQ; ii. Proteome Discoverer Ver 3.0, PD). LFQ-based profiling of four proteins (Amuc_1409, Amuc_0824, Amuc_1100, and Amuc_0198) was conducted using the MQ (shown in red) and PD with Minora tool (shown in blue) and plotted on a bar chart with two Y-axes (MQ Intensity, left; PD Abundance, right). The relative abundance was calculated as the intensity ratio of each protein to the total protein in each fraction sample. All data are presented as the mean ± SEM. Source data are provided as a Source Data file. The uncropped western blot images are provided in Supplementary Fig. 15.

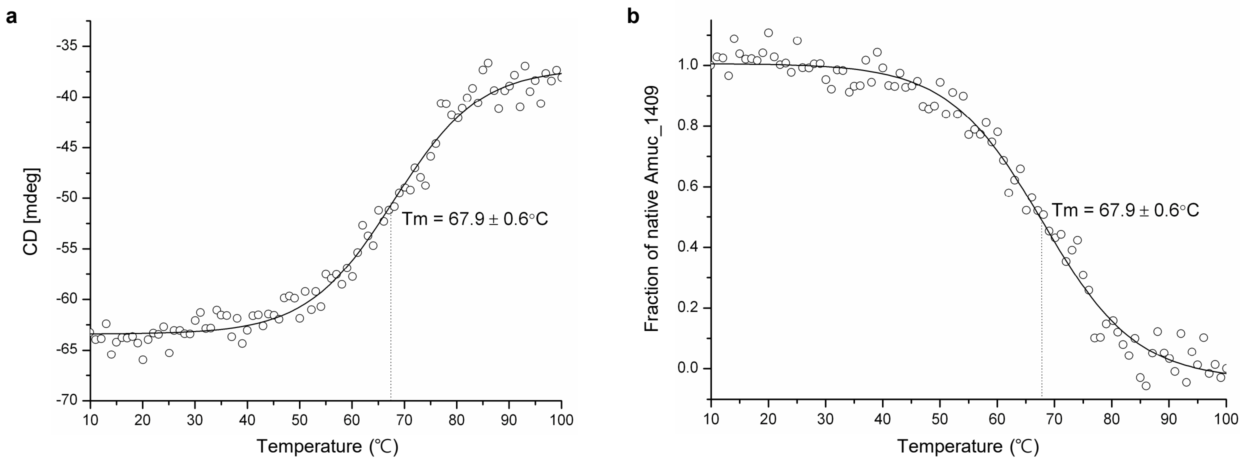

Supplementary Fig. 8 Thermal unfolding of the Amuc_1409 protein as monitored with circular dichroism. a, **b** Thermal unfolding profile of raw circular dichroism (CD) spectra (**a**) and a normalized two-state unfolding model (**b**) of Amuc_1409 protein by the standard equation: Fraction of native Amuc_1409 = $(\theta_t - \theta_{100})/(\theta_{10} - \theta_{100})$. The CD spectra were monitored at 216 nm by raising the temperature in 1°C intervals from 10 to 100°C. The melting temperature (T_m, 67.9 ± 0.6°C) was calculated by a non-linear curve fit of Boltzmann method using Origin-v8.0 program (Origin Lab Corporation, USA). Source data are provided as a Source Data file.

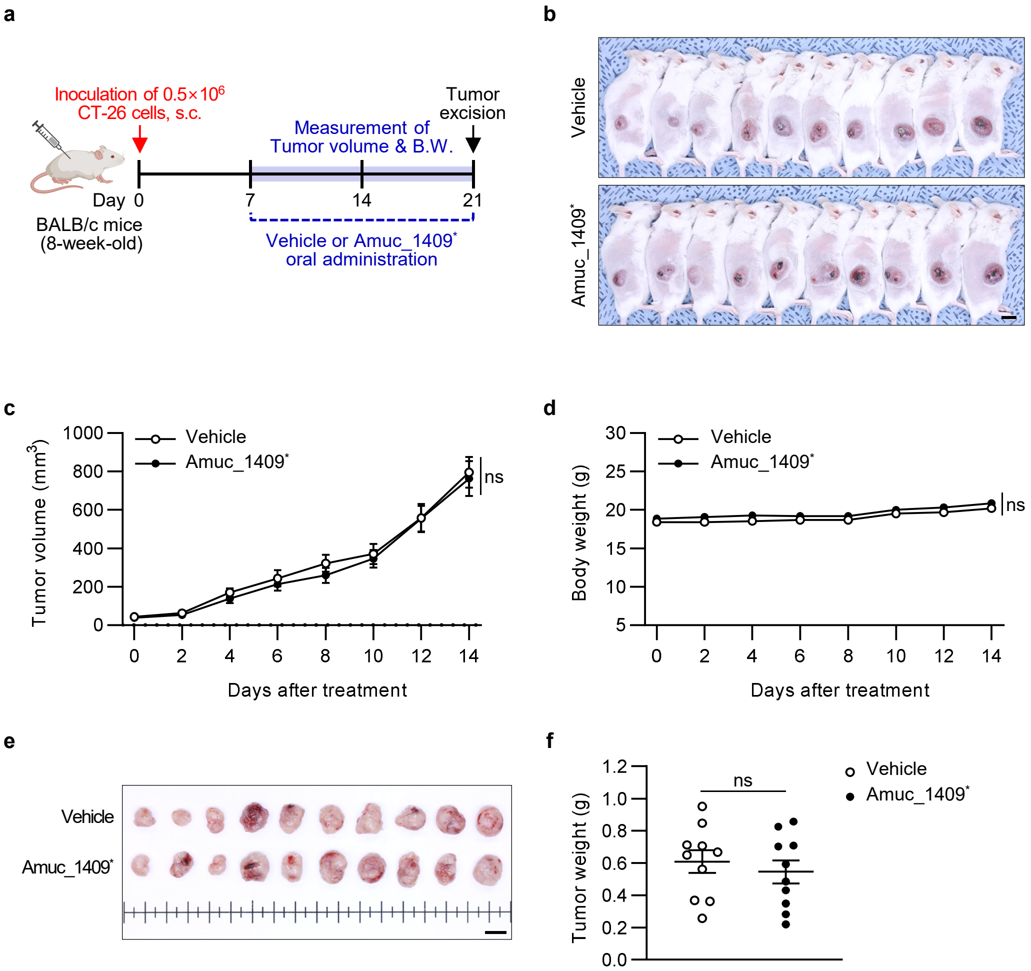

Supplementary Fig. 9 Amuc_1409 showed no obvious effect on tumor proliferation in the CT-26 syngeneic mouse model. a Scheme showing the experimental workflow for oral administration of vehicle or Amuc_1409* (9 µg per mouse) for 14 days, starting from 7 days after the subcutaneous (s.c.) injection of CT-26 cells in BALB/c mice (*n* = 10 biological replicate mice per group). Images were created with biorender.com and have been granted a publication license. **b** Gross observation of CT-26 tumor-bearing mice on day 14 after treatment with vehicle or Amuc_1409*. Scale bar, 1 cm. **c**, **d** The changes in the tumor volume (**c**) and body weight (**d**) during the course of treatment. **e**, **f** Gross observation (**e**) and weight (**f**) of excised tumor from CT-26 tumor-bearing mice sacrificed on day 14 days after treatment with vehicle or Amuc_1409*. Scale bar, 1 cm. In **f**, each data point represents a biological replicate, corresponding to one mouse. All data are presented as the mean ± SEM. Data shown are representative of three independent experiments, each with similar results. Statistical analyses were performed using two-tailed Student's *t*-test (**c**, **d**, **f**) (ns, not statistically significant, vs vehicle-treated group). Source data, including the exact *p*-values, are provided as a Source Data file.

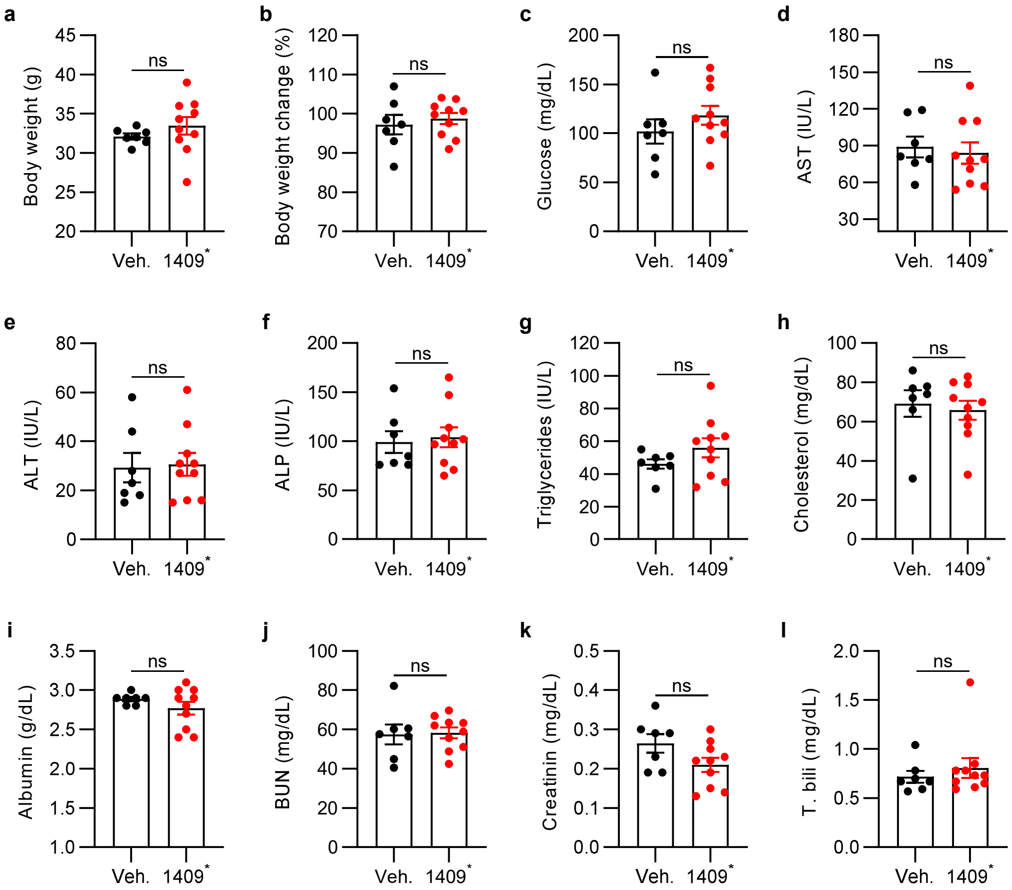

Supplementary Fig. 10 Long-term administration of Amuc_1409 has no adverse effects on body weight and blood chemistry values in a natural aging mouse model. a–l Bar graphs showing the absolute body weight (**a**), the percentage change in body weight (**b**), and blood chemistry values (**c**–**l**) of each group at 15 weeks after vehicle (n = 7 biological replicate mice) or Amuc_1409* (n = 10 biological replicate mice, 4.5 µg per mouse) administration in the natural aging mice (100-week-old) model. AST (aspartate aminotransferase), ALT (alanine aminotransferase), ALP (alkaline phosphatase), BUN (blood urea nitrogen), T. bili (total bilirubin). All data are presented as the mean ± SEM. Data shown are representative of three independent experiments, each with similar results. Each data point represents a biological replicate, corresponding to one mouse. Statistical analyses were performed using two-tailed Student's t-test. (ns, not statistically significant, vs vehicle-treated group). Source data, including the exact p-values, are provided as a Source Data file.

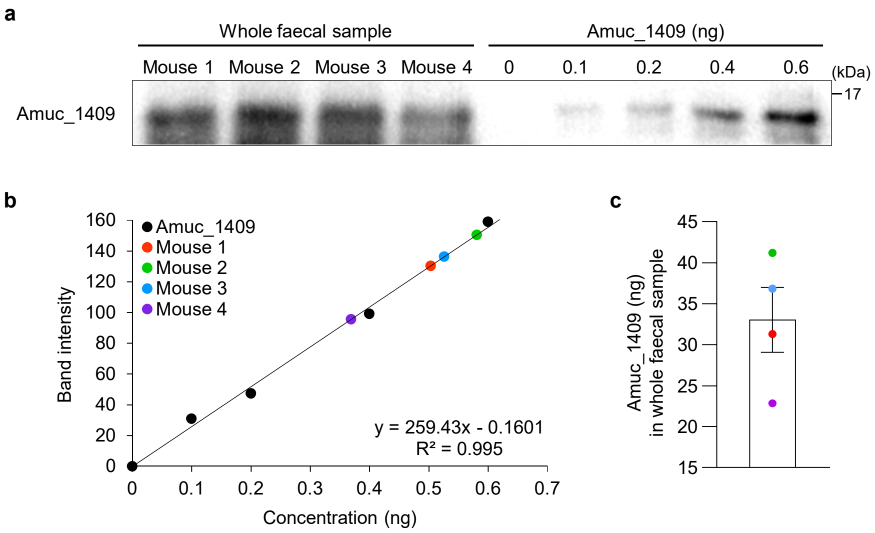

Supplementary Fig. 11 The quantification of physiological concentration of Amuc_1409 in whole faecal samples from the mouse gut. a Proteins (100 µg) isolated from whole faecal samples of mouse gut (*n* = 4 biological replicate mice) were subjected to western blotting in parallel with dilution of purified Amuc_1409 protein with known concentration and detected using an in-house-made rabbit polyclonal antibody for Amuc_1409. The signal intensity of each band was measured using ImageJ software. **b** Standard curve for Amuc_1409 protein was generated using band intensities of different dilutions of purified Amuc_1409 protein. The physiological concentration of Amuc_1409 in whole faecal samples was calculated by inserting the sample's band intensity into the standard curve. **c** Bar graphs representing mean values of estimated physiological concentration of Amuc_1409 in whole faecal samples of mouse gut. All data are presented as the mean ± SEM. Source data are provided as a Source Data file. The uncropped western blot image is provided in Supplementary Fig. 16.

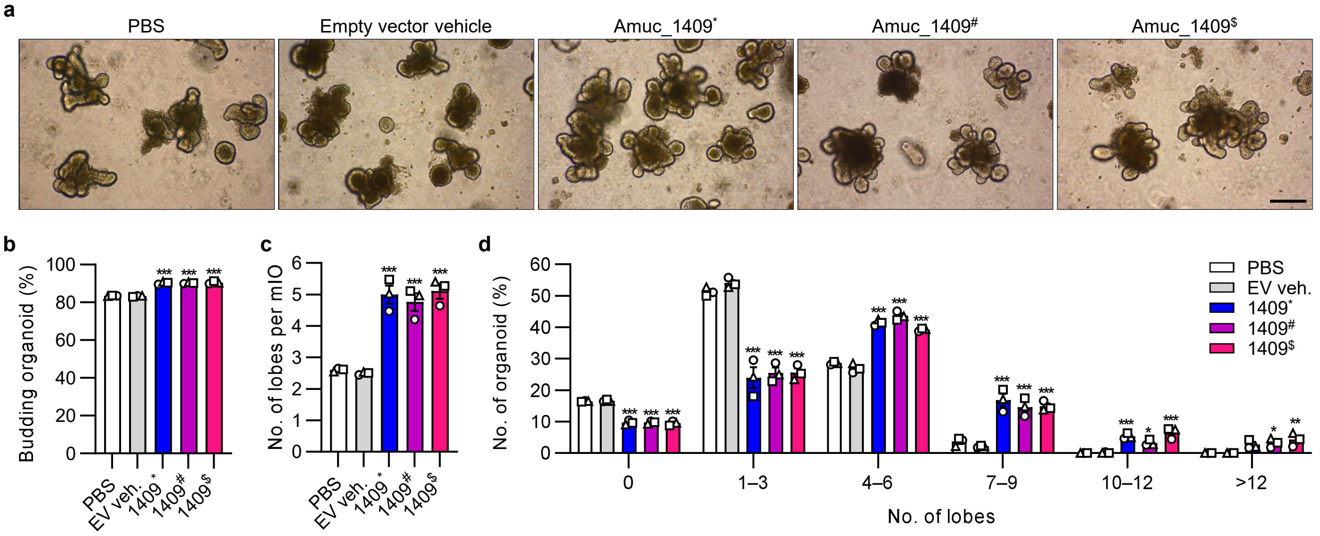

Supplementary Fig. 12 The promotion of organoid growth is specifically attributed to the Amuc_1409 protein itself, not the His-tag or potential contaminant carryover. a–d Analysis of mIOs treated with PBS, empty vector vehicle, Amuc_1409*, Amuc_1409$^{\#}$, and Amuc_1409$^{\$}$ on day 4 after the second subculturing passage. Empty vector vehicle (EV veh.) is the solution obtained from the same purification process of EV-transformed *E. coli*. Amuc_1409* is the recombinant His-tagged Amuc_1409 protein expressed in *E. coli* and purified using NI-NTA affinity chromatography. Amuc_1409$^{\#}$ is the recombinant His-tag cleaved Amuc_1409 protein by the TEV protease. Amuc_1409$^{\$}$ is the recombinant His-tag cleaved Amuc_1409 protein, which underwent an additional purification step through gel filtration chromatography. Representative brightfield images of the mIOs (**a**), quantitative assessment of the percentage of budding organoids (**b**), the number of lobes per mIO (**c**), and the percentage distribution of organoids with the indicated number of lobes per mIO (**d**) in mIOs. Scale bar, 200 μm. All data are presented as the mean ± SEM. A different symbol indicates a data point representing each biological replicate from independently established organoid lines derived from distinct mouse litters (n = 3 biologically independent mice). Statistical analyses were performed using one-way ANOVA with Dunnett's multiple comparisons test (*$p < 0.05$, **$p < 0.01$, and ***$p < 0.001$ vs PBS-treated group). Source data, including the exact p-values, are provided as a Source Data file.

Supplementary Fig. 2b

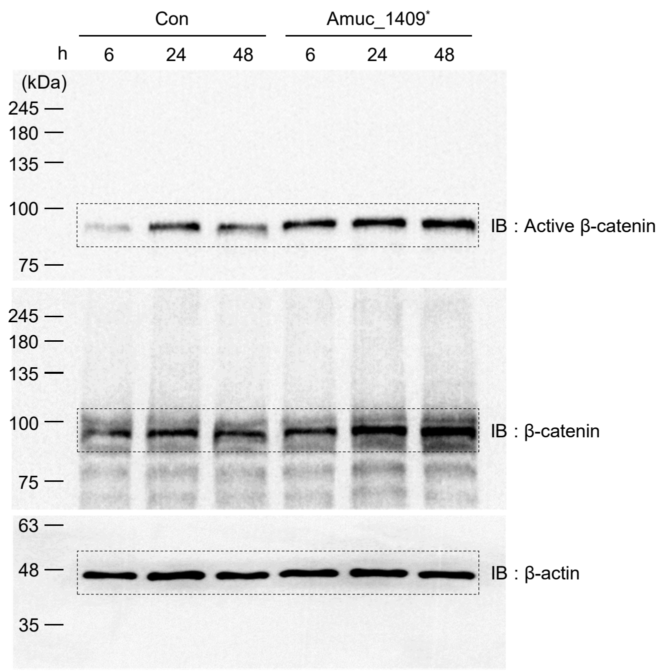

Supplementary Fig. 13 The uncropped western blot images presented in the indicated figures.

Note: Black dashed lines identify the cropped areas

Supplementary Fig. 5b

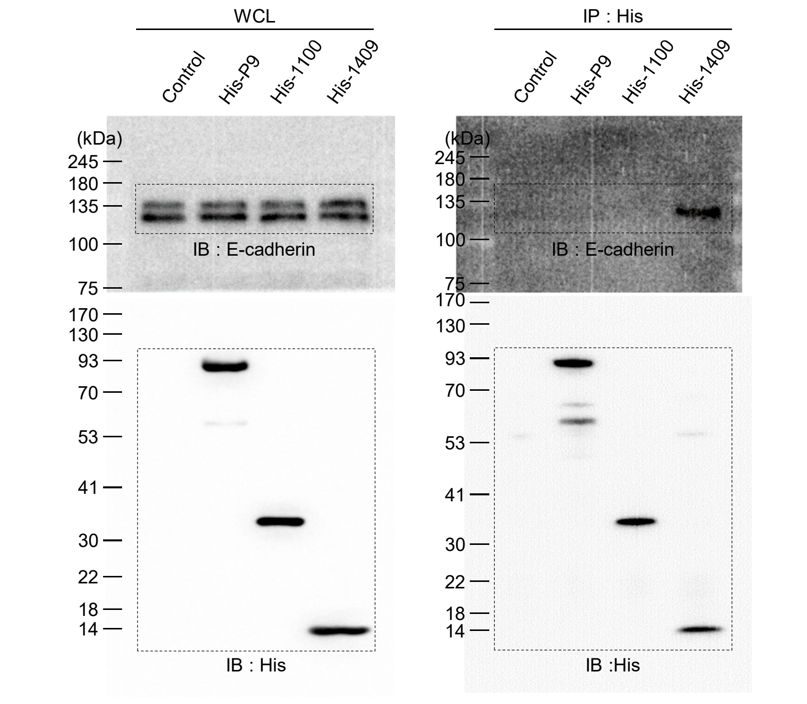

Supplementary Fig. 5d

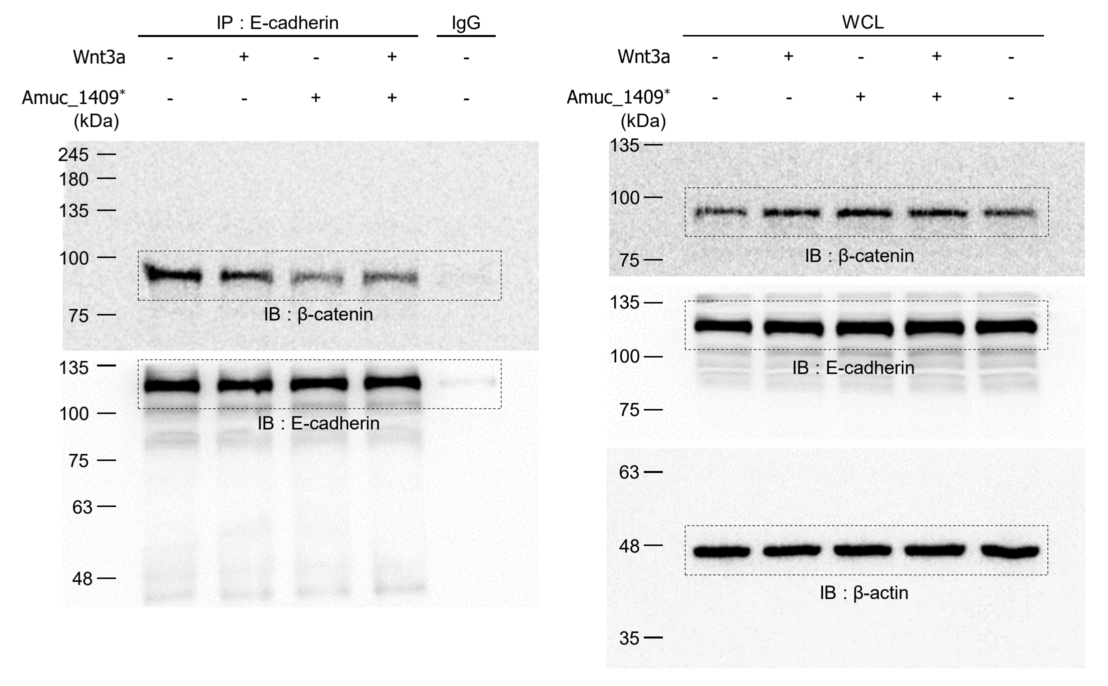

Supplementary Fig. 14 The uncropped western blot images presented in the indicated figures.

Note: Black dashed lines identify the cropped areas

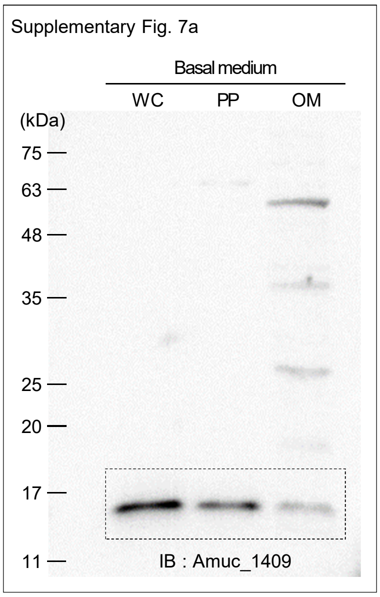

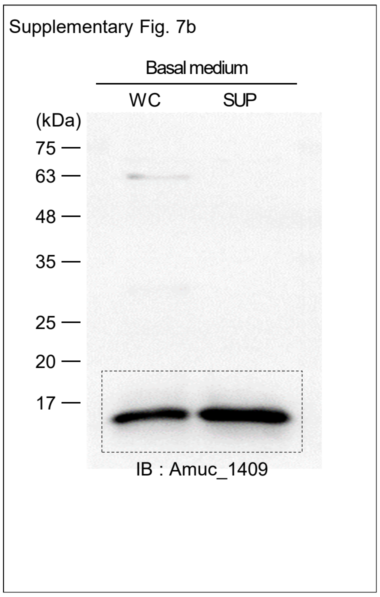

Supplementary Fig. 15 The uncropped western blot images presented in the indicated figures.

Note: Black dashed lines identify the cropped areas

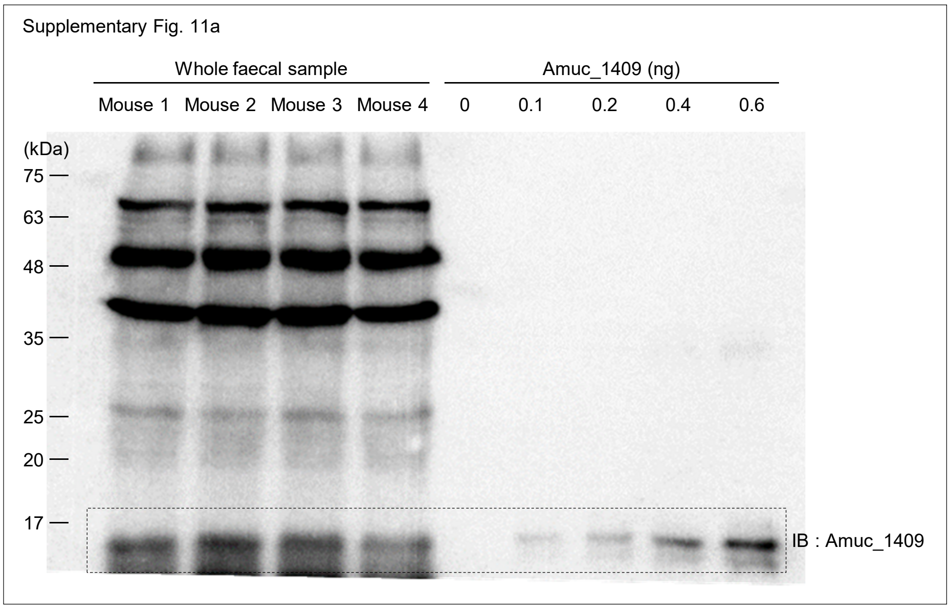

Supplementary Fig. 16 The uncropped western blot images presented in the indicated figures.

Note: Black dashed lines identify the cropped areas

Supplementary Table 1. List of proteins identified in secretome of *A. muciniphila* cultivated under under basal medium.

Majority protein IDs	Fasta headers	Gene locus	Peptides	Unique peptides	Sequence coverage [%]	Mol. weight [kDa]	Sequence lengths	Score	Intensity	MS/MS count	iBAQ
B2UKW7	tr|B2UKW7|B2UKW7_AKKM8 Glyceraldehyde-3-phosphate dehydrogenase OS=Akkermansia muciniphila (strain ATCC BAA-835 / DSM 22959 / JCM 33894 / BCRC 81048 / CCUG 64013 / CIP 107961 / Muc) OX=349741 GN=Amuc_1417 PE=3 SV=1	Amuc_1417	21	21	68.00	36.74	341	323.31	5.E+11	3106	2.E+10
B2UQS2	sp|B2UQS2|ACP_AKKM8 Acyl carrier protein OS=Akkermansia muciniphila (strain ATCC BAA-835 / DSM 22959 / JCM 33894 / BCRC 81048 / CCUG 64013 / CIP 107961 / Muc) OX=349741 GN=acpP PE=3 SV=1	Amuc_0975	3	3	40.00	8.82	80	200.16	6.E+10	1971	2.E+10
B2UP94	tr|B2UP94|B2UP94_AKKM8 Rubrerythrin domain-containing protein OS=Akkermansia muciniphila (strain ATCC BAA-835 / DSM 22959 / JCM 33894 / BCRC 81048 / CCUG 64013 / CIP 107961 / Muc) OX=349741 GN=Amuc_2055 PE=4 SV=1	Amuc_2055	7	5	86.00	12.73	114	264.75	1.E+11	1261	2.E+10
B2UMR7	tr|B2UMR7|B2UMR7_AKKM8 Histone family protein DNA-binding protein OS=Akkermansia muciniphila (strain ATCC BAA-835 / DSM 22959 / JCM 33894 / BCRC 81048 / CCUG 64013 / CIP 107961 / Muc) OX=349741 GN=Amuc_0281 PE=3 SV=1	Amuc_0281	4	4	50.50	11.25	101	210.98	6.E+10	587	2.E+10
B2UQP3	tr|B2UQP3|B2UQP3_AKKM8 DJ-1 family protein OS=Akkermansia muciniphila (strain ATCC BAA-835 / DSM 22959 / JCM 33894 / BCRC 81048 / CCUG 64013 / CIP 107961 / Muc) OX=349741 GN=Amuc_0946 PE=4 SV=1	Amuc_0946	11	11	67.90	20.11	187	323.31	1.E+11	1832	1.E+10
B2ULM5	tr|B2ULM5|B2ULM5_AKKM8 Superoxide dismutase OS=Akkermansia muciniphila (strain ATCC BAA-835 / DSM 22959 / JCM 33894 / BCRC 81048 / CCUG 64013 / CIP 107961 / Muc) OX=349741 GN=Amuc_1592 PE=3 SV=1	Amuc_1592	11	11	56.10	23.92	214	323.31	2.E+11	1249	1.E+10
B2URC4	tr|B2URC4|B2URC4_AKKM8 Enolase OS=Akkermansia muciniphila (strain ATCC BAA-835 / DSM 22959 / JCM 33894 / BCRC 81048 / CCUG 64013 / CIP 107961 / Muc) OX=349741 GN=eno PE=3 SV=1	Amuc_1184	19	17	67.10	45.68	426	323.31	3.E+11	3686	1.E+10
B2UPZ3	tr|B2UPZ3|B2UPZ3_AKKM8 Thioredoxin OS=Akkermansia muciniphila (strain ATCC BAA-835 / DSM 22959 / JCM 33894 / BCRC 81048 / CCUG 64013 / CIP 107961 / Muc) OX=349741 GN=Amuc_0691 PE=3 SV=1	Amuc_0691	5	5	66.70	11.49	105	237.29	6.E+10	502	1.E+10
B2URF0	tr|B2URF0|B2URF0_AKKM8 Phosphoenolpyruvate carboxykinase [GTP] OS=Akkermansia muciniphila (strain ATCC BAA-835 / DSM 22959 / JCM 33894 / BCRC 81048 / CCUG 64013 / CIP 107961 / Muc) OX=349741 GN=pckG PE=3 SV=1	Amuc_1210	22	22	48.40	67.52	608	323.31	2.E+11	2108	8.E+09
B2UN99	tr|B2UN99|B2UN99_AKKM8 Glutamate decarboxylase OS=Akkermansia muciniphila (strain ATCC BAA-835 / DSM 22959 / JCM 33894 / BCRC 81048 / CCUG 64013 / CIP 107961 / Muc) OX=349741 GN=Amuc_0372 PE=3 SV=1	Amuc_0372	24	24	65.90	52.46	466	323.31	2.E+11	2064	7.E+09
B2UKW8	sp|B2UKW8|PGK_AKKM8 Phosphoglycerate kinase OS=Akkermansia muciniphila (strain ATCC BAA-835 / DSM 22959 / JCM 33894 / BCRC 81048 / CCUG 64013 / CIP 107961 / Muc) OX=349741 GN=pgk PE=3 SV=1	Amuc_1418	24	24	75.20	42.69	403	323.31	1.E+11	2595	6.E+09
B2UKV9	tr|B2UKV9|B2UKV9_AKKM8 PepSY_like domain-containing protein OS=Akkermansia muciniphila (strain ATCC BAA-835 / DSM 22959 / JCM 33894 / BCRC 81048 / CCUG 64013 / CIP 107961 / Muc) OX=349741 GN=Amuc_1409 PE=4 SV=1	Amuc_1409	8	8	52.00	16.52	150	245.16	4.E+10	538	5.E+09
B2UQ05	tr|B2UQ05|B2UQ05_AKKM8 Alanine--glyoxylate transaminase OS=Akkermansia muciniphila (strain ATCC BAA-835 / DSM 22959 / JCM 33894 / BCRC 81048 / CCUG 64013 / CIP 107961 / Muc) OX=349741 GN=Amuc_0703 PE=3 SV=1	Amuc_0703	20	20	76.80	39.28	357	323.31	1.E+11	2027	5.E+09
B2UNK0	tr|B2UNK0|B2UNK0_AKKM8 Uncharacterized protein OS=Akkermansia muciniphila (strain ATCC BAA-835 / DSM 22959 / JCM 33894 / BCRC 81048 / CCUG 64013 / CIP 107961 / Muc) OX=349741 GN=Amuc_1902 PE=4 SV=1	Amuc_1902	13	13	71.30	24.93	230	323.31	5.E+10	917	4.E+09
B2UN29	tr|B2UN29|B2UN29_AKKM8 Glucosamine-6-phosphate deaminase OS=Akkermansia muciniphila (strain ATCC BAA-835 / DSM 22959 / JCM 33894 / BCRC 81048 / CCUG 64013 / CIP 107961 / Muc) OX=349741 GN=nagB PE=3 SV=1	Amuc_1822	10	10	46.90	32.84	303	323.31	6.E+10	535	4.E+09
B2UNL9	tr|B2UNL9|B2UNL9_AKKM8 Putative ferredoxin OS=Akkermansia muciniphila (strain ATCC BAA-835 / DSM 22959 / JCM 33894 / BCRC 81048 / CCUG 64013 / CIP 107961 / Muc) OX=349741 GN=Amuc_1922 PE=4 SV=1	Amuc_1922	2	2	24.70	8.40	77	134.67	2.E+10	78	3.E+09

B2UNS8	tr|B2UNS8|B2UNS8_AKKM8 Methylmalonyl-CoA mutase, large subunit OS=Akkermansia muciniphila (strain ATCC BAA-835 / DSM 22959 / JCM 33894 / BCRC 81048 / CCUG 64013 / CIP 107961 / Muc) OX=349741 GN=Amuc_1983 PE=4 SV=1	Amuc_1983	25	25	46.50	78.41	714	323.31	1.E+11	1974	3.E+09
B2UL96	sp|B2UL96|GLSA_AKKM8 Glutaminase OS=Akkermansia muciniphila (strain ATCC BAA-835 / DSM 22959 / JCM 33894 / BCRC 81048 / CCUG 64013 / CIP 107961 / Muc) OX=349741 GN=glsA PE=3 SV=1	Amuc_0038	7	7	31.00	33.13	313	186.41	3.E+10	391	3.E+09
B2UPF0	tr|B2UPF0|B2UPF0_AKKM8 Uncharacterized protein OS=Akkermansia muciniphila (strain ATCC BAA-835 / DSM 22959 / JCM 33894 / BCRC 81048 / CCUG 64013 / CIP 107961 / Muc) OX=349741 GN=Amuc_0590 PE=4 SV=1	Amuc_0590	22	22	51.50	50.45	460	323.31	6.E+10	1665	3.E+09
B2ULM2	tr|B2ULM2|B2ULM2_AKKM8 Aminotransferase OS=Akkermansia muciniphila (strain ATCC BAA-835 / DSM 22959 / JCM 33894 / BCRC 81048 / CCUG 64013 / CIP 107961 / Muc) OX=349741 GN=Amuc_1589 PE=3 SV=1	Amuc_1589	14	14	52.80	42.44	388	323.31	4.E+10	986	3.E+09
B2UNH3	tr|B2UNH3|B2UNH3_AKKM8 Glycine cleavage system H protein OS=Akkermansia muciniphila (strain ATCC BAA-835 / DSM 22959 / JCM 33894 / BCRC 81048 / CCUG 64013 / CIP 107961 / Muc) OX=349741 GN=gcvH PE=3 SV=1	Amuc_0447	3	3	40.50	13.72	126	120.45	5.E+09	111	3.E+09
B2UKY5	sp|B2UKY5|MDH_AKKM8 Malate dehydrogenase OS=Akkermansia muciniphila (strain ATCC BAA-835 / DSM 22959 / JCM 33894 / BCRC 81048 / CCUG 64013 / CIP 107961 / Muc) OX=349741 GN=mdh PE=3 SV=1	Amuc_1436	13	13	47.10	35.10	329	323.31	4.E+10	548	2.E+09
B2URG7	sp|B2URG7|Y1227_AKKM8 Nucleoid-associated protein Amuc_1227 OS=Akkermansia muciniphila (strain ATCC BAA-835 / DSM 22959 / JCM 33894 / BCRC 81048 / CCUG 64013 / CIP 107961 / Muc) OX=349741 GN=Amuc_1227 PE=3 SV=1	Amuc_1227	1	1	13.50	10.91	104	2.56	8.E+09	36	2.E+09
B2UQP5	tr|B2UQP5|B2UQP5_AKKM8 N-acetylglucosamine-6-phosphate deacetylase OS=Akkermansia muciniphila (strain ATCC BAA-835 / DSM 22959 / JCM 33894 / BCRC 81048 / CCUG 64013 / CIP 107961 / Muc) OX=349741 GN=Amuc_0948 PE=3 SV=1	Amuc_0948	12	12	45.30	40.83	382	323.31	4.E+10	614	2.E+09
B2UNS2	tr|B2UNS2|B2UNS2_AKKM8 Glucose-6-phosphate isomerase OS=Akkermansia muciniphila (strain ATCC BAA-835 / DSM 22959 / JCM 33894 / BCRC 81048 / CCUG 64013 / CIP 107961 / Muc) OX=349741 GN=pgi PE=3 SV=1	Amuc_1975	25	25	58.30	55.47	515	323.31	6.E+10	1407	2.E+09
B2UP29	tr|B2UP29|B2UP29_AKKM8 Triosephosphate isomerase OS=Akkermansia muciniphila (strain ATCC BAA-835 / DSM 22959 / JCM 33894 / BCRC 81048 / CCUG 64013 / CIP 107961 / Muc) OX=349741 GN=tpiA PE=3 SV=1	Amuc_0562	10	10	61.40	26.99	254	323.31	2.E+10	517	2.E+09
B2UL55	tr|B2UL55|B2UL55_AKKM8 Malonyl CoA-acyl carrier protein transacylase OS=Akkermansia muciniphila (strain ATCC BAA-835 / DSM 22959 / JCM 33894 / BCRC 81048 / CCUG 64013 / CIP 107961 / Muc) OX=349741 GN=Amuc_1507 PE=3 SV=1	Amuc_1507	9	9	41.90	32.57	308	323.31	3.E+10	444	2.E+09
B2URK9	tr|B2URK9|B2URK9_AKKM8 Thioredoxin reductase OS=Akkermansia muciniphila (strain ATCC BAA-835 / DSM 22959 / JCM 33894 / BCRC 81048 / CCUG 64013 / CIP 107961 / Muc) OX=349741 GN=Amuc_1269 PE=3 SV=1	Amuc_1269	8	8	31.70	33.21	309	248.71	2.E+10	506	2.E+09
B2UPR8	tr|B2UPR8|B2UPR8_AKKM8 Nitroreductase OS=Akkermansia muciniphila (strain ATCC BAA-835 / DSM 22959 / JCM 33894 / BCRC 81048 / CCUG 64013 / CIP 107961 / Muc) OX=349741 GN=Amuc_2137 PE=3 SV=1	Amuc_2137	11	11	49.30	24.41	215	275.58	2.E+10	767	2.E+09
B2ULH2	tr|B2ULH2|B2ULH2_AKKM8 OsmC family protein OS=Akkermansia muciniphila (strain ATCC BAA-835 / DSM 22959 / JCM 33894 / BCRC 81048 / CCUG 64013 / CIP 107961 / Muc) OX=349741 GN=Amuc_1538 PE=4 SV=1	Amuc_1538	4	4	49.30	14.99	136	173.93	9.E+09	241	1.E+09
B2UR48	tr|B2UR48|B2UR48_AKKM8 ATP phosphoribosyltransferase OS=Akkermansia muciniphila (strain ATCC BAA-835 / DSM 22959 / JCM 33894 / BCRC 81048 / CCUG 64013 / CIP 107961 / Muc) OX=349741 GN=hisG PE=3 SV=1	Amuc_1107	11	11	52.20	32.70	291	323.31	2.E+10	397	1.E+09
B2UP53	tr|B2UP53|B2UP53_AKKM8 Cysteine synthase OS=Akkermansia muciniphila (strain ATCC BAA-835 / DSM 22959 / JCM 33894 / BCRC 81048 / CCUG 64013 / CIP 107961 / Muc) OX=349741 GN=Amuc_2014 PE=3 SV=1	Amuc_2014	15	15	86.40	32.53	309	323.31	2.E+10	867	1.E+09
B2UPE1	tr|B2UPE1|B2UPE1_AKKM8 Meso-diaminopimelate D-dehydrogenase OS=Akkermansia muciniphila (strain ATCC BAA-835 / DSM 22959 / JCM 33894 / BCRC 81048 / CCUG 64013 / CIP 107961 / Muc) OX=349741 GN=Amuc_0581 PE=3 SV=1	Amuc_0581	17	17	72.40	31.31	293	323.31	2.E+10	1032	1.E+09
B2URE7	sp|B2URE7|PDXJ_AKKM8 Pyridoxine 5-phosphate synthase OS=Akkermansia muciniphila (strain ATCC BAA-835 / DSM 22959 / JCM 33894 / BCRC 81048 / CCUG 64013 / CIP 107961 / Muc) OX=349741 GN=pdxJ PE=3 SV=1	Amuc_1207	7	7	49.60	27.11	246	323.31	1.E+10	1276	1.E+09

B2UL89	tr|B2UL89|B2UL89_AKKM8 NTP_transferase domain-containing protein OS=Akkermansia muciniphila (strain ATCC BAA-835 / DSM 22959 / JCM 33894 / BCRC 81048 / CCUG 64013 / CIP 107961 / Muc) OX=349741 GN=Amuc_0031 PE=4 SV=1	Amuc_0031	10	10	46.70	32.12	291	164.51	2.E+10	381	1.E+09
B2UMF1	sp|B2UMF1|DAPB_AKKM8 4-hydroxy-tetrahydrodipicolinate reductase OS=Akkermansia muciniphila (strain ATCC BAA-835 / DSM 22959 / JCM 33894 / BCRC 81048 / CCUG 64013 / CIP 107961 / Muc) OX=349741 GN=dapB PE=3 SV=1	Amuc_0257	8	8	50.00	26.31	246	289.43	2.E+10	443	1.E+09
B2UMG7	tr|B2UMG7|B2UMG7_AKKM8 Endoribonuclease L-PSP OS=Akkermansia muciniphila (strain ATCC BAA-835 / DSM 22959 / JCM 33894 / BCRC 81048 / CCUG 64013 / CIP 107961 / Muc) OX=349741 GN=Amuc_1705 PE=4 SV=1	Amuc_1705	6	6	67.90	16.64	156	323.31	8.E+09	590	1.E+09
B2UNK1	tr|B2UNK1|B2UNK1_AKKM8 Uncharacterized protein OS=Akkermansia muciniphila (strain ATCC BAA-835 / DSM 22959 / JCM 33894 / BCRC 81048 / CCUG 64013 / CIP 107961 / Muc) OX=349741 GN=Amuc_1903 PE=4 SV=1	Amuc_1903	10	10	40.30	35.41	335	323.31	2.E+10	554	1.E+09
B2UM96	tr|B2UM96|B2UM96_AKKM8 Carboxyl transferase OS=Akkermansia muciniphila (strain ATCC BAA-835 / DSM 22959 / JCM 33894 / BCRC 81048 / CCUG 64013 / CIP 107961 / Muc) OX=349741 GN=Amuc_0201 PE=4 SV=1	Amuc_0201	11	11	33.60	56.01	521	323.31	2.E+10	506	1.E+09
B2ULV5	tr|B2ULV5|B2ULV5_AKKM8 Phosphoglucomutase/phosphomannomutase alpha/beta/alpha domain I OS=Akkermansia muciniphila (strain ATCC BAA-835 / DSM 22959 / JCM 33894 / BCRC 81048 / CCUG 64013 / CIP 107961 / Muc) OX=349741 GN=Amuc_0155 PE=3 SV=1	Amuc_0155	20	20	42.70	71.24	651	323.31	3.E+10	1126	1.E+09
B2UP27	tr|B2UP27|B2UP27_AKKM8 Argininosuccinate synthase OS=Akkermansia muciniphila (strain ATCC BAA-835 / DSM 22959 / JCM 33894 / BCRC 81048 / CCUG 64013 / CIP 107961 / Muc) OX=349741 GN=argG PE=3 SV=1	Amuc_0559	14	14	44.70	47.87	430	308.42	2.E+10	1173	1.E+09
B2UQY9	sp|B2UQY9|EFTU_AKKM8 Elongation factor Tu OS=Akkermansia muciniphila (strain ATCC BAA-835 / DSM 22959 / JCM 33894 / BCRC 81048 / CCUG 64013 / CIP 107961 / Muc) OX=349741 GN=tuf PE=3 SV=1	Amuc_1048	10	10	34.30	43.43	394	323.31	2.E+10	628	1.E+09
B2UMA1	tr|B2UMA1|B2UMA1_AKKM8 Succinate CoA transferase OS=Akkermansia muciniphila (strain ATCC BAA-835 / DSM 22959 / JCM 33894 / BCRC 81048 / CCUG 64013 / CIP 107961 / Muc) OX=349741 GN=Amuc_0206 PE=3 SV=1	Amuc_0206	23	23	61.00	54.05	498	323.31	2.E+10	859	9.E+08
B2UKM8	tr|B2UKM8|B2UKM8_AKKM8 3-oxoacyl-[acyl-carrier-protein] synthase 2 OS=Akkermansia muciniphila (strain ATCC BAA-835 / DSM 22959 / JCM 33894 / BCRC 81048 / CCUG 64013 / CIP 107961 / Muc) OX=349741 GN=Amuc_1327 PE=3 SV=1	Amuc_1327	8	8	34.70	43.82	415	251.36	1.E+10	885	9.E+08
B2UMF2	tr|B2UMF2|B2UMF2_AKKM8 4-hydroxy-tetrahydrodipicolinate synthase OS=Akkermansia muciniphila (strain ATCC BAA-835 / DSM 22959 / JCM 33894 / BCRC 81048 / CCUG 64013 / CIP 107961 / Muc) OX=349741 GN=dapA PE=3 SV=1	Amuc_0258	7	7	34.10	31.25	293	205.55	2.E+10	458	9.E+08
B2UNS9	tr|B2UNS9|B2UNS9_AKKM8 Methylmalonyl-CoA mutase OS=Akkermansia muciniphila (strain ATCC BAA-835 / DSM 22959 / JCM 33894 / BCRC 81048 / CCUG 64013 / CIP 107961 / Muc) OX=349741 GN=Amuc_1984 PE=4 SV=1	Amuc_1984	26	26	50.60	74.10	684	323.31	3.E+10	1054	9.E+08
B2UP95	tr|B2UP95|B2UP95_AKKM8 Rubrerythrin domain-containing protein OS=Akkermansia muciniphila (strain ATCC BAA-835 / DSM 22959 / JCM 33894 / BCRC 81048 / CCUG 64013 / CIP 107961 / Muc) OX=349741 GN=Amuc_2056 PE=4 SV=1	Amuc_2056	3	1	36.60	13.69	123	78.43	6.E+09	123	8.E+08
B2UPB0	tr|B2UPB0|B2UPB0_AKKM8 Rubrerythrin OS=Akkermansia muciniphila (strain ATCC BAA-835 / DSM 22959 / JCM 33894 / BCRC 81048 / CCUG 64013 / CIP 107961 / Muc) OX=349741 GN=Amuc_2072 PE=4 SV=1	Amuc_2072	3	3	19.90	21.34	191	177.17	6.E+09	143	8.E+08
B2UQW7	tr|B2UQW7|B2UQW7_AKKM8 Peptidyl-prolyl cis-trans isomerase OS=Akkermansia muciniphila (strain ATCC BAA-835 / DSM 22959 / JCM 33894 / BCRC 81048 / CCUG 64013 / CIP 107961 / Muc) OX=349741 GN=Amuc_1026 PE=3 SV=1	Amuc_1026	5	5	47.90	17.92	167	260.88	6.E+09	192	8.E+08
B2UQ07	tr|B2UQ07|B2UQ07_AKKM8 2-oxoglutarate reductase OS=Akkermansia muciniphila (strain ATCC BAA-835 / DSM 22959 / JCM 33894 / BCRC 81048 / CCUG 64013 / CIP 107961 / Muc) OX=349741 GN=Amuc_0705 PE=3 SV=1	Amuc_0705	14	14	37.90	56.68	523	323.31	2.E+10	561	8.E+08
B2UMX5	tr|B2UMX5|B2UMX5_AKKM8 OsmC family protein OS=Akkermansia muciniphila (strain ATCC BAA-835 / DSM 22959 / JCM 33894 / BCRC 81048 / CCUG 64013 / CIP 107961 / Muc) OX=349741 GN=Amuc_0342 PE=4 SV=1	Amuc_0342	7	7	65.20	14.48	135	113.32	5.E+09	262	8.E+08

B2UNP8	tr|B2UNP8|B2UNP8_AKKM8 NADH:flavin oxidoreductase/NADH oxidase OS=Akkermansia muciniphila (strain ATCC BAA-835 / DSM 22959 / JCM 33894 / BCRC 81048 / CCUG 64013 / CIP 107961 / Muc) OX=349741 GN=Amuc_1951 PE=4 SV=1	Amuc_1951	16	16	49.90	40.82	367	323.31	1.E+10	578	7.E+08
B2UPU6	tr|B2UPU6|B2UPU6_AKKM8 Oligopeptidase A OS=Akkermansia muciniphila (strain ATCC BAA-835 / DSM 22959 / JCM 33894 / BCRC 81048 / CCUG 64013 / CIP 107961 / Muc) OX=349741 GN=Amuc_2167 PE=3 SV=1	Amuc_2167	18	18	38.40	79.32	700	323.31	3.E+10	871	7.E+08
B2URP8	tr|B2URP8|B2URP8_AKKM8 Aldose 1-epimerase OS=Akkermansia muciniphila (strain ATCC BAA-835 / DSM 22959 / JCM 33894 / BCRC 81048 / CCUG 64013 / CIP 107961 / Muc) OX=349741 GN=Amuc_1309 PE=3 SV=1	Amuc_1309	11	11	49.20	34.50	319	287.90	1.E+10	405	7.E+08
B2UL16	tr|B2UL16|B2UL16_AKKM8 Uncharacterized protein OS=Akkermansia muciniphila (strain ATCC BAA-835 / DSM 22959 / JCM 33894 / BCRC 81048 / CCUG 64013 / CIP 107961 / Muc) OX=349741 GN=Amuc_1467 PE=4 SV=1	Amuc_1467	4	4	16.50	22.00	194	98.66	6.E+09	160	7.E+08
B2UN39	tr|B2UN39|B2UN39_AKKM8 L-fucose isomerase OS=Akkermansia muciniphila (strain ATCC BAA-835 / DSM 22959 / JCM 33894 / BCRC 81048 / CCUG 64013 / CIP 107961 / Muc) OX=349741 GN=fucI PE=3 SV=1	Amuc_1832	20	20	39.20	64.70	592	323.31	2.E+10	765	7.E+08
B2UML6	sp|B2UML6|OTC_AKKM8 Ornithine carbamoyltransferase OS=Akkermansia muciniphila (strain ATCC BAA-835 / DSM 22959 / JCM 33894 / BCRC 81048 / CCUG 64013 / CIP 107961 / Muc) OX=349741 GN=arcB PE=3 SV=1	Amuc_1630	11	11	47.90	34.14	305	323.31	1.E+10	527	7.E+08
B2UMU5	sp|B2UMU5|GPMI_AKKM8 2,3-bisphosphoglycerate-independent phosphoglycerate mutase OS=Akkermansia muciniphila (strain ATCC BAA-835 / DSM 22959 / JCM 33894 / BCRC 81048 / CCUG 64013 / CIP 107961 / Muc) OX=349741 GN=gpmI PE=3 SV=1	Amuc_0309	14	14	37.70	56.14	514	323.31	2.E+10	554	7.E+08
B2UPJ1	tr|B2UPJ1|B2UPJ1_AKKM8 FAD-binding domain protein OS=Akkermansia muciniphila (strain ATCC BAA-835 / DSM 22959 / JCM 33894 / BCRC 81048 / CCUG 64013 / CIP 107961 / Muc) OX=349741 GN=Amuc_0631 PE=4 SV=1	Amuc_0631	13	13	53.50	41.44	376	323.31	1.E+10	521	7.E+08
B2UNK9	tr|B2UNK9|B2UNK9_AKKM8 Iron-containing alcohol dehydrogenase OS=Akkermansia muciniphila (strain ATCC BAA-835 / DSM 22959 / JCM 33894 / BCRC 81048 / CCUG 64013 / CIP 107961 / Muc) OX=349741 GN=Amuc_1911 PE=4 SV=1	Amuc_1911	9	9	37.60	41.35	386	175.54	9.E+09	482	7.E+08
B2UMC9	tr|B2UMC9|B2UMC9_AKKM8 Alpha-glucan phosphorylase OS=Akkermansia muciniphila (strain ATCC BAA-835 / DSM 22959 / JCM 33894 / BCRC 81048 / CCUG 64013 / CIP 107961 / Muc) OX=349741 GN=Amuc_0235 PE=3 SV=1	Amuc_0235	22	22	49.50	63.41	554	323.31	2.E+10	936	6.E+08
B2UPE0	tr|B2UPE0|B2UPE0_AKKM8 Entericidin EcnAB OS=Akkermansia muciniphila (strain ATCC BAA-835 / DSM 22959 / JCM 33894 / BCRC 81048 / CCUG 64013 / CIP 107961 / Muc) OX=349741 GN=Amuc_0580 PE=4 SV=1	Amuc_0580	1	1	29.80	4.74	47	40.49	1.E+09	24	6.E+08
B2UPQ6	tr|B2UPQ6|B2UPQ6_AKKM8 Adenylosuccinate synthetase OS=Akkermansia muciniphila (strain ATCC BAA-835 / DSM 22959 / JCM 33894 / BCRC 81048 / CCUG 64013 / CIP 107961 / Muc) OX=349741 GN=purA PE=3 SV=1	Amuc_2125	10	10	28.80	46.05	423	219.74	1.E+10	788	6.E+08
B2UL87	tr|B2UL87|B2UL87_AKKM8 NAD-dependent epimerase/dehydratase OS=Akkermansia muciniphila (strain ATCC BAA-835 / DSM 22959 / JCM 33894 / BCRC 81048 / CCUG 64013 / CIP 107961 / Muc) OX=349741 GN=Amuc_0029 PE=4 SV=1	Amuc_0029	10	10	39.30	33.59	308	165.07	1.E+10	440	6.E+08
B2UP16	tr|B2UP16|B2UP16_AKKM8 MotA/TolQ/ExbB proton channel OS=Akkermansia muciniphila (strain ATCC BAA-835 / DSM 22959 / JCM 33894 / BCRC 81048 / CCUG 64013 / CIP 107961 / Muc) OX=349741 GN=Amuc_0548 PE=3 SV=1	Amuc_0548	2	2	11.80	28.34	263	12.58	3.E+09	44	6.E+08
B2UNS1	tr|B2UNS1|B2UNS1_AKKM8 Pyruvate, phosphate dikinase OS=Akkermansia muciniphila (strain ATCC BAA-835 / DSM 22959 / JCM 33894 / BCRC 81048 / CCUG 64013 / CIP 107961 / Muc) OX=349741 GN=Amuc_1974 PE=3 SV=1	Amuc_1974	22	22	30.60	100.12	914	323.31	3.E+10	1239	6.E+08
B2UKY9	tr|B2UKY9|B2UKY9_AKKM8 Nitroreductase OS=Akkermansia muciniphila (strain ATCC BAA-835 / DSM 22959 / JCM 33894 / BCRC 81048 / CCUG 64013 / CIP 107961 / Muc) OX=349741 GN=Amuc_1440 PE=3 SV=1	Amuc_1440	7	7	31.60	24.93	215	58.67	5.E+09	169	6.E+08
B2UQR6	tr|B2UQR6|B2UQR6_AKKM8 Galactokinase OS=Akkermansia muciniphila (strain ATCC BAA-835 / DSM 22959 / JCM 33894 / BCRC 81048 / CCUG 64013 / CIP 107961 / Muc) OX=349741 GN=Amuc_0969 PE=3 SV=1	Amuc_0969	9	9	29.30	42.15	392	223.84	1.E+10	418	5.E+08

B2UN63	tr|B2UN63|B2UN63_AKKM8 Formate--tetrahydrofolate ligase OS=Akkermansia muciniphila (strain ATCC BAA-835 / DSM 22959 / JCM 33894 / BCRC 81048 / CCUG 64013 / CIP 107961 / Muc) OX=349741 GN=fhs PE=3 SV=1	Amuc_1860	17	17	40.90	59.80	557	323.31	2.E+10	898	5.E+08
B2UMC7	tr|B2UMC7|B2UMC7_AKKM8 L-threonine 3-dehydrogenase OS=Akkermansia muciniphila (strain ATCC BAA-835 / DSM 22959 / JCM 33894 / BCRC 81048 / CCUG 64013 / CIP 107961 / Muc) OX=349741 GN=tdh PE=3 SV=1	Amuc_0233	7	7	31.00	37.54	345	250.40	7.E+09	294	5.E+08
B2UM06	tr|B2UM06|B2UM06_AKKM8 Serine hydroxymethyltransferase OS=Akkermansia muciniphila (strain ATCC BAA-835 / DSM 22959 / JCM 33894 / BCRC 81048 / CCUG 64013 / CIP 107961 / Muc) OX=349741 GN=glyA PE=3 SV=1	Amuc_1630	22	22	50.40	61.10	566	323.31	2.E+10	849	5.E+08
B2UP04	tr|B2UP04|B2UP04_AKKM8 Uncharacterized protein OS=Akkermansia muciniphila (strain ATCC BAA-835 / DSM 22959 / JCM 33894 / BCRC 81048 / CCUG 64013 / CIP 107961 / Muc) OX=349741 GN=Amuc_0536 PE=4 SV=1	Amuc_0536	6	6	34.00	28.70	259	195.84	6.E+09	190	5.E+08
B2UMA5	tr|B2UMA5|B2UMA5_AKKM8 6-phosphofructokinase OS=Akkermansia muciniphila (strain ATCC BAA-835 / DSM 22959 / JCM 33894 / BCRC 81048 / CCUG 64013 / CIP 107961 / Muc) OX=349741 GN=Amuc_0210 PE=4 SV=1	Amuc_0210	7	7	25.00	35.31	320	144.82	6.E+09	251	5.E+08
B2UQW6	tr|B2UQW6|B2UQW6_AKKM8 Disulfide bond chaperones of the HSP33 family-like protein OS=Akkermansia muciniphila (strain ATCC BAA-835 / DSM 22959 / JCM 33894 / BCRC 81048 / CCUG 64013 / CIP 107961 / Muc) OX=349741 GN=Amuc_1025 PE=4 SV=1	Amuc_1025	10	10	47.10	30.09	257	310.77	7.E+09	575	5.E+08
B2UNP3	tr|B2UNP3|B2UNP3_AKKM8 Dihydrodipicolinate synthetase OS=Akkermansia muciniphila (strain ATCC BAA-835 / DSM 22959 / JCM 33894 / BCRC 81048 / CCUG 64013 / CIP 107961 / Muc) OX=349741 GN=Amuc_1946 PE=3 SV=1	Amuc_1946	6	6	25.60	33.03	305	119.27	8.E+09	463	5.E+08
B2UPS4	tr|B2UPS4|B2UPS4_AKKM8 Fumarate hydratase class I OS=Akkermansia muciniphila (strain ATCC BAA-835 / DSM 22959 / JCM 33894 / BCRC 81048 / CCUG 64013 / CIP 107961 / Muc) OX=349741 GN=Amuc_2144 PE=3 SV=1	Amuc_2144	21	21	53.80	60.10	548	323.31	2.E+10	611	5.E+08
B2UN19	tr|B2UN19|B2UN19_AKKM8 Alpha amylase catalytic region OS=Akkermansia muciniphila (strain ATCC BAA-835 / DSM 22959 / JCM 33894 / BCRC 81048 / CCUG 64013 / CIP 107961 / Muc) OX=349741 GN=Amuc_1812 PE=3 SV=1	Amuc_1812	12	12	37.00	56.72	492	162.51	1.E+10	509	4.E+08
B2UQK1	tr|B2UQK1|B2UQK1_AKKM8 Aconitate hydratase OS=Akkermansia muciniphila (strain ATCC BAA-835 / DSM 22959 / JCM 33894 / BCRC 81048 / CCUG 64013 / CIP 107961 / Muc) OX=349741 GN=Amuc_0904 PE=3 SV=1	Amuc_0904	23	23	38.40	99.01	917	323.31	2.E+10	1077	4.E+08
B2URI9	tr|B2URI9|B2URI9_AKKM8 GDP-mannose 4,6-dehydratase OS=Akkermansia muciniphila (strain ATCC BAA-835 / DSM 22959 / JCM 33894 / BCRC 81048 / CCUG 64013 / CIP 107961 / Muc) OX=349741 GN=gmd PE=3 SV=1	Amuc_1249	14	14	51.00	40.86	355	219.04	9.E+09	591	4.E+08
B2UQE5	tr|B2UQE5|B2UQE5_AKKM8 Metal dependent phosphohydrolase OS=Akkermansia muciniphila (strain ATCC BAA-835 / DSM 22959 / JCM 33894 / BCRC 81048 / CCUG 64013 / CIP 107961 / Muc) OX=349741 GN=Amuc_0847 PE=4 SV=1	Amuc_0847	9	9	33.90	39.25	354	58.34	7.E+09	76	4.E+08
B2UMK3	tr|B2UMK3|B2UMK3_AKKM8 UTP--glucose-1-phosphate uridylyltransferase OS=Akkermansia muciniphila (strain ATCC BAA-835 / DSM 22959 / JCM 33894 / BCRC 81048 / CCUG 64013 / CIP 107961 / Muc) OX=349741 GN=Amuc_1741 PE=3 SV=1	Amuc_1741	15	15	42.50	50.44	461	291.62	9.E+09	394	4.E+08
B2UN83	tr|B2UN83|B2UN83_AKKM8 Isocitrate dehydrogenase [NADP] OS=Akkermansia muciniphila (strain ATCC BAA-835 / DSM 22959 / JCM 33894 / BCRC 81048 / CCUG 64013 / CIP 107961 / Muc) OX=349741 GN=Amuc_1880 PE=4 SV=1	Amuc_1880	18	18	50.10	45.31	415	166.12	9.E+09	552	4.E+08
B2UQ06	tr|B2UQ06|B2UQ06_AKKM8 Uncharacterized protein OS=Akkermansia muciniphila (strain ATCC BAA-835 / DSM 22959 / JCM 33894 / BCRC 81048 / CCUG 64013 / CIP 107961 / Muc) OX=349741 GN=Amuc_0704 PE=4 SV=1	Amuc_0704	9	9	26.40	47.48	417	313.47	8.E+09	349	4.E+08
B2UMM1	tr|B2UMM1|B2UMM1_AKKM8 Peptidase M20 OS=Akkermansia muciniphila (strain ATCC BAA-835 / DSM 22959 / JCM 33894 / BCRC 81048 / CCUG 64013 / CIP 107961 / Muc) OX=349741 GN=Amuc_1759 PE=4 SV=1	Amuc_1759	11	11	24.30	50.33	465	139.33	7.E+09	496	3.E+08
B2UR66	tr|B2UR66|B2UR66_AKKM8 UDP-glucose 4-epimerase OS=Akkermansia muciniphila (strain ATCC BAA-835 / DSM 22959 / JCM 33894 / BCRC 81048 / CCUG 64013 / CIP 107961 / Muc) OX=349741 GN=Amuc_1125 PE=3 SV=1	Amuc_1125	7	7	31.30	36.07	329	100.94	5.E+09	310	3.E+08

B2UKM2	tr|B2UKM2|B2UKM2_AKKM8 Alkyl hydroperoxide reductase/ Thiol specific antioxidant/ Mal allergen OS=Akkermansia muciniphila (strain ATCC BAA-835 / DSM 22959 / JCM 33894 / BCRC 81048 / CCUG 64013 / CIP 107961 / Muc) OX=349741 GN=Amuc_1321 PE=3 SV=1	Amuc_1321	5	5	36.20	22.83	207	126.45	3.E+09	170	3.E+08
B2UN36	tr|B2UN36|B2UN36_AKKM8 Class II aldolase/adducin family protein OS=Akkermansia muciniphila (strain ATCC BAA-835 / DSM 22959 / JCM 33894 / BCRC 81048 / CCUG 64013 / CIP 107961 / Muc) OX=349741 GN=Amuc_1829 PE=4 SV=1	Amuc_1829	7	7	39.90	29.81	271	200.78	4.E+09	313	3.E+08
B2UKP3	tr|B2UKP3|B2UKP3_AKKM8 Uncharacterized protein OS=Akkermansia muciniphila (strain ATCC BAA-835 / DSM 22959 / JCM 33894 / BCRC 81048 / CCUG 64013 / CIP 107961 / Muc) OX=349741 GN=Amuc_1342 PE=4 SV=1	Amuc_1342	4	4	17.30	34.98	312	90.38	5.E+09	235	3.E+08
B2UMG1	tr|B2UMG1|B2UMG1_AKKM8 Uncharacterized protein OS=Akkermansia muciniphila (strain ATCC BAA-835 / DSM 22959 / JCM 33894 / BCRC 81048 / CCUG 64013 / CIP 107961 / Muc) OX=349741 GN=Amuc_0267 PE=4 SV=1	Amuc_0267	5	5	30.10	18.11	163	118.25	3.E+09	255	3.E+08
B2UR47	tr|B2UR47|B2UR47_AKKM8 Peptidase M24 OS=Akkermansia muciniphila (strain ATCC BAA-835 / DSM 22959 / JCM 33894 / BCRC 81048 / CCUG 64013 / CIP 107961 / Muc) OX=349741 GN=Amuc_1106 PE=4 SV=1	Amuc_1106	11	11	26.00	48.19	427	134.55	6.E+09	465	3.E+08
B2ULA2	tr|B2ULA2|B2ULA2_AKKM8 Diaminopimelate epimerase OS=Akkermansia muciniphila (strain ATCC BAA-835 / DSM 22959 / JCM 33894 / BCRC 81048 / CCUG 64013 / CIP 107961 / Muc) OX=349741 GN=dapF PE=3 SV=1	Amuc_0044	6	6	28.50	29.30	274	140.20	4.E+09	193	3.E+08
B2UL47	tr|B2UL47|B2UL47_AKKM8 Uncharacterized protein OS=Akkermansia muciniphila (strain ATCC BAA-835 / DSM 22959 / JCM 33894 / BCRC 81048 / CCUG 64013 / CIP 107961 / Muc) OX=349741 GN=Amuc_1498 PE=3 SV=1	Amuc_1498	8	8	43.20	25.20	229	106.89	3.E+09	238	3.E+08
B2UPF7	tr|B2UPF7|B2UPF7_AKKM8 Flavin_Reduct domain-containing protein OS=Akkermansia muciniphila (strain ATCC BAA-835 / DSM 22959 / JCM 33894 / BCRC 81048 / CCUG 64013 / CIP 107961 / Muc) OX=349741 GN=Amuc_0597 PE=4 SV=1	Amuc_0597	4	4	30.40	19.05	168	60.60	2.E+09	220	3.E+08
B2UQT1	tr|B2UQT1|B2UQT1_AKKM8 Uncharacterized protein OS=Akkermansia muciniphila (strain ATCC BAA-835 / DSM 22959 / JCM 33894 / BCRC 81048 / CCUG 64013 / CIP 107961 / Muc) OX=349741 GN=Amuc_0987 PE=4 SV=1	Amuc_0987	5	5	67.80	9.56	90	65.07	2.E+09	167	3.E+08
B2URF8	tr|B2URF8|B2URF8_AKKM8 3-dehydroquinate synthase OS=Akkermansia muciniphila (strain ATCC BAA-835 / DSM 22959 / JCM 33894 / BCRC 81048 / CCUG 64013 / CIP 107961 / Muc) OX=349741 GN=aroB PE=3 SV=1	Amuc_1218	11	11	44.10	39.26	370	178.11	6.E+09	214	3.E+08
B2UR44	tr|B2UR44|B2UR44_AKKM8 Glutamyl aminopeptidase OS=Akkermansia muciniphila (strain ATCC BAA-835 / DSM 22959 / JCM 33894 / BCRC 81048 / CCUG 64013 / CIP 107961 / Muc) OX=349741 GN=Amuc_1103 PE=3 SV=1	Amuc_1103	8	8	29.50	38.13	359	70.29	5.E+09	169	3.E+08
B2UQ25	tr|B2UQ25|B2UQ25_AKKM8 Branched-chain-amino-acid aminotransferase OS=Akkermansia muciniphila (strain ATCC BAA-835 / DSM 22959 / JCM 33894 / BCRC 81048 / CCUG 64013 / CIP 107961 / Muc) OX=349741 GN=ilvE PE=3 SV=1	Amuc_0723	6	6	31.90	31.84	288	120.56	5.E+09	154	3.E+08
B2UKZ6	sp|B2UKZ6|ARGC_AKKM8 N-acetyl-gamma-glutamyl-phosphate reductase OS=Akkermansia muciniphila (strain ATCC BAA-835 / DSM 22959 / JCM 33894 / BCRC 81048 / CCUG 64013 / CIP 107961 / Muc) OX=349741 GN=argC PE=3 SV=1	Amuc_1447	7	7	34.60	37.59	347	137.05	4.E+09	246	3.E+08
B2UPN7	tr|B2UPN7|B2UPN7_AKKM8 Malate dehydrogenase (Oxaloacetate-decarboxylating) (NADP(+)) OS=Akkermansia muciniphila (strain ATCC BAA-835 / DSM 22959 / JCM 33894 / BCRC 81048 / CCUG 64013 / CIP 107961 / Muc) OX=349741 GN=Amuc_2106 PE=3 SV=1	Amuc_2106	12	12	45.00	47.52	436	166.38	6.E+09	271	3.E+08
B2UMD1	tr|B2UMD1|B2UMD1_AKKM8 Arsenate reductase and related OS=Akkermansia muciniphila (strain ATCC BAA-835 / DSM 22959 / JCM 33894 / BCRC 81048 / CCUG 64013 / CIP 107961 / Muc) OX=349741 GN=Amuc_0237 PE=3 SV=1	Amuc_0237	5	5	46.20	13.52	119	55.32	2.E+09	110	3.E+08
B2URB8	sp|B2URB8|ILVC_AKKM8 Ketol-acid reductoisomerase (NADP(+)) OS=Akkermansia muciniphila (strain ATCC BAA-835 / DSM 22959 / JCM 33894 / BCRC 81048 / CCUG 64013 / CIP 107961 / Muc) OX=349741 GN=ilvC PE=3 SV=1	Amuc_1178	10	10	44.80	35.14	326	175.89	4.E+09	297	3.E+08
B2UMZ5	tr|B2UMZ5|B2UMZ5_AKKM8 Uncharacterized protein OS=Akkermansia muciniphila (strain ATCC BAA-835 / DSM 22959 / JCM 33894 / BCRC 81048 / CCUG 64013 / CIP 107961 / Muc) OX=349741 GN=Amuc_0362 PE=4 SV=1	Amuc_0362	2	2	19.30	15.36	140	49.49	2.E+09	70	2.E+08

B2UMM9	tr|B2UMM9|B2UMM9_AKKM8 Uncharacterized protein OS=Akkermansia muciniphila (strain ATCC BAA-835 / DSM 22959 / JCM 33894 / BCRC 81048 / CCUG 64013 / CIP 107961 / Muc) OX=349741 GN=Amuc_1767 PE=4 SV=1	Amuc_1767	11	11	47.60	32.22	296	317.01	4.E+09	236	2.E+08
B2UP96	tr|B2UP96|B2UP96_AKKM8 Thioredoxin domain-containing protein OS=Akkermansia muciniphila (strain ATCC BAA-835 / DSM 22959 / JCM 33894 / BCRC 81048 / CCUG 64013 / CIP 107961 / Muc) OX=349741 GN=Amuc_2057 PE=4 SV=1	Amuc_2057	4	4	29.30	18.12	167	48.08	2.E+09	144	2.E+08
B2ULL3	tr|B2ULL3|B2ULL3_AKKM8 Nucleoside-diphosphate kinase OS=Akkermansia muciniphila (strain ATCC BAA-835 / DSM 22959 / JCM 33894 / BCRC 81048 / CCUG 64013 / CIP 107961 / Muc) OX=349741 GN=Amuc_1580 PE=3 SV=1	Amuc_1580	6	6	41.70	16.91	151	72.83	3.E+09	130	2.E+08
B2ULU7	tr|B2ULU7|B2ULU7_AKKM8 Thioredoxin domain OS=Akkermansia muciniphila (strain ATCC BAA-835 / DSM 22959 / JCM 33894 / BCRC 81048 / CCUG 64013 / CIP 107961 / Muc) OX=349741 GN=Amuc_0147 PE=4 SV=1	Amuc_0147	9	9	29.60	34.97	321	86.17	4.E+09	274	2.E+08
B2UP90	tr|B2UP90|B2UP90_AKKM8 Glutamate dehydrogenase OS=Akkermansia muciniphila (strain ATCC BAA-835 / DSM 22959 / JCM 33894 / BCRC 81048 / CCUG 64013 / CIP 107961 / Muc) OX=349741 GN=Amuc_2051 PE=3 SV=1	Amuc_2051	14	14	41.70	49.57	451	226.25	7.E+09	417	2.E+08
B2UQH2	tr|B2UQH2|B2UQH2_AKKM8 Elongation factor P OS=Akkermansia muciniphila (strain ATCC BAA-835 / DSM 22959 / JCM 33894 / BCRC 81048 / CCUG 64013 / CIP 107961 / Muc) OX=349741 GN=efp PE=3 SV=1	Amuc_0874	4	4	23.40	20.91	188	93.81	2.E+09	50	2.E+08
B2UPN4	tr|B2UPN4|B2UPN4_AKKM8 Methionine--tRNA ligase OS=Akkermansia muciniphila (strain ATCC BAA-835 / DSM 22959 / JCM 33894 / BCRC 81048 / CCUG 64013 / CIP 107961 / Muc) OX=349741 GN=Amuc_2103 PE=3 SV=1	Amuc_2103	9	9	19.30	62.13	550	235.75	5.E+09	320	2.E+08
B2UPY9	tr|B2UPY9|B2UPY9_AKKM8 Outer membrane autotransporter barrel domain protein OS=Akkermansia muciniphila (strain ATCC BAA-835 / DSM 22959 / JCM 33894 / BCRC 81048 / CCUG 64013 / CIP 107961 / Muc) OX=349741 GN=Amuc_0687 PE=4 SV=1	Amuc_0687	13	13	27.30	107.54	1044	323.31	8.E+09	284	2.E+08
B2UQ32	tr|B2UQ32|B2UQ32_AKKM8 ATP-grasp domain-containing protein OS=Akkermansia muciniphila (strain ATCC BAA-835 / DSM 22959 / JCM 33894 / BCRC 81048 / CCUG 64013 / CIP 107961 / Muc) OX=349741 GN=Amuc_0730 PE=4 SV=1	Amuc_0730	9	9	33.70	38.30	341	127.29	4.E+09	366	2.E+08
B2UQB4	tr|B2UQB4|B2UQB4_AKKM8 Beta sliding clamp OS=Akkermansia muciniphila (strain ATCC BAA-835 / DSM 22959 / JCM 33894 / BCRC 81048 / CCUG 64013 / CIP 107961 / Muc) OX=349741 GN=Amuc_0816 PE=3 SV=1	Amuc_0816	11	11	42.30	40.54	366	168.71	5.E+09	246	2.E+08
B2UQ98	tr|B2UQ98|B2UQ98_AKKM8 Pyruvate flavodoxin/ferredoxin oxidoreductase domain protein OS=Akkermansia muciniphila (strain ATCC BAA-835 / DSM 22959 / JCM 33894 / BCRC 81048 / CCUG 64013 / CIP 107961 / Muc) OX=349741 GN=Amuc_0799 PE=3 SV=1	Amuc_0799	22	22	27.50	129.66	1192	323.31	1.E+10	737	2.E+08
B2ULC0	tr|B2ULC0|B2ULC0_AKKM8 5-oxopent-3-ene-1,2,5-tricarboxylate decarboxylase OS=Akkermansia muciniphila (strain ATCC BAA-835 / DSM 22959 / JCM 33894 / BCRC 81048 / CCUG 64013 / CIP 107961 / Muc) OX=349741 GN=Amuc_0064 PE=4 SV=1	Amuc_0064	7	7	36.00	31.33	292	130.07	3.E+09	226	2.E+08
B2UN16	tr|B2UN16|B2UN16_AKKM8 NAD(P)H dehydrogenase (Quinone) OS=Akkermansia muciniphila (strain ATCC BAA-835 / DSM 22959 / JCM 33894 / BCRC 81048 / CCUG 64013 / CIP 107961 / Muc) OX=349741 GN=Amuc_1809 PE=4 SV=1	Amuc_1809	5	5	26.50	20.95	185	48.54	2.E+09	122	2.E+08
B2UQ84	tr|B2UQ84|B2UQ84_AKKM8 3-isopropylmalate dehydrogenase OS=Akkermansia muciniphila (strain ATCC BAA-835 / DSM 22959 / JCM 33894 / BCRC 81048 / CCUG 64013 / CIP 107961 / Muc) OX=349741 GN=leuB PE=3 SV=1	Amuc_0785	10	10	29.00	39.80	369	148.93	4.E+09	271	2.E+08
B2UNN8	tr|B2UNN8|B2UNN8_AKKM8 Adenylosuccinate lyase OS=Akkermansia muciniphila (strain ATCC BAA-835 / DSM 22959 / JCM 33894 / BCRC 81048 / CCUG 64013 / CIP 107961 / Muc) OX=349741 GN=Amuc_1941 PE=3 SV=1	Amuc_1941	12	12	33.50	52.42	474	272.31	5.E+09	378	2.E+08
B2UNE7	tr|B2UNE7|B2UNE7_AKKM8 Glutamine--tRNA ligase OS=Akkermansia muciniphila (strain ATCC BAA-835 / DSM 22959 / JCM 33894 / BCRC 81048 / CCUG 64013 / CIP 107961 / Muc) OX=349741 GN=glnS PE=3 SV=1	Amuc_0421	13	13	32.20	64.11	559	251.66	6.E+09	305	2.E+08
B2UMG2	tr|B2UMG2|B2UMG2_AKKM8 Histidine triad (HIT) protein OS=Akkermansia muciniphila (strain ATCC BAA-835 / DSM 22959 / JCM 33894 / BCRC 81048 / CCUG 64013 / CIP 107961 / Muc) OX=349741 GN=Amuc_0268 PE=4 SV=1	Amuc_0268	2	2	21.90	12.49	114	22.34	1.E+09	82	2.E+08

B2UM17	sp|B2UM17|FOLD_AKKM8 Bifunctional protein FolD OS=Akkermansia muciniphila (strain ATCC BAA-835 / DSM 22959 / JCM 33894 / BCRC 81048 / CCUG 64013 / CIP 107961 / Muc) OX=349741 GN=folD PE=3 SV=1	Amuc_1641	10	10	51.20	30.85	291	136.95	3.E+09	230	2.E+08
B2UPP6	sp|B2UPP6|SYS_AKKM8 Serine--tRNA ligase OS=Akkermansia muciniphila (strain ATCC BAA-835 / DSM 22959 / JCM 33894 / BCRC 81048 / CCUG 64013 / CIP 107961 / Muc) OX=349741 GN=serS PE=3 SV=1	Amuc_2115	7	7	21.60	47.90	426	128.81	3.E+09	208	2.E+08
B2UPN2	tr|B2UPN2|B2UPN2_AKKM8 D-ribose pyranase OS=Akkermansia muciniphila (strain ATCC BAA-835 / DSM 22959 / JCM 33894 / BCRC 81048 / CCUG 64013 / CIP 107961 / Muc) OX=349741 GN=Amuc_2101 PE=4 SV=1	Amuc_2101	4	4	36.70	15.08	139	39.18	8.E+08	69	2.E+08
B2UQ02	tr|B2UQ02|B2UQ02_AKKM8 Uncharacterized protein OS=Akkermansia muciniphila (strain ATCC BAA-835 / DSM 22959 / JCM 33894 / BCRC 81048 / CCUG 64013 / CIP 107961 / Muc) OX=349741 GN=Amuc_0700 PE=4 SV=1	Amuc_0700	2	2	13.70	15.12	139	51.76	5.E+08	26	2.E+08
B2UNE0	sp|B2UNE0|GMHA_AKKM8 Phosphoheptose isomerase OS=Akkermansia muciniphila (strain ATCC BAA-835 / DSM 22959 / JCM 33894 / BCRC 81048 / CCUG 64013 / CIP 107961 / Muc) OX=349741 GN=gmhA PE=3 SV=1	Amuc_0414	3	3	20.60	20.11	189	73.52	1.E+09	132	2.E+08
B2UQT2	tr|B2UQT2|B2UQT2_AKKM8 TatD-related deoxyribonuclease OS=Akkermansia muciniphila (strain ATCC BAA-835 / DSM 22959 / JCM 33894 / BCRC 81048 / CCUG 64013 / CIP 107961 / Muc) OX=349741 GN=Amuc_0988 PE=4 SV=1	Amuc_0988	6	6	32.50	30.79	271	102.11	3.E+09	192	2.E+08
B2UMM5	tr|B2UMM5|B2UMM5_AKKM8 Pyridoxal phosphate homeostasis protein OS=Akkermansia muciniphila (strain ATCC BAA-835 / DSM 22959 / JCM 33894 / BCRC 81048 / CCUG 64013 / CIP 107961 / Muc) OX=349741 GN=Amuc_1763 PE=3 SV=1	Amuc_1763	5	5	27.70	25.63	231	63.66	2.E+09	246	1.E+08
B2UN34	tr|B2UN34|B2UN34_AKKM8 Pirin domain protein OS=Akkermansia muciniphila (strain ATCC BAA-835 / DSM 22959 / JCM 33894 / BCRC 81048 / CCUG 64013 / CIP 107961 / Muc) OX=349741 GN=Amuc_1827 PE=3 SV=1	Amuc_1827	4	4	12.10	26.34	232	15.68	2.E+09	105	1.E+08
B2UNI4	tr|B2UNI4|B2UNI4_AKKM8 Glutamine amidotransferase class-II OS=Akkermansia muciniphila (strain ATCC BAA-835 / DSM 22959 / JCM 33894 / BCRC 81048 / CCUG 64013 / CIP 107961 / Muc) OX=349741 GN=Amuc_0458 PE=4 SV=1	Amuc_0458	18	18	33.30	72.22	639	236.68	4.E+09	410	1.E+08
B2UM77	tr|B2UM77|B2UM77_AKKM8 Ferritin OS=Akkermansia muciniphila (strain ATCC BAA-835 / DSM 22959 / JCM 33894 / BCRC 81048 / CCUG 64013 / CIP 107961 / Muc) OX=349741 GN=Amuc_1703 PE=3 SV=1	Amuc_1703	3	3	17.10	18.97	164	16.60	1.E+09	84	1.E+08
B2ULR5	tr|B2ULR5|B2ULR5_AKKM8 N5-carboxyaminoimidazole ribonucleotide mutase OS=Akkermansia muciniphila (strain ATCC BAA-835 / DSM 22959 / JCM 33894 / BCRC 81048 / CCUG 64013 / CIP 107961 / Muc) OX=349741 GN=purE PE=3 SV=1	Amuc_0114	4	4	40.40	14.14	136	30.20	8.E+08	69	1.E+08
B2ULP8	tr|B2ULP8|B2ULP8_AKKM8 ROK family protein OS=Akkermansia muciniphila (strain ATCC BAA-835 / DSM 22959 / JCM 33894 / BCRC 81048 / CCUG 64013 / CIP 107961 / Muc) OX=349741 GN=Amuc_0097 PE=4 SV=1	Amuc_0097	5	5	22.60	33.91	319	76.81	2.E+09	164	1.E+08
B2UME9	tr|B2UME9|B2UME9_AKKM8 3-methyl-2-oxobutanoate hydroxymethyltransferase OS=Akkermansia muciniphila (strain ATCC BAA-835 / DSM 22959 / JCM 33894 / BCRC 81048 / CCUG 64013 / CIP 107961 / Muc) OX=349741 GN=panB PE=3 SV=1	Amuc_0255	6	6	30.50	27.64	262	65.83	1.E+09	180	1.E+08
B2UR09	sp|B2UR09|METK_AKKM8 S-adenosylmethionine synthase OS=Akkermansia muciniphila (strain ATCC BAA-835 / DSM 22959 / JCM 33894 / BCRC 81048 / CCUG 64013 / CIP 107961 / Muc) OX=349741 GN=metK PE=3 SV=1	Amuc_1068	9	9	29.20	43.52	394	58.07	3.E+09	145	1.E+08
B2UQ68	tr|B2UQ68|B2UQ68_AKKM8 Ferripyochelin binding protein (Fbp) OS=Akkermansia muciniphila (strain ATCC BAA-835 / DSM 22959 / JCM 33894 / BCRC 81048 / CCUG 64013 / CIP 107961 / Muc) OX=349741 GN=Amuc_0768 PE=4 SV=1	Amuc_0768	2	2	14.00	19.07	178	8.97	5.E+08	108	1.E+08
B2UPW8	sp|B2UPW8|LEUC_AKKM8 3-isopropylmalate dehydratase large subunit OS=Akkermansia muciniphila (strain ATCC BAA-835 / DSM 22959 / JCM 33894 / BCRC 81048 / CCUG 64013 / CIP 107961 / Muc) OX=349741 GN=leuC PE=3 SV=1	Amuc_0666	9	9	27.00	50.53	470	94.34	3.E+09	166	1.E+08
B2UPA8	tr|B2UPA8|B2UPA8_AKKM8 Catalase OS=Akkermansia muciniphila (strain ATCC BAA-835 / DSM 22959 / JCM 33894 / BCRC 81048 / CCUG 64013 / CIP 107961 / Muc) OX=349741 GN=Amuc_2070 PE=3 SV=1	Amuc_2070	17	17	29.60	84.03	751	146.98	4.E+09	296	1.E+08
B2UR10	tr|B2UR10|B2UR10_AKKM8 Adenosylhomocysteinase OS=Akkermansia muciniphila (strain ATCC BAA-835 / DSM 22959 / JCM 33894 / BCRC 81048 / CCUG 64013 / CIP 107961 / Muc) OX=349741 GN=ahcY PE=3 SV=1	Amuc_1069	7	7	23.10	52.07	471	123.87	3.E+09	214	1.E+08

B2UPH1	tr|B2UPH1|B2UPH1_AKKM8 Phosphoribosyl-AMP cyclohydrolase OS=Akkermansia muciniphila (strain ATCC BAA-835 / DSM 22959 / JCM 33894 / BCRC 81048 / CCUG 64013 / CIP 107961 / Muc) OX=349741 GN=hisI PE=3 SV=1	Amuc_0611	3	3	23.30	17.86	163	9.66	8.E+08	52	1.E+08
B2UN13	tr|B2UN13|B2UN13_AKKM8 Carboxymuconolactone decarboxylase OS=Akkermansia muciniphila (strain ATCC BAA-835 / DSM 22959 / JCM 33894 / BCRC 81048 / CCUG 64013 / CIP 107961 / Muc) OX=349741 GN=Amuc_1806 PE=4 SV=1	Amuc_1806	6	6	29.40	28.21	255	94.40	2.E+09	161	1.E+08
B2ULU8	tr|B2ULU8|B2ULU8_AKKM8 Saccharopine dehydrogenase OS=Akkermansia muciniphila (strain ATCC BAA-835 / DSM 22959 / JCM 33894 / BCRC 81048 / CCUG 64013 / CIP 107961 / Muc) OX=349741 GN=Amuc_0148 PE=4 SV=1	Amuc_0148	7	7	28.10	45.82	409	119.97	2.E+09	185	1.E+08
B2UKV6	sp|B2UKV6|DNAK_AKKM8 Chaperone protein DnaK OS=Akkermansia muciniphila (strain ATCC BAA-835 / DSM 22959 / JCM 33894 / BCRC 81048 / CCUG 64013 / CIP 107961 / Muc) OX=349741 GN=dnaK PE=3 SV=1	Amuc_1406	13	13	29.30	68.85	642	196.07	3.E+09	365	1.E+08
B2UR52	sp|B2UR52|ILVD_AKKM8 Dihydroxy-acid dehydratase OS=Akkermansia muciniphila (strain ATCC BAA-835 / DSM 22959 / JCM 33894 / BCRC 81048 / CCUG 64013 / CIP 107961 / Muc) OX=349741 GN=ilvD PE=3 SV=1	Amuc_1111	9	9	24.60	59.48	558	87.53	2.E+09	96	1.E+08
B2UPP1	tr|B2UPP1|B2UPP1_AKKM8 Orotate phosphoribosyltransferase OS=Akkermansia muciniphila (strain ATCC BAA-835 / DSM 22959 / JCM 33894 / BCRC 81048 / CCUG 64013 / CIP 107961 / Muc) OX=349741 GN=pyrE PE=3 SV=1	Amuc_2110	4	4	33.90	20.13	189	46.38	9.E+08	30	1.E+08
B2UQ23	tr|B2UQ23|B2UQ23_AKKM8 Fructose-bisphosphate aldolase OS=Akkermansia muciniphila (strain ATCC BAA-835 / DSM 22959 / JCM 33894 / BCRC 81048 / CCUG 64013 / CIP 107961 / Muc) OX=349741 GN=Amuc_0721 PE=3 SV=1	Amuc_0721	7	7	33.20	36.92	343	133.49	2.E+09	164	1.E+08
B2UPS6	tr|B2UPS6|B2UPS6_AKKM8 6-carboxy-5,6,7,8-tetrahydropterin synthase OS=Akkermansia muciniphila (strain ATCC BAA-835 / DSM 22959 / JCM 33894 / BCRC 81048 / CCUG 64013 / CIP 107961 / Muc) OX=349741 GN=Amuc_2146 PE=3 SV=1	Amuc_2146	4	4	32.70	17.24	150	29.24	9.E+08	74	1.E+08
B2UNH4	sp|B2UNH4|GCSP_AKKM8 Glycine dehydrogenase (decarboxylating) OS=Akkermansia muciniphila (strain ATCC BAA-835 / DSM 22959 / JCM 33894 / BCRC 81048 / CCUG 64013 / CIP 107961 / Muc) OX=349741 GN=gcvP PE=3 SV=1	Amuc_0448	12	12	15.80	102.65	948	167.26	4.E+09	140	1.E+08
B2UM95	tr|B2UM95|B2UM95_AKKM8 Methylmalonyl-CoA epimerase OS=Akkermansia muciniphila (strain ATCC BAA-835 / DSM 22959 / JCM 33894 / BCRC 81048 / CCUG 64013 / CIP 107961 / Muc) OX=349741 GN=Amuc_0200 PE=3 SV=1	Amuc_0200	2	2	19.30	15.05	140	16.76	8.E+08	49	1.E+08
B2UL88	tr|B2UL88|B2UL88_AKKM8 APH domain-containing protein OS=Akkermansia muciniphila (strain ATCC BAA-835 / DSM 22959 / JCM 33894 / BCRC 81048 / CCUG 64013 / CIP 107961 / Muc) OX=349741 GN=Amuc_0030 PE=4 SV=1	Amuc_0030	10	10	31.60	41.66	364	98.24	3.E+09	286	1.E+08
B2UQP6	tr|B2UQP6|B2UQP6_AKKM8 Peptidyl-prolyl cis-trans isomerase OS=Akkermansia muciniphila (strain ATCC BAA-835 / DSM 22959 / JCM 33894 / BCRC 81048 / CCUG 64013 / CIP 107961 / Muc) OX=349741 GN=Amuc_0949 PE=3 SV=1	Amuc_0949	2	2	12.70	20.97	197	47.03	6.E+08	127	1.E+08
B2UM88	tr|B2UM88|B2UM88_AKKM8 Cysteine desulfurase OS=Akkermansia muciniphila (strain ATCC BAA-835 / DSM 22959 / JCM 33894 / BCRC 81048 / CCUG 64013 / CIP 107961 / Muc) OX=349741 GN=Amuc_0193 PE=3 SV=1	Amuc_0193	10	10	37.30	44.14	408	88.82	2.E+09	151	1.E+08
B2UL59	tr|B2UL59|B2UL59_AKKM8 NUDIX hydrolase OS=Akkermansia muciniphila (strain ATCC BAA-835 / DSM 22959 / JCM 33894 / BCRC 81048 / CCUG 64013 / CIP 107961 / Muc) OX=349741 GN=Amuc_0001 PE=4 SV=1	Amuc_0001	3	3	27.40	20.60	186	60.00	7.E+08	60	9.E+07
B2UNG5	sp|B2UNG5|RS13_AKKM8 30S ribosomal protein S13 OS=Akkermansia muciniphila (strain ATCC BAA-835 / DSM 22959 / JCM 33894 / BCRC 81048 / CCUG 64013 / CIP 107961 / Muc) OX=349741 GN=rpsM PE=3 SV=1	Amuc_0439	3	3	31.20	14.21	125	31.36	6.E+08	29	9.E+07
B2UMU1	sp|B2UMU1|RS10_AKKM8 30S ribosomal protein S10 OS=Akkermansia muciniphila (strain ATCC BAA-835 / DSM 22959 / JCM 33894 / BCRC 81048 / CCUG 64013 / CIP 107961 / Muc) OX=349741 GN=rpsJ PE=3 SV=1	Amuc_0305	2	2	21.60	11.71	102	13.75	4.E+08	35	9.E+07
B2UN72	tr|B2UN72|B2UN72_AKKM8 Glycosyl transferase group 1 OS=Akkermansia muciniphila (strain ATCC BAA-835 / DSM 22959 / JCM 33894 / BCRC 81048 / CCUG 64013 / CIP 107961 / Muc) OX=349741 GN=Amuc_1869 PE=4 SV=1	Amuc_1869	12	12	30.10	47.87	432	120.81	2.E+09	139	9.E+07

B2UM63	tr|B2UM63|B2UM63_AKKM8 Dihydrolipoyl dehydrogenase OS=Akkermansia muciniphila (strain ATCC BAA-835 / DSM 22959 / JCM 33894 / BCRC 81048 / CCUG 64013 / CIP 107961 / Muc) OX=349741 GN=Amuc_1689 PE=3 SV=1	Amuc_1689	9	9	25.30	48.95	462	92.78	2.E+09	159	9.E+07
B2ULD3	tr|B2ULD3|B2ULD3_AKKM8 2-amino-3-ketobutyrate coenzyme A ligase OS=Akkermansia muciniphila (strain ATCC BAA-835 / DSM 22959 / JCM 33894 / BCRC 81048 / CCUG 64013 / CIP 107961 / Muc) OX=349741 GN=kbl PE=3 SV=1	Amuc_0077	8	8	27.60	43.36	399	102.03	2.E+09	156	9.E+07
B2UQY3	sp|B2UQY3|RL7_AKKM8 50S ribosomal protein L7/L12 OS=Akkermansia muciniphila (strain ATCC BAA-835 / DSM 22959 / JCM 33894 / BCRC 81048 / CCUG 64013 / CIP 107961 / Muc) OX=349741 GN=rplL PE=3 SV=1	Amuc_1042	3	3	27.40	12.64	124	57.71	6.E+08	60	9.E+07
B2UP74	tr|B2UP74|B2UP74_AKKM8 Uncharacterized protein OS=Akkermansia muciniphila (strain ATCC BAA-835 / DSM 22959 / JCM 33894 / BCRC 81048 / CCUG 64013 / CIP 107961 / Muc) OX=349741 GN=Amuc_2035 PE=4 SV=1	Amuc_2035	4	4	22.30	27.43	251	116.43	1.E+09	59	9.E+07
B2UNZ8	tr|B2UNZ8|B2UNZ8_AKKM8 Transketolase OS=Akkermansia muciniphila (strain ATCC BAA-835 / DSM 22959 / JCM 33894 / BCRC 81048 / CCUG 64013 / CIP 107961 / Muc) OX=349741 GN=Amuc_0530 PE=3 SV=1	Amuc_0530	17	17	33.50	72.86	677	157.84	3.E+09	296	8.E+07
B2UP35	tr|B2UP35|B2UP35_AKKM8 Tryptophanase OS=Akkermansia muciniphila (strain ATCC BAA-835 / DSM 22959 / JCM 33894 / BCRC 81048 / CCUG 64013 / CIP 107961 / Muc) OX=349741 GN=Amuc_1995 PE=3 SV=1	Amuc_1995	7	7	17.90	54.00	487	107.46	2.E+09	195	8.E+07
B2UP77	tr|B2UP77|B2UP77_AKKM8 PUR-alpha/beta/gamma DNA/RNA-binding OS=Akkermansia muciniphila (strain ATCC BAA-835 / DSM 22959 / JCM 33894 / BCRC 81048 / CCUG 64013 / CIP 107961 / Muc) OX=349741 GN=Amuc_2038 PE=4 SV=1	Amuc_2038	4	4	52.90	8.11	70	11.84	2.E+08	51	8.E+07
B2URK3	tr|B2URK3|B2URK3_AKKM8 Quinolinate phosphoribosyltransferase [decarboxylating] OS=Akkermansia muciniphila (strain ATCC BAA-835 / DSM 22959 / JCM 33894 / BCRC 81048 / CCUG 64013 / CIP 107961 / Muc) OX=349741 GN=Amuc_1263 PE=3 SV=1	Amuc_1263	3	3	13.90	30.91	287	48.31	8.E+08	108	8.E+07
B2UPP7	tr|B2UPP7|B2UPP7_AKKM8 Alcohol dehydrogenase zinc-binding domain protein OS=Akkermansia muciniphila (strain ATCC BAA-835 / DSM 22959 / JCM 33894 / BCRC 81048 / CCUG 64013 / CIP 107961 / Muc) OX=349741 GN=Amuc_2116 PE=3 SV=1	Amuc_2116	7	7	26.80	40.38	373	64.36	1.E+09	184	8.E+07
B2UKV8	sp|B2UKV8|CH60_AKKM8 60 kDa chaperonin OS=Akkermansia muciniphila (strain ATCC BAA-835 / DSM 22959 / JCM 33894 / BCRC 81048 / CCUG 64013 / CIP 107961 / Muc) OX=349741 GN=groL PE=3 SV=1	Amuc_1408	11	11	29.10	58.44	550	157.91	3.E+09	247	7.E+07
B2UM62	tr|B2UM62|B2UM62_AKKM8 Amidohydrolase OS=Akkermansia muciniphila (strain ATCC BAA-835 / DSM 22959 / JCM 33894 / BCRC 81048 / CCUG 64013 / CIP 107961 / Muc) OX=349741 GN=Amuc_1688 PE=4 SV=1	Amuc_1688	4	4	15.20	47.60	440	55.44	1.E+09	55	7.E+07
B2UPW3	tr|B2UPW3|B2UPW3_AKKM8 D-alanine--D-alanine ligase OS=Akkermansia muciniphila (strain ATCC BAA-835 / DSM 22959 / JCM 33894 / BCRC 81048 / CCUG 64013 / CIP 107961 / Muc) OX=349741 GN=ddl PE=3 SV=1	Amuc_0661	3	3	10.30	33.30	311	23.44	1.E+09	89	7.E+07
B2UNP4	tr|B2UNP4|B2UNP4_AKKM8 N-acylglucosamine 2-epimerase OS=Akkermansia muciniphila (strain ATCC BAA-835 / DSM 22959 / JCM 33894 / BCRC 81048 / CCUG 64013 / CIP 107961 / Muc) OX=349741 GN=Amuc_1947 PE=4 SV=1	Amuc_1947	6	6	20.60	45.60	393	31.79	1.E+09	124	6.E+07
B2URK6	tr|B2URK6|B2URK6_AKKM8 Uncharacterized protein OS=Akkermansia muciniphila (strain ATCC BAA-835 / DSM 22959 / JCM 33894 / BCRC 81048 / CCUG 64013 / CIP 107961 / Muc) OX=349741 GN=Amuc_1266 PE=4 SV=1	Amuc_1266	1	1	7.70	22.40	220	5.69	1.E+08	7	6.E+07
B2URQ1	tr|B2URQ1|B2URQ1_AKKM8 Peptidase M16 domain protein OS=Akkermansia muciniphila (strain ATCC BAA-835 / DSM 22959 / JCM 33894 / BCRC 81048 / CCUG 64013 / CIP 107961 / Muc) OX=349741 GN=Amuc_1312 PE=4 SV=1	Amuc_1312	9	9	14.90	93.01	840	150.12	2.E+09	211	6.E+07
B2UQL1	tr|B2UQL1|B2UQL1_AKKM8 Cupin 2 conserved barrel domain protein OS=Akkermansia muciniphila (strain ATCC BAA-835 / DSM 22959 / JCM 33894 / BCRC 81048 / CCUG 64013 / CIP 107961 / Muc) OX=349741 GN=Amuc_0914 PE=4 SV=1	Amuc_0914	2	2	18.80	12.31	112	11.16	3.E+08	34	6.E+07
B2UNJ7	tr|B2UNJ7|B2UNJ7_AKKM8 Flavodoxin/nitric oxide synthase OS=Akkermansia muciniphila (strain ATCC BAA-835 / DSM 22959 / JCM 33894 / BCRC 81048 / CCUG 64013 / CIP 107961 / Muc) OX=349741 GN=Amuc_1899 PE=4 SV=1	Amuc_1899	2	2	14.60	15.84	144	16.15	3.E+08	31	6.E+07
B2UN86	tr|B2UN86|B2UN86_AKKM8 Beta-lactamase domain protein OS=Akkermansia muciniphila (strain ATCC BAA-835 / DSM 22959 / JCM 33894 / BCRC 81048 / CCUG 64013 / CIP 107961 / Muc) OX=349741 GN=Amuc_1883 PE=4 SV=1	Amuc_1883	7	7	20.20	50.21	456	38.43	1.E+09	90	6.E+07

B2UPT0	tr|B2UPT0|B2UPT0_AKKM8 Quinolinate synthase OS=Akkermansia muciniphila (strain ATCC BAA-835 / DSM 22959 / JCM 33894 / BCRC 81048 / CCUG 64013 / CIP 107961 / Muc) OX=349741 GN=Amuc_2150 PE=4 SV=1	Amuc_2150	3	3	15.80	34.45	311	47.91	1.E+09	59	6.E+07
B2UKP4	tr|B2UKP4|B2UKP4_AKKM8 Uncharacterized protein OS=Akkermansia muciniphila (strain ATCC BAA-835 / DSM 22959 / JCM 33894 / BCRC 81048 / CCUG 64013 / CIP 107961 / Muc) OX=349741 GN=Amuc_1343 PE=4 SV=1	Amuc_1343	2	2	13.30	20.32	188	12.68	4.E+08	41	5.E+07
B2UNX2	tr|B2UNX2|B2UNX2_AKKM8 DUF5069 domain-containing protein OS=Akkermansia muciniphila (strain ATCC BAA-835 / DSM 22959 / JCM 33894 / BCRC 81048 / CCUG 64013 / CIP 107961 / Muc) OX=349741 GN=Amuc_0504 PE=4 SV=1	Amuc_0504	2	2	14.40	21.45	187	13.69	5.E+08	100	5.E+07
B2UMI8	tr|B2UMI8|B2UMI8_AKKM8 UDP-glucuronate decarboxylase OS=Akkermansia muciniphila (strain ATCC BAA-835 / DSM 22959 / JCM 33894 / BCRC 81048 / CCUG 64013 / CIP 107961 / Muc) OX=349741 GN=Amuc_1726 PE=3 SV=1	Amuc_1726	6	6	20.60	35.03	310	47.48	8.E+08	163	5.E+07
B2UPX3	tr|B2UPX3|B2UPX3_AKKM8 3-dehydroquinate synthase OS=Akkermansia muciniphila (strain ATCC BAA-835 / DSM 22959 / JCM 33894 / BCRC 81048 / CCUG 64013 / CIP 107961 / Muc) OX=349741 GN=Amuc_0671 PE=4 SV=1	Amuc_0671	4	4	13.20	42.15	387	47.64	1.E+09	121	5.E+07
B2URI5	tr|B2URI5|B2URI5_AKKM8 Mannose-6-phosphate isomerase OS=Akkermansia muciniphila (strain ATCC BAA-835 / DSM 22959 / JCM 33894 / BCRC 81048 / CCUG 64013 / CIP 107961 / Muc) OX=349741 GN=Amuc_1245 PE=3 SV=1	Amuc_1245	8	8	32.70	34.83	306	39.99	7.E+08	60	5.E+07
B2UNZ3	tr|B2UNZ3|B2UNZ3_AKKM8 Cycloartenol synthase-like protein OS=Akkermansia muciniphila (strain ATCC BAA-835 / DSM 22959 / JCM 33894 / BCRC 81048 / CCUG 64013 / CIP 107961 / Muc) OX=349741 GN=Amuc_0525 PE=4 SV=1	Amuc_0525	8	8	30.40	43.36	392	42.09	1.E+09	69	5.E+07
B2UM18	tr|B2UM18|B2UM18_AKKM8 Enoyl-[acyl-carrier-protein] reductase [NADH] OS=Akkermansia muciniphila (strain ATCC BAA-835 / DSM 22959 / JCM 33894 / BCRC 81048 / CCUG 64013 / CIP 107961 / Muc) OX=349741 GN=Amuc_1642 PE=3 SV=1	Amuc_1642	5	5	19.60	27.88	260	26.48	8.E+08	75	5.E+07
B2UKX4	tr|B2UKX4|B2UKX4_AKKM8 Kelch repeat-containing protein OS=Akkermansia muciniphila (strain ATCC BAA-835 / DSM 22959 / JCM 33894 / BCRC 81048 / CCUG 64013 / CIP 107961 / Muc) OX=349741 GN=Amuc_1425 PE=4 SV=1	Amuc_1425	2	2	9.20	35.13	327	29.39	7.E+08	56	5.E+07
B2UQX4	tr|B2UQX4|B2UQX4_AKKM8 Sulfatase OS=Akkermansia muciniphila (strain ATCC BAA-835 / DSM 22959 / JCM 33894 / BCRC 81048 / CCUG 64013 / CIP 107961 / Muc) OX=349741 GN=Amuc_1033 PE=3 SV=1	Amuc_1033	9	9	18.20	67.00	581	42.47	2.E+09	103	5.E+07
B2ULX5	tr|B2ULX5|B2ULX5_AKKM8 PDZ/DHR/GLGF domain protein OS=Akkermansia muciniphila (strain ATCC BAA-835 / DSM 22959 / JCM 33894 / BCRC 81048 / CCUG 64013 / CIP 107961 / Muc) OX=349741 GN=Amuc_0175 PE=4 SV=1	Amuc_0175	1	1	4.40	42.68	390	27.86	8.E+08	20	5.E+07
B2UM28	tr|B2UM28|B2UM28_AKKM8 Ribulose-phosphate 3-epimerase OS=Akkermansia muciniphila (strain ATCC BAA-835 / DSM 22959 / JCM 33894 / BCRC 81048 / CCUG 64013 / CIP 107961 / Muc) OX=349741 GN=rpe PE=3 SV=1	Amuc_1653	3	3	12.40	23.63	217	15.85	2.E+08	12	5.E+07
B2UPD6	tr|B2UPD6|B2UPD6_AKKM8 Peptidase M16 domain protein OS=Akkermansia muciniphila (strain ATCC BAA-835 / DSM 22959 / JCM 33894 / BCRC 81048 / CCUG 64013 / CIP 107961 / Muc) OX=349741 GN=Amuc_0576 PE=3 SV=1	Amuc_0576	22	22	16.90	160.48	1442	199.61	4.E+09	264	5.E+07
B2UQ36	tr|B2UQ36|B2UQ36_AKKM8 YD repeat protein OS=Akkermansia muciniphila (strain ATCC BAA-835 / DSM 22959 / JCM 33894 / BCRC 81048 / CCUG 64013 / CIP 107961 / Muc) OX=349741 GN=Amuc_0735 PE=4 SV=1	Amuc_0735	23	23	19.20	212.82	1938	161.17	4.E+09	172	4.E+07
B2UQY5	tr|B2UQY5|B2UQY5_AKKM8 50S ribosomal protein L1 OS=Akkermansia muciniphila (strain ATCC BAA-835 / DSM 22959 / JCM 33894 / BCRC 81048 / CCUG 64013 / CIP 107961 / Muc) OX=349741 GN=rplA PE=3 SV=1	Amuc_1044	4	4	26.60	24.67	233	41.16	5.E+08	34	4.E+07
B2UQJ3	tr|B2UQJ3|B2UQJ3_AKKM8 PepSY_like domain-containing protein OS=Akkermansia muciniphila (strain ATCC BAA-835 / DSM 22959 / JCM 33894 / BCRC 81048 / CCUG 64013 / CIP 107961 / Muc) OX=349741 GN=Amuc_0895 PE=4 SV=1	Amuc_0895	3	3	24.30	16.87	152	8.63	3.E+08	24	4.E+07
B2UMU3	sp|B2UMU3|RS7_AKKM8 30S ribosomal protein S7 OS=Akkermansia muciniphila (strain ATCC BAA-835 / DSM 22959 / JCM 33894 / BCRC 81048 / CCUG 64013 / CIP 107961 / Muc) OX=349741 GN=rpsG PE=3 SV=1	Amuc_0307	2	2	25.50	17.94	157	28.58	3.E+08	42	4.E+07
B2UL08	tr|B2UL08|B2UL08_AKKM8 Protein GrpE OS=Akkermansia muciniphila (strain ATCC BAA-835 / DSM 22959 / JCM 33894 / BCRC 81048 / CCUG 64013 / CIP 107961 / Muc) OX=349741 GN=grpE PE=3 SV=1	Amuc_1459	2	2	17.90	20.67	184	32.19	2.E+08	16	4.E+07

B2UP30	tr|B2UP30|B2UP30_AKKM8 Pyrroline-5-carboxylate reductase OS=Akkermansia muciniphila (strain ATCC BAA-835 / DSM 22959 / JCM 33894 / BCRC 81048 / CCUG 64013 / CIP 107961 / Muc) OX=349741 GN=proC PE=3 SV=1	Amuc_1990	3	3	15.70	27.92	268	30.52	5.E+08	43	4.E+07
B2URK5	tr|B2URK5|B2URK5_AKKM8 Aspartate-semialdehyde dehydrogenase OS=Akkermansia muciniphila (strain ATCC BAA-835 / DSM 22959 / JCM 33894 / BCRC 81048 / CCUG 64013 / CIP 107961 / Muc) OX=349741 GN=asd PE=3 SV=1	Amuc_1265	4	4	16.70	36.06	336	98.04	7.E+08	38	4.E+07
B2UKZ7	tr|B2UKZ7|B2UKZ7_AKKM8 Arginine biosynthesis bifunctional protein ArgJ OS=Akkermansia muciniphila (strain ATCC BAA-835 / DSM 22959 / JCM 33894 / BCRC 81048 / CCUG 64013 / CIP 107961 / Muc) OX=349741 GN=argJ PE=3 SV=1	Amuc_1448	6	6	19.70	44.44	417	35.58	9.E+08	51	4.E+07
B2UQI1	tr|B2UQI1|B2UQI1_AKKM8 Uncharacterized protein OS=Akkermansia muciniphila (strain ATCC BAA-835 / DSM 22959 / JCM 33894 / BCRC 81048 / CCUG 64013 / CIP 107961 / Muc) OX=349741 GN=Amuc_0883 PE=4 SV=1	Amuc_0883	2	2	21.00	6.74	62	29.80	8.E+07	4	4.E+07
B2UN37	tr|B2UN37|B2UN37_AKKM8 L-fuculokinase OS=Akkermansia muciniphila (strain ATCC BAA-835 / DSM 22959 / JCM 33894 / BCRC 81048 / CCUG 64013 / CIP 107961 / Muc) OX=349741 GN=fucK PE=3 SV=1	Amuc_1830	6	6	20.50	51.25	474	46.09	7.E+08	85	4.E+07
B2UP85	tr|B2UP85|B2UP85_AKKM8 Uncharacterized protein OS=Akkermansia muciniphila (strain ATCC BAA-835 / DSM 22959 / JCM 33894 / BCRC 81048 / CCUG 64013 / CIP 107961 / Muc) OX=349741 GN=Amuc_2046 PE=4 SV=1	Amuc_2046	4	4	27.70	22.81	206	27.75	5.E+08	93	4.E+07
B2UNN9	tr|B2UNN9|B2UNN9_AKKM8 Peptidase T OS=Akkermansia muciniphila (strain ATCC BAA-835 / DSM 22959 / JCM 33894 / BCRC 81048 / CCUG 64013 / CIP 107961 / Muc) OX=349741 GN=pepT PE=3 SV=1	Amuc_1942	5	5	17.70	45.12	417	37.50	6.E+08	48	4.E+07
B2URI3	sp|B2URI3|ISPF_AKKM8 2-C-methyl-D-erythritol 2,4-cyclodiphosphate synthase OS=Akkermansia muciniphila (strain ATCC BAA-835 / DSM 22959 / JCM 33894 / BCRC 81048 / CCUG 64013 / CIP 107961 / Muc) OX=349741 GN=ispF PE=3 SV=1	Amuc_1243	3	3	21.30	17.69	169	15.18	3.E+08	15	4.E+07
B2UL30	tr|B2UL30|B2UL30_AKKM8 ATP-dependent 6-phosphofructokinase OS=Akkermansia muciniphila (strain ATCC BAA-835 / DSM 22959 / JCM 33894 / BCRC 81048 / CCUG 64013 / CIP 107961 / Muc) OX=349741 GN=pfkA PE=3 SV=1	Amuc_1481	5	5	15.90	37.95	359	14.12	7.E+08	53	4.E+07
B2UMH5	sp|B2UMH5|SUCC_AKKM8 Succinate--CoA ligase [ADP-forming] subunit beta OS=Akkermansia muciniphila (strain ATCC BAA-835 / DSM 22959 / JCM 33894 / BCRC 81048 / CCUG 64013 / CIP 107961 / Muc) OX=349741 GN=sucC PE=3 SV=1	Amuc_1713	6	6	20.40	42.34	393	102.02	1.E+09	77	4.E+07
B2UMH4	tr|B2UMH4|B2UMH4_AKKM8 Succinate--CoA ligase [ADP-forming] subunit alpha OS=Akkermansia muciniphila (strain ATCC BAA-835 / DSM 22959 / JCM 33894 / BCRC 81048 / CCUG 64013 / CIP 107961 / Muc) OX=349741 GN=sucD PE=3 SV=1	Amuc_1712	4	4	18.60	30.57	295	20.75	5.E+08	39	4.E+07
B2UP52	tr|B2UP52|B2UP52_AKKM8 O-acetylhomoserine/O-acetylserine sulfhydrylase OS=Akkermansia muciniphila (strain ATCC BAA-835 / DSM 22959 / JCM 33894 / BCRC 81048 / CCUG 64013 / CIP 107961 / Muc) OX=349741 GN=Amuc_2013 PE=3 SV=1	Amuc_2013	3	3	8.40	46.41	428	32.68	7.E+08	51	4.E+07
B2UQI4	tr|B2UQI4|B2UQI4_AKKM8 Transketolase central region OS=Akkermansia muciniphila (strain ATCC BAA-835 / DSM 22959 / JCM 33894 / BCRC 81048 / CCUG 64013 / CIP 107961 / Muc) OX=349741 GN=Amuc_0886 PE=4 SV=1	Amuc_0886	7	7	30.90	35.59	324	53.97	6.E+08	81	4.E+07
B2UQG5	tr|B2UQG5|B2UQG5_AKKM8 Peptidase M42 family protein OS=Akkermansia muciniphila (strain ATCC BAA-835 / DSM 22959 / JCM 33894 / BCRC 81048 / CCUG 64013 / CIP 107961 / Muc) OX=349741 GN=Amuc_0867 PE=3 SV=1	Amuc_0867	5	5	19.10	41.20	382	37.51	7.E+08	38	3.E+07
B2UNI3	tr|B2UNI3|B2UNI3_AKKM8 AIR synthase OS=Akkermansia muciniphila (strain ATCC BAA-835 / DSM 22959 / JCM 33894 / BCRC 81048 / CCUG 64013 / CIP 107961 / Muc) OX=349741 GN=Amuc_0457 PE=3 SV=1	Amuc_0457	5	5	20.10	36.49	333	66.15	6.E+08	55	3.E+07
B2UN30;B2 UQW1	tr|B2UN30|B2UN30_AKKM8 Uncharacterized protein OS=Akkermansia muciniphila (strain ATCC BAA-835 / DSM 22959 / JCM 33894 / BCRC 81048 / CCUG 64013 / CIP 107961 / Muc) OX=349741 GN=Amuc_1823 PE=4 SV=1;tr|B2UQW1|B2UQW1_AKKM8 Uncharacterized protein OS=Akkerman	Amuc_1823	6	6	15.40	61.69	545;546	132.90	9.E+08	75	3.E+07
B2UMD4	tr|B2UMD4|B2UMD4_AKKM8 Sec-independent protein translocase protein TatA OS=Akkermansia muciniphila (strain ATCC BAA-835 / DSM 22959 / JCM 33894 / BCRC 81048 / CCUG 64013 / CIP 107961 / Muc) OX=349741 GN=tatA PE=3 SV=1	Amuc_0240	1	1	19.20	10.90	99	2.87	1.E+08	8	3.E+07
B2UQC2	tr|B2UQC2|B2UQC2_AKKM8 Lactase OS=Akkermansia muciniphila (strain ATCC BAA-835 / DSM 22959 / JCM 33894 / BCRC 81048 / CCUG 64013 / CIP 107961 / Muc) OX=349741 GN=Amuc_0824 PE=3 SV=1	Amuc_0824	20	20	18.00	142.05	1264	124.73	2.E+09	273	3.E+07

B2UNT6	tr|B2UNT6|B2UNT6_AKKM8 Transcriptional regulator, DeoR family OS=Akkermansia muciniphila (strain ATCC BAA-835 / DSM 22959 / JCM 33894 / BCRC 81048 / CCUG 64013 / CIP 107961 / Muc) OX=349741 GN=Amuc_0468 PE=4 SV=1	Amuc_0468	3	3	14.10	27.12	249	15.20	4.E+08	30	3.E+07
B2UQG3	tr|B2UQG3|B2UQG3_AKKM8 Rubrerythrin OS=Akkermansia muciniphila (strain ATCC BAA-835 / DSM 22959 / JCM 33894 / BCRC 81048 / CCUG 64013 / CIP 107961 / Muc) OX=349741 GN=Amuc_0865 PE=4 SV=1	Amuc_0865	4	4	29.70	19.53	182	35.11	3.E+08	31	3.E+07
B2URJ1	tr|B2URJ1|B2URJ1_AKKM8 Carbamoyl-phosphate synthase large chain OS=Akkermansia muciniphila (strain ATCC BAA-835 / DSM 22959 / JCM 33894 / BCRC 81048 / CCUG 64013 / CIP 107961 / Muc) OX=349741 GN=carB PE=3 SV=1	Amuc_1251	16	16	21.00	117.47	1067	120.87	2.E+09	230	3.E+07
B2UQE2	tr|B2UQE2|B2UQE2_AKKM8 Enolase OS=Akkermansia muciniphila (strain ATCC BAA-835 / DSM 22959 / JCM 33894 / BCRC 81048 / CCUG 64013 / CIP 107961 / Muc) OX=349741 GN=eno PE=3 SV=1	Amuc_0844	6	4	19.30	45.97	429	25.17	5.E+08	26	3.E+07
B2UP25	sp|B2UP25|RS14_AKKM8 30S ribosomal protein S14 OS=Akkermansia muciniphila (strain ATCC BAA-835 / DSM 22959 / JCM 33894 / BCRC 81048 / CCUG 64013 / CIP 107961 / Muc) OX=349741 GN=rpsN PE=3 SV=1	Amuc_0557	2	2	25.80	10.28	89	7.50	6.E+07	15	3.E+07
B2UL21	sp|B2UL21|RL25_AKKM8 50S ribosomal protein L25 OS=Akkermansia muciniphila (strain ATCC BAA-835 / DSM 22959 / JCM 33894 / BCRC 81048 / CCUG 64013 / CIP 107961 / Muc) OX=349741 GN=rplY PE=3 SV=1	Amuc_1472	2	2	15.40	21.01	201	14.15	2.E+08	31	3.E+07
B2UME2	tr|B2UME2|B2UME2_AKKM8 Nitroreductase OS=Akkermansia muciniphila (strain ATCC BAA-835 / DSM 22959 / JCM 33894 / BCRC 81048 / CCUG 64013 / CIP 107961 / Muc) OX=349741 GN=Amuc_0248 PE=4 SV=1	Amuc_0248	1	1	7.30	20.92	193	35.31	2.E+08	30	3.E+07
B2UN14	tr|B2UN14|B2UN14_AKKM8 Aldo/keto reductase OS=Akkermansia muciniphila (strain ATCC BAA-835 / DSM 22959 / JCM 33894 / BCRC 81048 / CCUG 64013 / CIP 107961 / Muc) OX=349741 GN=Amuc_1807 PE=4 SV=1	Amuc_1807	7	7	33.40	34.57	314	25.97	5.E+08	86	3.E+07
B2UQC0	tr|B2UQC0|B2UQC0_AKKM8 TatD-related deoxyribonuclease OS=Akkermansia muciniphila (strain ATCC BAA-835 / DSM 22959 / JCM 33894 / BCRC 81048 / CCUG 64013 / CIP 107961 / Muc) OX=349741 GN=Amuc_0822 PE=4 SV=1	Amuc_0822	4	4	19.20	30.49	271	25.97	5.E+08	44	3.E+07
B2UQQ6	tr|B2UQQ6|B2UQQ6_AKKM8 dITP/XTP pyrophosphatase OS=Akkermansia muciniphila (strain ATCC BAA-835 / DSM 22959 / JCM 33894 / BCRC 81048 / CCUG 64013 / CIP 107961 / Muc) OX=349741 GN=Amuc_0959 PE=3 SV=1	Amuc_0959	3	3	21.00	23.54	214	12.43	2.E+08	34	3.E+07
B2UNA9	sp|B2UNA9|PNP_AKKM8 Polyribonucleotide nucleotidyltransferase OS=Akkermansia muciniphila (strain ATCC BAA-835 / DSM 22959 / JCM 33894 / BCRC 81048 / CCUG 64013 / CIP 107961 / Muc) OX=349741 GN=pnp PE=3 SV=1	Amuc_0382	8	8	15.80	77.48	714	35.83	9.E+08	84	3.E+07
B2URG3	tr|B2URG3|B2URG3_AKKM8 Orotidine-5-phosphate decarboxylase OS=Akkermansia muciniphila (strain ATCC BAA-835 / DSM 22959 / JCM 33894 / BCRC 81048 / CCUG 64013 / CIP 107961 / Muc) OX=349741 GN=Amuc_1223 PE=4 SV=1	Amuc_1223	4	4	22.70	28.31	264	47.74	4.E+08	49	3.E+07
B2UN11	tr|B2UN11|B2UN11_AKKM8 Flavodoxin-like domain-containing protein OS=Akkermansia muciniphila (strain ATCC BAA-835 / DSM 22959 / JCM 33894 / BCRC 81048 / CCUG 64013 / CIP 107961 / Muc) OX=349741 GN=Amuc_1804 PE=4 SV=1	Amuc_1804	2	2	14.00	19.22	178	8.61	3.E+08	16	3.E+07
B2URD0	tr|B2URD0|B2URD0_AKKM8 Alcohol dehydrogenase zinc-binding domain protein OS=Akkermansia muciniphila (strain ATCC BAA-835 / DSM 22959 / JCM 33894 / BCRC 81048 / CCUG 64013 / CIP 107961 / Muc) OX=349741 GN=Amuc_1190 PE=4 SV=1	Amuc_1190	6	6	25.50	36.08	333	43.43	5.E+08	62	3.E+07
B2UMF9	sp|B2UMF9|PLSX_AKKM8 Phosphate acyltransferase OS=Akkermansia muciniphila (strain ATCC BAA-835 / DSM 22959 / JCM 33894 / BCRC 81048 / CCUG 64013 / CIP 107961 / Muc) OX=349741 GN=plsX PE=3 SV=1	Amuc_0265	1	1	4.30	37.05	349	5.05	5.E+08	27	3.E+07
B2ULH7	tr|B2ULH7|B2ULH7_AKKM8 Formate C-acetyltransferase OS=Akkermansia muciniphila (strain ATCC BAA-835 / DSM 22959 / JCM 33894 / BCRC 81048 / CCUG 64013 / CIP 107961 / Muc) OX=349741 GN=Amuc_1543 PE=3 SV=1	Amuc_1543	11	11	20.70	85.23	755	80.99	1.E+09	158	2.E+07
B2ULM1	tr|B2ULM1|B2ULM1_AKKM8 Uncharacterized protein OS=Akkermansia muciniphila (strain ATCC BAA-835 / DSM 22959 / JCM 33894 / BCRC 81048 / CCUG 64013 / CIP 107961 / Muc) OX=349741 GN=Amuc_1588 PE=4 SV=1	Amuc_1588	4	4	27.80	18.54	169	21.44	2.E+08	29	2.E+07
B2UQI5	tr|B2UQI5|B2UQI5_AKKM8 Dehydrogenase E1 component OS=Akkermansia muciniphila (strain ATCC BAA-835 / DSM 22959 / JCM 33894 / BCRC 81048 / CCUG 64013 / CIP 107961 / Muc) OX=349741 GN=Amuc_0887 PE=4 SV=1	Amuc_0887	7	7	23.70	33.92	312	28.90	4.E+08	50	2.E+07

B2ULE0	tr|B2ULE0|B2ULE0_AKKM8 PA14 domain-containing protein OS=Akkermansia muciniphila (strain ATCC BAA-835 / DSM 22959 / JCM 33894 / BCRC 81048 / CCUG 64013 / CIP 107961 / Muc) OX=349741 GN=Amuc_0084 PE=4 SV=1	Amuc_0084	3	3	9.90	61.62	547	47.40	7.E+08	49	2.E+07
B2URI2	tr|B2URI2|B2URI2_AKKM8 Inositol-1-monophosphatase OS=Akkermansia muciniphila (strain ATCC BAA-835 / DSM 22959 / JCM 33894 / BCRC 81048 / CCUG 64013 / CIP 107961 / Muc) OX=349741 GN=Amuc_1242 PE=3 SV=1	Amuc_1242	3	3	14.20	28.52	254	11.42	2.E+08	27	2.E+07
B2ULB4	tr|B2ULB4|B2ULB4_AKKM8 UvrB/UvrC protein OS=Akkermansia muciniphila (strain ATCC BAA-835 / DSM 22959 / JCM 33894 / BCRC 81048 / CCUG 64013 / CIP 107961 / Muc) OX=349741 GN=Amuc_0057 PE=4 SV=1	Amuc_0057	1	1	7.30	19.74	178	8.37	1.E+08	15	2.E+07
B2UKU8	tr|B2UKU8|B2UKU8_AKKM8 Histidine--tRNA ligase OS=Akkermansia muciniphila (strain ATCC BAA-835 / DSM 22959 / JCM 33894 / BCRC 81048 / CCUG 64013 / CIP 107961 / Muc) OX=349741 GN=Amuc_1397 PE=3 SV=1	Amuc_1397	7	7	20.50	47.05	424	40.52	5.E+08	66	2.E+07
B2UN97	sp|B2UN97|AROA_AKKM8 3-phosphoshikimate 1-carboxyvinyltransferase OS=Akkermansia muciniphila (strain ATCC BAA-835 / DSM 22959 / JCM 33894 / BCRC 81048 / CCUG 64013 / CIP 107961 / Muc) OX=349741 GN=aroA PE=3 SV=1	Amuc_1894	3	3	11.50	46.11	435	19.31	3.E+08	17	2.E+07
B2UL94	tr|B2UL94|B2UL94_AKKM8 YD repeat protein OS=Akkermansia muciniphila (strain ATCC BAA-835 / DSM 22959 / JCM 33894 / BCRC 81048 / CCUG 64013 / CIP 107961 / Muc) OX=349741 GN=Amuc_0036 PE=4 SV=1	Amuc_0036	26	26	19.70	214.69	1957	147.05	2.E+09	211	2.E+07
B2UR15	tr|B2UR15|B2UR15_AKKM8 Sulfatase OS=Akkermansia muciniphila (strain ATCC BAA-835 / DSM 22959 / JCM 33894 / BCRC 81048 / CCUG 64013 / CIP 107961 / Muc) OX=349741 GN=Amuc_1074 PE=3 SV=1	Amuc_1074	5	5	10.10	62.82	552	18.05	5.E+08	74	2.E+07
B2UQ75	tr|B2UQ75|B2UQ75_AKKM8 Thioesterase superfamily protein OS=Akkermansia muciniphila (strain ATCC BAA-835 / DSM 22959 / JCM 33894 / BCRC 81048 / CCUG 64013 / CIP 107961 / Muc) OX=349741 GN=Amuc_0775 PE=3 SV=1	Amuc_0775	1	1	9.70	16.96	155	35.42	1.E+08	28	2.E+07
B2URJ2	tr|B2URJ2|B2URJ2_AKKM8 Glutamine synthetase catalytic region OS=Akkermansia muciniphila (strain ATCC BAA-835 / DSM 22959 / JCM 33894 / BCRC 81048 / CCUG 64013 / CIP 107961 / Muc) OX=349741 GN=Amuc_1252 PE=3 SV=1	Amuc_1252	1	1	1.80	78.73	711	2.27	6.E+08	1	2.E+07
B2UN71	tr|B2UN71|B2UN71_AKKM8 Glycoside hydrolase family 57 OS=Akkermansia muciniphila (strain ATCC BAA-835 / DSM 22959 / JCM 33894 / BCRC 81048 / CCUG 64013 / CIP 107961 / Muc) OX=349741 GN=Amuc_1868 PE=4 SV=1	Amuc_1868	5	5	15.70	45.10	395	13.43	3.E+08	14	2.E+07
B2UPB5	tr|B2UPB5|B2UPB5_AKKM8 Polysaccharide export protein OS=Akkermansia muciniphila (strain ATCC BAA-835 / DSM 22959 / JCM 33894 / BCRC 81048 / CCUG 64013 / CIP 107961 / Muc) OX=349741 GN=Amuc_2077 PE=4 SV=1	Amuc_2077	4	4	20.40	28.03	260	35.78	3.E+08	35	2.E+07
B2UM84	tr|B2UM84|B2UM84_AKKM8 Isoleucine--tRNA ligase OS=Akkermansia muciniphila (strain ATCC BAA-835 / DSM 22959 / JCM 33894 / BCRC 81048 / CCUG 64013 / CIP 107961 / Muc) OX=349741 GN=ileS PE=3 SV=1	Amuc_0189	12	12	16.80	102.39	909	47.91	9.E+08	189	2.E+07
B2UMZ3	tr|B2UMZ3|B2UMZ3_AKKM8 Uncharacterized protein OS=Akkermansia muciniphila (strain ATCC BAA-835 / DSM 22959 / JCM 33894 / BCRC 81048 / CCUG 64013 / CIP 107961 / Muc) OX=349741 GN=Amuc_0360 PE=4 SV=1	Amuc_0360	1	1	11.40	19.02	184	5.77	5.E+07	6	2.E+07
B2UNL2	tr|B2UNL2|B2UNL2_AKKM8 Site-specific DNA-methyltransferase (adenine-specific) OS=Akkermansia muciniphila (strain ATCC BAA-835 / DSM 22959 / JCM 33894 / BCRC 81048 / CCUG 64013 / CIP 107961 / Muc) OX=349741 GN=Amuc_1915 PE=3 SV=1	Amuc_1915	12	12	15.00	95.77	853	40.70	8.E+08	86	2.E+07
B2ULS7	tr|B2ULS7|B2ULS7_AKKM8 Transposase IS3/IS911 family protein OS=Akkermansia muciniphila (strain ATCC BAA-835 / DSM 22959 / JCM 33894 / BCRC 81048 / CCUG 64013 / CIP 107961 / Muc) OX=349741 GN=Amuc_0127 PE=4 SV=1	Amuc_0127	3	3	31.70	13.95	126	16.53	1.E+08	9	2.E+07
B2URJ0	sp|B2URJ0|CARA_AKKM8 Carbamoyl-phosphate synthase small chain OS=Akkermansia muciniphila (strain ATCC BAA-835 / DSM 22959 / JCM 33894 / BCRC 81048 / CCUG 64013 / CIP 107961 / Muc) OX=349741 GN=carA PE=3 SV=1	Amuc_1250	4	4	17.70	40.19	372	13.16	2.E+08	34	2.E+07
B2UPP9	tr|B2UPP9|B2UPP9_AKKM8 Nitrilase/cyanide hydratase and apolipoprotein N-acyltransferase OS=Akkermansia muciniphila (strain ATCC BAA-835 / DSM 22959 / JCM 33894 / BCRC 81048 / CCUG 64013 / CIP 107961 / Muc) OX=349741 GN=Amuc_2118 PE=4 SV=1	Amuc_2118	3	3	11.90	32.04	285	14.89	2.E+08	23	2.E+07

B2UPD0	tr|B2UPD0|B2UPD0_AKKM8 Uncharacterized protein OS=Akkermansia muciniphila (strain ATCC BAA-835 / DSM 22959 / JCM 33894 / BCRC 81048 / CCUG 64013 / CIP 107961 / Muc) OX=349741 GN=Amuc_0570 PE=4 SV=1	Amuc_0570	1	1	17.00	12.10	100	9.16	1.E+08	8	2.E+07
B2UL26	tr|B2UL26|B2UL26_AKKM8 4-hydroxythreonine-4-phosphate dehydrogenase OS=Akkermansia muciniphila (strain ATCC BAA-835 / DSM 22959 / JCM 33894 / BCRC 81048 / CCUG 64013 / CIP 107961 / Muc) OX=349741 GN=Amuc_1477 PE=4 SV=1	Amuc_1477	3	3	17.30	31.16	289	16.71	2.E+08	27	2.E+07
B2UPT2;B2UQS7	tr|B2UPT2|B2UPT2_AKKM8 YD repeat protein OS=Akkermansia muciniphila (strain ATCC BAA-835 / DSM 22959 / JCM 33894 / BCRC 81048 / CCUG 64013 / CIP 107961 / Muc) OX=349741 GN=Amuc_2152 PE=4 SV=1;tr|B2UQS7|B2UQS7_AKKM8 YD repeat protein OS=Akkermansia muciniph	Amuc_2152	19	19	14.20	208.44	1912;1929	76.75	1.E+09	112	2.E+07
B2UPC8	tr|B2UPC8|B2UPC8_AKKM8 Thioesterase superfamily protein OS=Akkermansia muciniphila (strain ATCC BAA-835 / DSM 22959 / JCM 33894 / BCRC 81048 / CCUG 64013 / CIP 107961 / Muc) OX=349741 GN=Amuc_0568 PE=3 SV=1	Amuc_0568	2	2	18.90	16.61	143	14.15	1.E+08	6	1.E+07
B2URL1	tr|B2URL1|B2URL1_AKKM8 GCN5-related N-acetyltransferase OS=Akkermansia muciniphila (strain ATCC BAA-835 / DSM 22959 / JCM 33894 / BCRC 81048 / CCUG 64013 / CIP 107961 / Muc) OX=349741 GN=Amuc_1271 PE=4 SV=1	Amuc_1271	1	1	7.70	21.25	181	13.28	1.E+08	13	1.E+07
B2UM07	tr|B2UM07|B2UM07_AKKM8 Carboxyl-terminal protease OS=Akkermansia muciniphila (strain ATCC BAA-835 / DSM 22959 / JCM 33894 / BCRC 81048 / CCUG 64013 / CIP 107961 / Muc) OX=349741 GN=Amuc_1631 PE=3 SV=1	Amuc_1631	8	8	13.60	83.86	748	47.61	4.E+08	31	1.E+07
B2UP66	tr|B2UP66|B2UP66_AKKM8 Dihydroorotase OS=Akkermansia muciniphila (strain ATCC BAA-835 / DSM 22959 / JCM 33894 / BCRC 81048 / CCUG 64013 / CIP 107961 / Muc) OX=349741 GN=pyrC PE=3 SV=1	Amuc_2027	5	5	16.80	36.40	333	11.33	2.E+08	14	1.E+07
B2UN04	tr|B2UN04|B2UN04_AKKM8 Uncharacterized protein OS=Akkermansia muciniphila (strain ATCC BAA-835 / DSM 22959 / JCM 33894 / BCRC 81048 / CCUG 64013 / CIP 107961 / Muc) OX=349741 GN=Amuc_1797 PE=4 SV=1	Amuc_1797	2	2	9.70	33.10	290	6.38	2.E+08	33	1.E+07
B2UR35	tr|B2UR35|B2UR35_AKKM8 ROK family protein OS=Akkermansia muciniphila (strain ATCC BAA-835 / DSM 22959 / JCM 33894 / BCRC 81048 / CCUG 64013 / CIP 107961 / Muc) OX=349741 GN=Amuc_1094 PE=4 SV=1	Amuc_1094	2	2	8.70	35.76	332	12.46	2.E+08	34	1.E+07
B2UMD2	tr|B2UMD2|B2UMD2_AKKM8 Beta-lactamase domain protein OS=Akkermansia muciniphila (strain ATCC BAA-835 / DSM 22959 / JCM 33894 / BCRC 81048 / CCUG 64013 / CIP 107961 / Muc) OX=349741 GN=Amuc_0238 PE=4 SV=1	Amuc_0238	5	5	13.00	52.42	468	14.04	3.E+08	43	1.E+07
B2UL38	tr|B2UL38|B2UL38_AKKM8 GMP kinase OS=Akkermansia muciniphila (strain ATCC BAA-835 / DSM 22959 / JCM 33894 / BCRC 81048 / CCUG 64013 / CIP 107961 / Muc) OX=349741 GN=Amuc_1489 PE=3 SV=1	Amuc_1489	1	1	5.50	22.19	200	6.92	1.E+08	30	1.E+07
B2ULD1	tr|B2ULD1|B2ULD1_AKKM8 PfkB domain protein OS=Akkermansia muciniphila (strain ATCC BAA-835 / DSM 22959 / JCM 33894 / BCRC 81048 / CCUG 64013 / CIP 107961 / Muc) OX=349741 GN=Amuc_0075 PE=4 SV=1	Amuc_0075	3	3	11.80	39.38	355	15.84	2.E+08	7	1.E+07
B2UQN1	tr|B2UQN1|B2UQN1_AKKM8 Adenylate kinase OS=Akkermansia muciniphila (strain ATCC BAA-835 / DSM 22959 / JCM 33894 / BCRC 81048 / CCUG 64013 / CIP 107961 / Muc) OX=349741 GN=adk PE=3 SV=1	Amuc_0934	2	2	12.00	24.69	216	8.73	1.E+08	22	1.E+07
B2UQM3	tr|B2UQM3|B2UQM3_AKKM8 50S ribosomal protein L5 OS=Akkermansia muciniphila (strain ATCC BAA-835 / DSM 22959 / JCM 33894 / BCRC 81048 / CCUG 64013 / CIP 107961 / Muc) OX=349741 GN=rplE PE=3 SV=1	Amuc_0926	1	1	6.20	21.72	193	5.58	1.E+08	9	1.E+07
B2URF3	tr|B2URF3|B2URF3_AKKM8 Uncharacterized protein OS=Akkermansia muciniphila (strain ATCC BAA-835 / DSM 22959 / JCM 33894 / BCRC 81048 / CCUG 64013 / CIP 107961 / Muc) OX=349741 GN=Amuc_1213 PE=4 SV=1	Amuc_1213	2	2	19.70	16.13	147	5.45	7.E+07	2	9.E+06
B2UL75	sp|B2UL75|G1091_AKKM8 Glycosyl hydrolase family 109 protein 1 OS=Akkermansia muciniphila (strain ATCC BAA-835 / DSM 22959 / JCM 33894 / BCRC 81048 / CCUG 64013 / CIP 107961 / Muc) OX=349741 GN=Amuc_0017 PE=3 SV=1	Amuc_0017	4	4	11.40	53.80	481	43.96	2.E+08	14	9.E+06
B2UNI6	tr|B2UNI6|B2UNI6_AKKM8 Uncharacterized protein OS=Akkermansia muciniphila (strain ATCC BAA-835 / DSM 22959 / JCM 33894 / BCRC 81048 / CCUG 64013 / CIP 107961 / Muc) OX=349741 GN=Amuc_0460 PE=4 SV=1	Amuc_0460	1	1	14.50	19.71	173	2.24	7.E+07	5	9.E+06
B2UQ57	tr|B2UQ57|B2UQ57_AKKM8 ADP-L-glycero-D-manno-heptose-6-epimerase OS=Akkermansia muciniphila (strain ATCC BAA-835 / DSM 22959 / JCM 33894 / BCRC 81048 / CCUG 64013 / CIP 107961 / Muc) OX=349741 GN=hldD PE=3 SV=1	Amuc_0756	4	4	16.20	36.86	328	14.77	2.E+08	13	9.E+06

B2UNI9	tr|B2UNI9|B2UNI9_AKKM8 Phosphopantothenoylcysteine decarboxylase OS=Akkermansia muciniphila (strain ATCC BAA-835 / DSM 22959 / JCM 33894 / BCRC 81048 / CCUG 64013 / CIP 107961 / Muc) OX=349741 GN=Amuc_0463 PE=4 SV=1	Amuc_0463	2	2	14.00	19.18	179	7.73	6.E+07	13	9.E+06
B2UNU4	tr|B2UNU4|B2UNU4_AKKM8 Uncharacterized protein OS=Akkermansia muciniphila (strain ATCC BAA-835 / DSM 22959 / JCM 33894 / BCRC 81048 / CCUG 64013 / CIP 107961 / Muc) OX=349741 GN=Amuc_0476 PE=4 SV=1	Amuc_0476	1	1	4.60	33.64	304	13.98	2.E+08	7	9.E+06
B2ULS2	tr|B2ULS2|B2ULS2_AKKM8 Sulfatase OS=Akkermansia muciniphila (strain ATCC BAA-835 / DSM 22959 / JCM 33894 / BCRC 81048 / CCUG 64013 / CIP 107961 / Muc) OX=349741 GN=Amuc_0121 PE=3 SV=1	Amuc_0121	6	6	16.20	57.46	526	22.83	3.E+08	36	9.E+06
B2UN08	tr|B2UN08|B2UN08_AKKM8 Peptidase S15 OS=Akkermansia muciniphila (strain ATCC BAA-835 / DSM 22959 / JCM 33894 / BCRC 81048 / CCUG 64013 / CIP 107961 / Muc) OX=349741 GN=Amuc_1801 PE=4 SV=1	Amuc_1801	5	5	20.10	39.75	359	23.41	2.E+08	18	9.E+06
B2UN18	tr|B2UN18|B2UN18_AKKM8 Uncharacterized protein OS=Akkermansia muciniphila (strain ATCC BAA-835 / DSM 22959 / JCM 33894 / BCRC 81048 / CCUG 64013 / CIP 107961 / Muc) OX=349741 GN=Amuc_1811 PE=4 SV=1	Amuc_1811	3	3	22.00	17.22	150	9.25	8.E+07	15	9.E+06
B2UKV7	tr|B2UKV7|B2UKV7_AKKM8 10 kDa chaperonin OS=Akkermansia muciniphila (strain ATCC BAA-835 / DSM 22959 / JCM 33894 / BCRC 81048 / CCUG 64013 / CIP 107961 / Muc) OX=349741 GN=groS PE=3 SV=1	Amuc_1407	2	2	29.20	10.31	96	7.25	4.E+07	3	8.E+06
B2UQZ5	tr|B2UQZ5|B2UQZ5_AKKM8 Uncharacterized protein OS=Akkermansia muciniphila (strain ATCC BAA-835 / DSM 22959 / JCM 33894 / BCRC 81048 / CCUG 64013 / CIP 107961 / Muc) OX=349741 GN=Amuc_1054 PE=4 SV=1	Amuc_1054	1	1	6.40	27.80	249	13.01	9.E+07	13	8.E+06
B2UQQ5	tr|B2UQQ5|B2UQQ5_AKKM8 Exodeoxyribonuclease III OS=Akkermansia muciniphila (strain ATCC BAA-835 / DSM 22959 / JCM 33894 / BCRC 81048 / CCUG 64013 / CIP 107961 / Muc) OX=349741 GN=Amuc_0958 PE=3 SV=1	Amuc_0958	2	2	7.10	28.58	253	6.92	1.E+08	18	8.E+06
B2UL25	sp|B2UL25|RS6_AKKM8 30S ribosomal protein S6 OS=Akkermansia muciniphila (strain ATCC BAA-835 / DSM 22959 / JCM 33894 / BCRC 81048 / CCUG 64013 / CIP 107961 / Muc) OX=349741 GN=rpsF PE=3 SV=1	Amuc_1476	1	1	19.10	10.64	94	2.72	4.E+07	5	8.E+06
B2UKW1	tr|B2UKW1|B2UKW1_AKKM8 Uncharacterized protein OS=Akkermansia muciniphila (strain ATCC BAA-835 / DSM 22959 / JCM 33894 / BCRC 81048 / CCUG 64013 / CIP 107961 / Muc) OX=349741 GN=Amuc_1411 PE=4 SV=1	Amuc_1411	2	2	16.10	21.91	224	5.25	4.E+07	2	7.E+06
B2UMT3	sp|B2UMT3|RS3_AKKM8 30S ribosomal protein S3 OS=Akkermansia muciniphila (strain ATCC BAA-835 / DSM 22959 / JCM 33894 / BCRC 81048 / CCUG 64013 / CIP 107961 / Muc) OX=349741 GN=rpsC PE=3 SV=1	Amuc_0297	3	3	18.80	25.87	229	19.13	1.E+08	29	7.E+06
B2UQJ8	tr|B2UQJ8|B2UQJ8_AKKM8 Uncharacterized protein OS=Akkermansia muciniphila (strain ATCC BAA-835 / DSM 22959 / JCM 33894 / BCRC 81048 / CCUG 64013 / CIP 107961 / Muc) OX=349741 GN=Amuc_0900 PE=4 SV=1	Amuc_0900	1	1	9.80	16.58	143	3.61	4.E+07	14	7.E+06
B2UN24	tr|B2UN24|B2UN24_AKKM8 dTDP-4-dehydrorhamnose reductase OS=Akkermansia muciniphila (strain ATCC BAA-835 / DSM 22959 / JCM 33894 / BCRC 81048 / CCUG 64013 / CIP 107961 / Muc) OX=349741 GN=Amuc_1817 PE=3 SV=1	Amuc_1817	1	1	6.60	33.39	301	2.95	9.E+07	4	6.E+06
B2UNL1	tr|B2UNL1|B2UNL1_AKKM8 Restriction modification system DNA specificity domain OS=Akkermansia muciniphila (strain ATCC BAA-835 / DSM 22959 / JCM 33894 / BCRC 81048 / CCUG 64013 / CIP 107961 / Muc) OX=349741 GN=Amuc_1914 PE=4 SV=1	Amuc_1914	2	2	9.80	43.90	386	11.72	1.E+08	12	6.E+06
B2UNC1	tr|B2UNC1|B2UNC1_AKKM8 Uncharacterized protein OS=Akkermansia muciniphila (strain ATCC BAA-835 / DSM 22959 / JCM 33894 / BCRC 81048 / CCUG 64013 / CIP 107961 / Muc) OX=349741 GN=Amuc_0394 PE=4 SV=1	Amuc_0394	1	1	6.70	27.22	252	3.45	6.E+07	12	6.E+06
B2URC9	sp|B2URC9|RL28_AKKM8 50S ribosomal protein L28 OS=Akkermansia muciniphila (strain ATCC BAA-835 / DSM 22959 / JCM 33894 / BCRC 81048 / CCUG 64013 / CIP 107961 / Muc) OX=349741 GN=rpmB PE=3 SV=1	Amuc_1189	2	2	23.00	9.85	87	4.41	2.E+07	3	6.E+06
B2UKX6	tr|B2UKX6|B2UKX6_AKKM8 Histidinol-phosphatase OS=Akkermansia muciniphila (strain ATCC BAA-835 / DSM 22959 / JCM 33894 / BCRC 81048 / CCUG 64013 / CIP 107961 / Muc) OX=349741 GN=Amuc_1427 PE=3 SV=1	Amuc_1427	2	2	6.30	30.87	269	2.45	7.E+07	2	5.E+06
B2UQK6	tr|B2UQK6|B2UQK6_AKKM8 3-phosphate/5-hydroxy nucleic acid ligase OS=Akkermansia muciniphila (strain ATCC BAA-835 / DSM 22959 / JCM 33894 / BCRC 81048 / CCUG 64013 / CIP 107961 / Muc) OX=349741 GN=Amuc_0909 PE=3 SV=1	Amuc_0909	4	4	13.70	41.25	371	8.23	1.E+08	10	5.E+06

B2UNM5	tr|B2UNM5|B2UNM5_AKKM8 Periplasmic binding protein OS=Akkermansia muciniphila (strain ATCC BAA-835 / DSM 22959 / JCM 33894 / BCRC 81048 / CCUG 64013 / CIP 107961 / Muc) OX=349741 GN=Amuc_1928 PE=4 SV=1	Amuc_1928	3	3	14.00	32.90	307	6.88	1.E+08	22	5.E+06
B2URM0	tr|B2URM0|B2URM0_AKKM8 Aminopeptidase OS=Akkermansia muciniphila (strain ATCC BAA-835 / DSM 22959 / JCM 33894 / BCRC 81048 / CCUG 64013 / CIP 107961 / Muc) OX=349741 GN=Amuc_1280 PE=4 SV=1	Amuc_1280	2	2	8.40	40.49	369	7.98	9.E+07	5	5.E+06
B2UPU2	tr|B2UPU2|B2UPU2_AKKM8 Sel1 domain protein repeat-containing protein OS=Akkermansia muciniphila (strain ATCC BAA-835 / DSM 22959 / JCM 33894 / BCRC 81048 / CCUG 64013 / CIP 107961 / Muc) OX=349741 GN=Amuc_2163 PE=4 SV=1	Amuc_2163	1	1	1.60	101.34	920	13.11	3.E+08	11	5.E+06
B2UQ85	tr|B2UQ85|B2UQ85_AKKM8 FAD dependent oxidoreductase OS=Akkermansia muciniphila (strain ATCC BAA-835 / DSM 22959 / JCM 33894 / BCRC 81048 / CCUG 64013 / CIP 107961 / Muc) OX=349741 GN=Amuc_0786 PE=4 SV=1	Amuc_0786	2	2	10.10	38.46	346	6.14	9.E+07	17	5.E+06
B2UPK9	tr|B2UPK9|B2UPK9_AKKM8 Transcriptional regulator MraZ OS=Akkermansia muciniphila (strain ATCC BAA-835 / DSM 22959 / JCM 33894 / BCRC 81048 / CCUG 64013 / CIP 107961 / Muc) OX=349741 GN=mraZ PE=3 SV=1	Amuc_0649	3	3	24.50	17.05	151	5.24	5.E+07	9	5.E+06
B2UPI5	tr|B2UPI5|B2UPI5_AKKM8 Exo-alpha-sialidase OS=Akkermansia muciniphila (strain ATCC BAA-835 / DSM 22959 / JCM 33894 / BCRC 81048 / CCUG 64013 / CIP 107961 / Muc) OX=349741 GN=Amuc_0625 PE=4 SV=1	Amuc_0625	2	2	5.70	45.59	419	3.33	1.E+08	6	5.E+06
B2UQH0	tr|B2UQH0|B2UQH0_AKKM8 Acetylornithine and succinylornithine aminotransferase OS=Akkermansia muciniphila (strain ATCC BAA-835 / DSM 22959 / JCM 33894 / BCRC 81048 / CCUG 64013 / CIP 107961 / Muc) OX=349741 GN=Amuc_0872 PE=3 SV=1	Amuc_0872	4	4	12.20	45.36	418	12.23	1.E+08	23	5.E+06
B2URI8	tr|B2URI8|B2URI8_AKKM8 GDP-L-fucose synthase OS=Akkermansia muciniphila (strain ATCC BAA-835 / DSM 22959 / JCM 33894 / BCRC 81048 / CCUG 64013 / CIP 107961 / Muc) OX=349741 GN=fcl PE=3 SV=1	Amuc_1248	3	3	13.50	40.65	362	10.52	7.E+07	12	4.E+06
B2UL95	tr|B2UL95|B2UL95_AKKM8 Amino acid permease-associated region OS=Akkermansia muciniphila (strain ATCC BAA-835 / DSM 22959 / JCM 33894 / BCRC 81048 / CCUG 64013 / CIP 107961 / Muc) OX=349741 GN=Amuc_0037 PE=4 SV=1	Amuc_0037	1	1	3.40	54.19	494	4.05	3.E+07	4	4.E+06
B2URJ3	tr|B2URJ3|B2URJ3_AKKM8 Glutamate synthase (Ferredoxin) OS=Akkermansia muciniphila (strain ATCC BAA-835 / DSM 22959 / JCM 33894 / BCRC 81048 / CCUG 64013 / CIP 107961 / Muc) OX=349741 GN=Amuc_1253 PE=3 SV=1	Amuc_1253	6	6	5.70	161.75	1479	44.35	2.E+08	17	4.E+06
B2URK4	tr|B2URK4|B2URK4_AKKM8 Polyprenyl synthetase OS=Akkermansia muciniphila (strain ATCC BAA-835 / DSM 22959 / JCM 33894 / BCRC 81048 / CCUG 64013 / CIP 107961 / Muc) OX=349741 GN=Amuc_1264 PE=3 SV=1	Amuc_1264	1	1	3.70	32.11	297	4.53	3.E+07	3	3.E+06
B2URE6	tr|B2URE6|B2URE6_AKKM8 Bifunctional purine biosynthesis protein PurH OS=Akkermansia muciniphila (strain ATCC BAA-835 / DSM 22959 / JCM 33894 / BCRC 81048 / CCUG 64013 / CIP 107961 / Muc) OX=349741 GN=purH PE=3 SV=1	Amuc_1206	2	2	4.50	56.36	515	5.65	1.E+08	16	3.E+06
B2UL15	tr|B2UL15|B2UL15_AKKM8 FAD linked oxidase domain protein OS=Akkermansia muciniphila (strain ATCC BAA-835 / DSM 22959 / JCM 33894 / BCRC 81048 / CCUG 64013 / CIP 107961 / Muc) OX=349741 GN=Amuc_1466 PE=4 SV=1	Amuc_1466	4	4	12.90	48.88	458	8.03	8.E+07	10	3.E+06
B2UMH6	tr|B2UMH6|B2UMH6_AKKM8 Histidinol dehydrogenase OS=Akkermansia muciniphila (strain ATCC BAA-835 / DSM 22959 / JCM 33894 / BCRC 81048 / CCUG 64013 / CIP 107961 / Muc) OX=349741 GN=Amuc_1714 PE=3 SV=1	Amuc_1714	2	2	5.30	46.29	432	8.97	6.E+07	7	3.E+06
B2UKT9	sp|B2UKT9|ISPG_AKKM8 4-hydroxy-3-methylbut-2-en-1-yl diphosphate synthase (flavodoxin) OS=Akkermansia muciniphila (strain ATCC BAA-835 / DSM 22959 / JCM 33894 / BCRC 81048 / CCUG 64013 / CIP 107961 / Muc) OX=349741 GN=ispG PE=3 SV=1	Amuc_1388	5	5	13.20	64.49	585	15.11	1.E+08	26	3.E+06
B2ULH3	tr|B2ULH3|B2ULH3_AKKM8 Homoserine O-acetyltransferase OS=Akkermansia muciniphila (strain ATCC BAA-835 / DSM 22959 / JCM 33894 / BCRC 81048 / CCUG 64013 / CIP 107961 / Muc) OX=349741 GN=metXA PE=3 SV=1	Amuc_1539	2	2	7.50	42.86	387	13.92	6.E+07	21	3.E+06
B2UNN7	tr|B2UNN7|B2UNN7_AKKM8 Uncharacterized protein OS=Akkermansia muciniphila (strain ATCC BAA-835 / DSM 22959 / JCM 33894 / BCRC 81048 / CCUG 64013 / CIP 107961 / Muc) OX=349741 GN=Amuc_1940 PE=4 SV=1	Amuc_1940	1	1	7.30	17.21	151	2.86	2.E+07	2	3.E+06

B2UQU9	tr|B2UQU9|B2UQU9_AKKM8 Glycoside hydrolase family 31 OS=Akkermansia muciniphila (strain ATCC BAA-835 / DSM 22959 / JCM 33894 / BCRC 81048 / CCUG 64013 / CIP 107961 / Muc) OX=349741 GN=Amuc_1008 PE=3 SV=1	Amuc_1008	4	4	5.30	146.26	1311	11.39	2.E+08	15	3.E+06
B2UND5	tr|B2UND5|B2UND5_AKKM8 Putative substrate-binding protein of aliphatic sulfonate ABC transporter OS=Akkermansia muciniphila (strain ATCC BAA-835 / DSM 22959 / JCM 33894 / BCRC 81048 / CCUG 64013 / CIP 107961 / Muc) OX=349741 GN=Amuc_0409 PE=4 SV=1	Amuc_0409	2	2	8.30	38.40	348	3.16	5.E+07	13	3.E+06
B2UNJ4	tr|B2UNJ4|B2UNJ4_AKKM8 Histone family protein DNA-binding protein OS=Akkermansia muciniphila (strain ATCC BAA-835 / DSM 22959 / JCM 33894 / BCRC 81048 / CCUG 64013 / CIP 107961 / Muc) OX=349741 GN=Amuc_1896 PE=3 SV=1	Amuc_1896	1	1	11.20	9.46	89	2.66	1.E+07	5	3.E+06
B2UQE7	sp|B2UQE7|MIAB_AKKM8 tRNA-2-methylthio-N(6)-dimethylallyladenosine synthase OS=Akkermansia muciniphila (strain ATCC BAA-835 / DSM 22959 / JCM 33894 / BCRC 81048 / CCUG 64013 / CIP 107961 / Muc) OX=349741 GN=miaB PE=3 SV=1	Amuc_0849	2	2	6.80	51.96	458	6.71	7.E+07	16	3.E+06
B2URA2	tr|B2URA2|B2URA2_AKKM8 Threonine aldolase OS=Akkermansia muciniphila (strain ATCC BAA-835 / DSM 22959 / JCM 33894 / BCRC 81048 / CCUG 64013 / CIP 107961 / Muc) OX=349741 GN=Amuc_1162 PE=4 SV=1	Amuc_1162	1	1	4.60	38.74	345	3.75	5.E+07	0	2.E+06
B2ULC6	tr|B2ULC6|B2ULC6_AKKM8 Tryptophan--tRNA ligase OS=Akkermansia muciniphila (strain ATCC BAA-835 / DSM 22959 / JCM 33894 / BCRC 81048 / CCUG 64013 / CIP 107961 / Muc) OX=349741 GN=trpS PE=3 SV=1	Amuc_0070	2	2	10.00	35.86	321	6.09	4.E+07	3	2.E+06
B2UNV4	tr|B2UNV4|B2UNV4_AKKM8 Ferric uptake regulator, Fur family OS=Akkermansia muciniphila (strain ATCC BAA-835 / DSM 22959 / JCM 33894 / BCRC 81048 / CCUG 64013 / CIP 107961 / Muc) OX=349741 GN=Amuc_0486 PE=4 SV=1	Amuc_0486	1	1	7.50	16.55	146	6.05	2.E+07	3	2.E+06
B2UQX2	tr|B2UQX2|B2UQX2_AKKM8 Cysteine--tRNA ligase OS=Akkermansia muciniphila (strain ATCC BAA-835 / DSM 22959 / JCM 33894 / BCRC 81048 / CCUG 64013 / CIP 107961 / Muc) OX=349741 GN=cysS PE=3 SV=1	Amuc_1031	3	3	7.00	53.02	471	8.35	5.E+07	16	2.E+06
B2UQI0	tr|B2UQI0|B2UQI0_AKKM8 Uncharacterized protein OS=Akkermansia muciniphila (strain ATCC BAA-835 / DSM 22959 / JCM 33894 / BCRC 81048 / CCUG 64013 / CIP 107961 / Muc) OX=349741 GN=Amuc_0882 PE=4 SV=1	Amuc_0882	1	1	11.90	11.35	109	2.99	6.E+06	2	2.E+06
B2UMT7	sp|B2UMT7|RL2_AKKM8 50S ribosomal protein L2 OS=Akkermansia muciniphila (strain ATCC BAA-835 / DSM 22959 / JCM 33894 / BCRC 81048 / CCUG 64013 / CIP 107961 / Muc) OX=349741 GN=rplB PE=3 SV=1	Amuc_0301	1	1	5.40	30.66	278	3.19	2.E+07	4	2.E+06
B2UN50	tr|B2UN50|B2UN50_AKKM8 PDZ/DHR/GLGF domain protein OS=Akkermansia muciniphila (strain ATCC BAA-835 / DSM 22959 / JCM 33894 / BCRC 81048 / CCUG 64013 / CIP 107961 / Muc) OX=349741 GN=Amuc_1844 PE=4 SV=1	Amuc_1844	2	2	10.00	34.08	319	5.79	2.E+07	3	1.E+06
B2ULN0	tr|B2ULN0|B2ULN0_AKKM8 Polyprenyl synthetase OS=Akkermansia muciniphila (strain ATCC BAA-835 / DSM 22959 / JCM 33894 / BCRC 81048 / CCUG 64013 / CIP 107961 / Muc) OX=349741 GN=Amuc_1597 PE=3 SV=1	Amuc_1597	2	2	5.70	37.33	336	2.80	2.E+07	10	1.E+06
B2ULK3	tr|B2ULK3|B2ULK3_AKKM8 Uncharacterized protein OS=Akkermansia muciniphila (strain ATCC BAA-835 / DSM 22959 / JCM 33894 / BCRC 81048 / CCUG 64013 / CIP 107961 / Muc) OX=349741 GN=Amuc_1569 PE=4 SV=1	Amuc_1569	1	1	7.50	18.51	173	5.63	1.E+07	1	1.E+06
B2UM66	tr|B2UM66|B2UM66_AKKM8 2-oxoglutarate dehydrogenase complex component E2 OS=Akkermansia muciniphila (strain ATCC BAA-835 / DSM 22959 / JCM 33894 / BCRC 81048 / CCUG 64013 / CIP 107961 / Muc) OX=349741 GN=Amuc_1692 PE=3 SV=1	Amuc_1692	1	1	5.20	39.16	363	3.09	2.E+07	4	1.E+06
B2UQQ8	tr|B2UQQ8|B2UQQ8_AKKM8 DUF362 domain-containing protein OS=Akkermansia muciniphila (strain ATCC BAA-835 / DSM 22959 / JCM 33894 / BCRC 81048 / CCUG 64013 / CIP 107961 / Muc) OX=349741 GN=Amuc_0961 PE=4 SV=1	Amuc_0961	1	1	5.30	30.33	282	5.62	1.E+07	4	9.E+05
B2UQJ6	tr|B2UQJ6|B2UQJ6_AKKM8 DNA topoisomerase OS=Akkermansia muciniphila (strain ATCC BAA-835 / DSM 22959 / JCM 33894 / BCRC 81048 / CCUG 64013 / CIP 107961 / Muc) OX=349741 GN=Amuc_0898 PE=3 SV=1	Amuc_0898	4	4	4.90	97.19	869	7.12	5.E+07	8	9.E+05
B2ULZ9	sp|B2ULZ9|RIMO_AKKM8 Ribosomal protein S12 methylthiotransferase RimO OS=Akkermansia muciniphila (strain ATCC BAA-835 / DSM 22959 / JCM 33894 / BCRC 81048 / CCUG 64013 / CIP 107961 / Muc) OX=349741 GN=rimO PE=3 SV=1	Amuc_1623	4	4	10.00	50.86	452	5.59	2.E+07	5	9.E+05

B2UQE3	sp|B2UQE3|END4_AKKM8 Probable endonuclease 4 OS=Akkermansia muciniphila (strain ATCC BAA-835 / DSM 22959 / JCM 33894 / BCRC 81048 / CCUG 64013 / CIP 107961 / Muc) OX=349741 GN=nfo PE=3 SV=1	Amuc_0845	3	3	14.10	30.78	277	4.26	1.E+07	1	8.E+05
B2UMT6	sp|B2UMT6|RS19_AKKM8 30S ribosomal protein S19 OS=Akkermansia muciniphila (strain ATCC BAA-835 / DSM 22959 / JCM 33894 / BCRC 81048 / CCUG 64013 / CIP 107961 / Muc) OX=349741 GN=rpsS PE=3 SV=1	Amuc_0300	1	1	16.90	9.80	89	3.40	5.E+06	1	8.E+05
B2UN91	sp|B2UN91|SYE_AKKM8 Glutamate--tRNA ligase OS=Akkermansia muciniphila (strain ATCC BAA-835 / DSM 22959 / JCM 33894 / BCRC 81048 / CCUG 64013 / CIP 107961 / Muc) OX=349741 GN=gltX PE=3 SV=1	Amuc_1888	2	2	7.60	48.61	433	6.33	2.E+07	7	8.E+05
B2UNY2	tr|B2UNY2|B2UNY2_AKKM8 Peptidoglycan glycosyltransferase OS=Akkermansia muciniphila (strain ATCC BAA-835 / DSM 22959 / JCM 33894 / BCRC 81048 / CCUG 64013 / CIP 107961 / Muc) OX=349741 GN=Amuc_0514 PE=4 SV=1	Amuc_0514	2	2	4.10	89.28	822	7.04	3.E+07	3	7.E+05
B2UR84	tr|B2UR84|B2UR84_AKKM8 YD repeat protein OS=Akkermansia muciniphila (strain ATCC BAA-835 / DSM 22959 / JCM 33894 / BCRC 81048 / CCUG 64013 / CIP 107961 / Muc) OX=349741 GN=Amuc_1143 PE=4 SV=1	Amuc_1143	3	3	2.40	217.62	1991	2.39	6.E+07	4	7.E+05
B2ULH4	tr|B2ULH4|B2ULH4_AKKM8 50S ribosomal protein L36 OS=Akkermansia muciniphila (strain ATCC BAA-835 / DSM 22959 / JCM 33894 / BCRC 81048 / CCUG 64013 / CIP 107961 / Muc) OX=349741 GN=rpmJ PE=3 SV=1	Amuc_1540	1	1	6.20	16.69	144	3.70	7.E+06	1	7.E+05
B2UNB9	tr|B2UNB9|B2UNB9_AKKM8 Coagulation factor 5/8 type domain protein OS=Akkermansia muciniphila (strain ATCC BAA-835 / DSM 22959 / JCM 33894 / BCRC 81048 / CCUG 64013 / CIP 107961 / Muc) OX=349741 GN=Amuc_0392 PE=4 SV=1	Amuc_0392	1	1	1.10	78.57	709	2.49	3.E+07	8	6.E+05
B2UQ76	tr|B2UQ76|B2UQ76_AKKM8 Short-chain dehydrogenase/reductase SDR OS=Akkermansia muciniphila (strain ATCC BAA-835 / DSM 22959 / JCM 33894 / BCRC 81048 / CCUG 64013 / CIP 107961 / Muc) OX=349741 GN=Amuc_0777 PE=4 SV=1	Amuc_0777	1	1	4.30	32.30	299	5.96	9.E+06	2	5.E+05
B2UKU3	tr|B2UKU3|B2UKU3_AKKM8 Beta-ketoacyl synthase OS=Akkermansia muciniphila (strain ATCC BAA-835 / DSM 22959 / JCM 33894 / BCRC 81048 / CCUG 64013 / CIP 107961 / Muc) OX=349741 GN=Amuc_1392 PE=3 SV=1	Amuc_1392	2	2	6.40	40.31	390	2.93	4.E+06	1	2.E+05
B2URL5	tr|B2URL5|B2URL5_AKKM8 Uncharacterized protein OS=Akkermansia muciniphila (strain ATCC BAA-835 / DSM 22959 / JCM 33894 / BCRC 81048 / CCUG 64013 / CIP 107961 / Muc) OX=349741 GN=Amuc_1275 PE=4 SV=1	Amuc_1275	2	2	6.60	38.86	348	3.84	2.E+06	2	1.E+05
B2UPQ8	tr|B2UPQ8|B2UPQ8_AKKM8 Carbohydrate-selective porin OprB OS=Akkermansia muciniphila (strain ATCC BAA-835 / DSM 22959 / JCM 33894 / BCRC 81048 / CCUG 64013 / CIP 107961 / Muc) OX=349741 GN=Amuc_2127 PE=3 SV=1	Amuc_2127	1	1	3.00	53.33	494	2.50	0.E+00	2	0.E+00
B2UL46	tr|B2UL46|B2UL46_AKKM8 Uncharacterized protein OS=Akkermansia muciniphila (strain ATCC BAA-835 / DSM 22959 / JCM 33894 / BCRC 81048 / CCUG 64013 / CIP 107961 / Muc) OX=349741 GN=Amuc_1497 PE=4 SV=1	Amuc_1497	2	2	6.60	38.92	349	2.63	0.E+00	2	0.E+00

Supplementary Table 2. List of proteins with at least two unique peptides identified in secretome of *A. muciniphila* cultivated under basal medium.

Majority protein IDs	Fasta headers	Gene locus	Peptides	Unique peptides	Sequence coverage [%]	Mol. weight [kDa]	Sequence lengths	Score	Intensity	MS/MS count	iBAQ
B2UKW7	tr|B2UKW7|B2UKW7_AKKM8 Glyceraldehyde-3-phosphate dehydrogenase OS=Akkermansia muciniphila (strain ATCC BAA-835 / DSM 22959 / JCM 33894 / BCRC 81048 / CCUG 64013 / CIP 107961 / Muc) OX=349741 GN=Amuc_1417 PE=3 SV=1	Amuc_1417	21	21	68.00	36.74	341	323.31	5.E+11	3106	2.E+10
B2UQS2	sp|B2UQS2|ACP_AKKM8 Acyl carrier protein OS=Akkermansia muciniphila (strain ATCC BAA-835 / DSM 22959 / JCM 33894 / BCRC 81048 / CCUG 64013 / CIP 107961 / Muc) OX=349741 GN=acpP PE=3 SV=1	Amuc_0975	3	3	40.00	8.82	80	200.16	6.E+10	1971	2.E+10
B2UP94	tr|B2UP94|B2UP94_AKKM8 Rubrerythrin domain-containing protein OS=Akkermansia muciniphila (strain ATCC BAA-835 / DSM 22959 / JCM 33894 / BCRC 81048 / CCUG 64013 / CIP 107961 / Muc) OX=349741 GN=Amuc_2055 PE=4 SV=1	Amuc_2055	7	5	86.00	12.73	114	264.75	1.E+11	1261	2.E+10
B2UMR7	tr|B2UMR7|B2UMR7_AKKM8 Histone family protein DNA-binding protein OS=Akkermansia muciniphila (strain ATCC BAA-835 / DSM 22959 / JCM 33894 / BCRC 81048 / CCUG 64013 / CIP 107961 / Muc) OX=349741 GN=Amuc_0281 PE=3 SV=1	Amuc_0281	4	4	50.50	11.25	101	210.98	6.E+10	587	2.E+10
B2UQP3	tr|B2UQP3|B2UQP3_AKKM8 DJ-1 family protein OS=Akkermansia muciniphila (strain ATCC BAA-835 / DSM 22959 / JCM 33894 / BCRC 81048 / CCUG 64013 / CIP 107961 / Muc) OX=349741 GN=Amuc_0946 PE=4 SV=1	Amuc_0946	11	11	67.90	20.11	187	323.31	1.E+11	1832	1.E+10
B2ULM5	tr|B2ULM5|B2ULM5_AKKM8 Superoxide dismutase OS=Akkermansia muciniphila (strain ATCC BAA-835 / DSM 22959 / JCM 33894 / BCRC 81048 / CCUG 64013 / CIP 107961 / Muc) OX=349741 GN=Amuc_1592 PE=3 SV=1	Amuc_1592	11	11	56.10	23.92	214	323.31	2.E+11	1249	1.E+10
B2URC4	tr|B2URC4|B2URC4_AKKM8 Enolase OS=Akkermansia muciniphila (strain ATCC BAA-835 / DSM 22959 / JCM 33894 / BCRC 81048 / CCUG 64013 / CIP 107961 / Muc) OX=349741 GN=eno PE=3 SV=1	Amuc_1184	19	17	67.10	45.68	426	323.31	3.E+11	3686	1.E+10
B2UPZ3	tr|B2UPZ3|B2UPZ3_AKKM8 Thioredoxin OS=Akkermansia muciniphila (strain ATCC BAA-835 / DSM 22959 / JCM 33894 / BCRC 81048 / CCUG 64013 / CIP 107961 / Muc) OX=349741 GN=Amuc_0691 PE=3 SV=1	Amuc_0691	5	5	66.70	11.49	105	237.29	6.E+10	502	1.E+10
B2URF0	tr|B2URF0|B2URF0_AKKM8 Phosphoenolpyruvate carboxykinase [GTP] OS=Akkermansia muciniphila (strain ATCC BAA-835 / DSM 22959 / JCM 33894 / BCRC 81048 / CCUG 64013 / CIP 107961 / Muc) OX=349741 GN=pckG PE=3 SV=1	Amuc_1210	22	22	48.40	67.52	608	323.31	2.E+11	2108	8.E+09
B2UN99	tr|B2UN99|B2UN99_AKKM8 Glutamate decarboxylase OS=Akkermansia muciniphila (strain ATCC BAA-835 / DSM 22959 / JCM 33894 / BCRC 81048 / CCUG 64013 / CIP 107961 / Muc) OX=349741 GN=Amuc_0372 PE=3 SV=1	Amuc_0372	24	24	65.90	52.46	466	323.31	2.E+11	2064	7.E+09
B2UKW8	sp|B2UKW8|PGK_AKKM8 Phosphoglycerate kinase OS=Akkermansia muciniphila (strain ATCC BAA-835 / DSM 22959 / JCM 33894 / BCRC 81048 / CCUG 64013 / CIP 107961 / Muc) OX=349741 GN=pgk PE=3 SV=1	Amuc_1418	24	24	75.20	42.69	403	323.31	1.E+11	2595	6.E+09
B2UKV9	tr|B2UKV9|B2UKV9_AKKM8 PepSY_like domain-containing protein OS=Akkermansia muciniphila (strain ATCC BAA-835 / DSM 22959 / JCM 33894 / BCRC 81048 / CCUG 64013 / CIP 107961 / Muc) OX=349741 GN=Amuc_1409 PE=4 SV=1	Amuc_1409	8	8	52.00	16.52	150	245.16	4.E+10	538	5.E+09
B2UQ05	tr|B2UQ05|B2UQ05_AKKM8 Alanine--glyoxylate transaminase OS=Akkermansia muciniphila (strain ATCC BAA-835 / DSM 22959 / JCM 33894 / BCRC 81048 / CCUG 64013 / CIP 107961 / Muc) OX=349741 GN=Amuc_0703 PE=3 SV=1	Amuc_0703	20	20	76.80	39.28	357	323.31	1.E+11	2027	5.E+09
B2UNK0	tr|B2UNK0|B2UNK0_AKKM8 Uncharacterized protein OS=Akkermansia muciniphila (strain ATCC BAA-835 / DSM 22959 / JCM 33894 / BCRC 81048 / CCUG 64013 / CIP 107961 / Muc) OX=349741 GN=Amuc_1902 PE=4 SV=1	Amuc_1902	13	13	71.30	24.93	230	323.31	5.E+10	917	4.E+09
B2UN29	tr|B2UN29|B2UN29_AKKM8 Glucosamine-6-phosphate deaminase OS=Akkermansia muciniphila (strain ATCC BAA-835 / DSM 22959 / JCM 33894 / BCRC 81048 / CCUG 64013 / CIP 107961 / Muc) OX=349741 GN=nagB PE=3 SV=1	Amuc_1822	10	10	46.90	32.84	303	323.31	6.E+10	535	4.E+09
B2UNL9	tr|B2UNL9|B2UNL9_AKKM8 Putative ferredoxin OS=Akkermansia muciniphila (strain ATCC BAA-835 / DSM 22959 / JCM 33894 / BCRC 81048 / CCUG 64013 / CIP 107961 / Muc) OX=349741 GN=Amuc_1922 PE=4 SV=1	Amuc_1922	2	2	24.70	8.40	77	134.67	2.E+10	78	3.E+09

B2UNS8	tr|B2UNS8|B2UNS8_AKKM8 Methylmalonyl-CoA mutase, large subunit OS=Akkermansia muciniphila (strain ATCC BAA-835 / DSM 22959 / JCM 33894 / BCRC 81048 / CCUG 64013 / CIP 107961 / Muc) OX=349741 GN=Amuc_1983 PE=4 SV=1	Amuc_1983	25	25	46.50	78.41	714	323.31	1.E+11	1974	3.E+09
B2UL96	sp|B2UL96|GLSA_AKKM8 Glutaminase OS=Akkermansia muciniphila (strain ATCC BAA-835 / DSM 22959 / JCM 33894 / BCRC 81048 / CCUG 64013 / CIP 107961 / Muc) OX=349741 GN=glsA PE=3 SV=1	Amuc_0038	7	7	31.00	33.13	313	186.41	3.E+10	391	3.E+09
B2UPF0	tr|B2UPF0|B2UPF0_AKKM8 Uncharacterized protein OS=Akkermansia muciniphila (strain ATCC BAA-835 / DSM 22959 / JCM 33894 / BCRC 81048 / CCUG 64013 / CIP 107961 / Muc) OX=349741 GN=Amuc_0590 PE=4 SV=1	Amuc_0590	22	22	51.50	50.45	460	323.31	6.E+10	1665	3.E+09
B2ULM2	tr|B2ULM2|B2ULM2_AKKM8 Aminotransferase OS=Akkermansia muciniphila (strain ATCC BAA-835 / DSM 22959 / JCM 33894 / BCRC 81048 / CCUG 64013 / CIP 107961 / Muc) OX=349741 GN=Amuc_1589 PE=3 SV=1	Amuc_1589	14	14	52.80	42.44	388	323.31	4.E+10	986	3.E+09
B2UNH3	tr|B2UNH3|B2UNH3_AKKM8 Glycine cleavage system H protein OS=Akkermansia muciniphila (strain ATCC BAA-835 / DSM 22959 / JCM 33894 / BCRC 81048 / CCUG 64013 / CIP 107961 / Muc) OX=349741 GN=gcvH PE=3 SV=1	Amuc_0447	3	3	40.50	13.72	126	120.45	5.E+09	111	3.E+09
B2UKY5	sp|B2UKY5|MDH_AKKM8 Malate dehydrogenase OS=Akkermansia muciniphila (strain ATCC BAA-835 / DSM 22959 / JCM 33894 / BCRC 81048 / CCUG 64013 / CIP 107961 / Muc) OX=349741 GN=mdh PE=3 SV=1	Amuc_1436	13	13	47.10	35.10	329	323.31	4.E+10	548	2.E+09
B2UQP5	tr|B2UQP5|B2UQP5_AKKM8 N-acetylglucosamine-6-phosphate deacetylase OS=Akkermansia muciniphila (strain ATCC BAA-835 / DSM 22959 / JCM 33894 / BCRC 81048 / CCUG 64013 / CIP 107961 / Muc) OX=349741 GN=Amuc_0948 PE=3 SV=1	Amuc_0948	12	12	45.30	40.83	382	323.31	4.E+10	614	2.E+09
B2UNS2	tr|B2UNS2|B2UNS2_AKKM8 Glucose-6-phosphate isomerase OS=Akkermansia muciniphila (strain ATCC BAA-835 / DSM 22959 / JCM 33894 / BCRC 81048 / CCUG 64013 / CIP 107961 / Muc) OX=349741 GN=pgi PE=3 SV=1	Amuc_1975	25	25	58.30	55.47	515	323.31	6.E+10	1407	2.E+09
B2UP29	tr|B2UP29|B2UP29_AKKM8 Triosephosphate isomerase OS=Akkermansia muciniphila (strain ATCC BAA-835 / DSM 22959 / JCM 33894 / BCRC 81048 / CCUG 64013 / CIP 107961 / Muc) OX=349741 GN=tpiA PE=3 SV=1	Amuc_0562	10	10	61.40	26.99	254	323.31	2.E+10	517	2.E+09
B2UL55	tr|B2UL55|B2UL55_AKKM8 Malonyl CoA-acyl carrier protein transacylase OS=Akkermansia muciniphila (strain ATCC BAA-835 / DSM 22959 / JCM 33894 / BCRC 81048 / CCUG 64013 / CIP 107961 / Muc) OX=349741 GN=Amuc_1507 PE=3 SV=1	Amuc_1507	9	9	41.90	32.57	308	323.31	3.E+10	444	2.E+09
B2URK9	tr|B2URK9|B2URK9_AKKM8 Thioredoxin reductase OS=Akkermansia muciniphila (strain ATCC BAA-835 / DSM 22959 / JCM 33894 / BCRC 81048 / CCUG 64013 / CIP 107961 / Muc) OX=349741 GN=Amuc_1269 PE=3 SV=1	Amuc_1269	8	8	31.70	33.21	309	248.71	2.E+10	506	2.E+09
B2UPR8	tr|B2UPR8|B2UPR8_AKKM8 Nitroreductase OS=Akkermansia muciniphila (strain ATCC BAA-835 / DSM 22959 / JCM 33894 / BCRC 81048 / CCUG 64013 / CIP 107961 / Muc) OX=349741 GN=Amuc_2137 PE=3 SV=1	Amuc_2137	11	11	49.30	24.41	215	275.58	2.E+10	767	2.E+09
B2ULH2	tr|B2ULH2|B2ULH2_AKKM8 OsmC family protein OS=Akkermansia muciniphila (strain ATCC BAA-835 / DSM 22959 / JCM 33894 / BCRC 81048 / CCUG 64013 / CIP 107961 / Muc) OX=349741 GN=Amuc_1538 PE=4 SV=1	Amuc_1538	4	4	49.30	14.99	136	173.93	9.E+09	241	1.E+09
B2UR48	tr|B2UR48|B2UR48_AKKM8 ATP phosphoribosyltransferase OS=Akkermansia muciniphila (strain ATCC BAA-835 / DSM 22959 / JCM 33894 / BCRC 81048 / CCUG 64013 / CIP 107961 / Muc) OX=349741 GN=hisG PE=3 SV=1	Amuc_1107	11	11	52.20	32.70	291	323.31	2.E+10	397	1.E+09
B2UP53	tr|B2UP53|B2UP53_AKKM8 Cysteine synthase OS=Akkermansia muciniphila (strain ATCC BAA-835 / DSM 22959 / JCM 33894 / BCRC 81048 / CCUG 64013 / CIP 107961 / Muc) OX=349741 GN=Amuc_2014 PE=3 SV=1	Amuc_2014	15	15	86.40	32.53	309	323.31	2.E+10	867	1.E+09
B2UPE1	tr|B2UPE1|B2UPE1_AKKM8 Meso-diaminopimelate D-dehydrogenase OS=Akkermansia muciniphila (strain ATCC BAA-835 / DSM 22959 / JCM 33894 / BCRC 81048 / CCUG 64013 / CIP 107961 / Muc) OX=349741 GN=Amuc_0581 PE=3 SV=1	Amuc_0581	17	17	72.40	31.31	293	323.31	2.E+10	1032	1.E+09
B2URE7	sp|B2URE7|PDXJ_AKKM8 Pyridoxine 5-phosphate synthase OS=Akkermansia muciniphila (strain ATCC BAA-835 / DSM 22959 / JCM 33894 / BCRC 81048 / CCUG 64013 / CIP 107961 / Muc) OX=349741 GN=pdxJ PE=3 SV=1	Amuc_1207	7	7	49.60	27.11	246	323.31	1.E+10	1276	1.E+09

B2UL89	tr|B2UL89|B2UL89_AKKM8 NTP_transferase domain-containing protein OS=Akkermansia muciniphila (strain ATCC BAA-835 / DSM 22959 / JCM 33894 / BCRC 81048 / CCUG 64013 / CIP 107961 / Muc) OX=349741 GN=Amuc_0031 PE=4 SV=1	Amuc_0031	10	10	46.70	32.12	291	164.51	2.E+10	381	1.E+09
B2UMF1	sp|B2UMF1|DAPB_AKKM8 4-hydroxy-tetrahydrodipicolinate reductase OS=Akkermansia muciniphila (strain ATCC BAA-835 / DSM 22959 / JCM 33894 / BCRC 81048 / CCUG 64013 / CIP 107961 / Muc) OX=349741 GN=dapB PE=3 SV=1	Amuc_0257	8	8	50.00	26.31	246	289.43	2.E+10	443	1.E+09
B2UMG7	tr|B2UMG7|B2UMG7_AKKM8 Endoribonuclease L-PSP OS=Akkermansia muciniphila (strain ATCC BAA-835 / DSM 22959 / JCM 33894 / BCRC 81048 / CCUG 64013 / CIP 107961 / Muc) OX=349741 GN=Amuc_1705 PE=4 SV=1	Amuc_1705	6	6	67.90	16.64	156	323.31	8.E+09	590	1.E+09
B2UNK1	tr|B2UNK1|B2UNK1_AKKM8 Uncharacterized protein OS=Akkermansia muciniphila (strain ATCC BAA-835 / DSM 22959 / JCM 33894 / BCRC 81048 / CCUG 64013 / CIP 107961 / Muc) OX=349741 GN=Amuc_1903 PE=4 SV=1	Amuc_1903	10	10	40.30	35.41	335	323.31	2.E+10	554	1.E+09
B2UM96	tr|B2UM96|B2UM96_AKKM8 Carboxyl transferase OS=Akkermansia muciniphila (strain ATCC BAA-835 / DSM 22959 / JCM 33894 / BCRC 81048 / CCUG 64013 / CIP 107961 / Muc) OX=349741 GN=Amuc_0201 PE=4 SV=1	Amuc_0201	11	11	33.60	56.01	521	323.31	2.E+10	506	1.E+09
B2ULV5	tr|B2ULV5|B2ULV5_AKKM8 Phosphoglucomutase/phosphomannomutase alpha/beta/alpha domain I OS=Akkermansia muciniphila (strain ATCC BAA-835 / DSM 22959 / JCM 33894 / BCRC 81048 / CCUG 64013 / CIP 107961 / Muc) OX=349741 GN=Amuc_0155 PE=3 SV=1	Amuc_0155	20	20	42.70	71.24	651	323.31	3.E+10	1126	1.E+09
B2UP27	tr|B2UP27|B2UP27_AKKM8 Argininosuccinate synthase OS=Akkermansia muciniphila (strain ATCC BAA-835 / DSM 22959 / JCM 33894 / BCRC 81048 / CCUG 64013 / CIP 107961 / Muc) OX=349741 GN=argG PE=3 SV=1	Amuc_0559	14	14	44.70	47.87	430	308.42	2.E+10	1173	1.E+09
B2UQY9	sp|B2UQY9|EFTU_AKKM8 Elongation factor Tu OS=Akkermansia muciniphila (strain ATCC BAA-835 / DSM 22959 / JCM 33894 / BCRC 81048 / CCUG 64013 / CIP 107961 / Muc) OX=349741 GN=tuf PE=3 SV=1	Amuc_1048	10	10	34.30	43.43	394	323.31	2.E+10	628	1.E+09
B2UMA1	tr|B2UMA1|B2UMA1_AKKM8 Succinate CoA transferase OS=Akkermansia muciniphila (strain ATCC BAA-835 / DSM 22959 / JCM 33894 / BCRC 81048 / CCUG 64013 / CIP 107961 / Muc) OX=349741 GN=Amuc_0206 PE=3 SV=1	Amuc_0206	23	23	61.00	54.05	498	323.31	2.E+10	859	9.E+08
B2UKM8	tr|B2UKM8|B2UKM8_AKKM8 3-oxoacyl-[acyl-carrier-protein] synthase 2 OS=Akkermansia muciniphila (strain ATCC BAA-835 / DSM 22959 / JCM 33894 / BCRC 81048 / CCUG 64013 / CIP 107961 / Muc) OX=349741 GN=Amuc_1327 PE=3 SV=1	Amuc_1327	8	8	34.70	43.82	415	251.36	1.E+10	885	9.E+08
B2UMF2	tr|B2UMF2|B2UMF2_AKKM8 4-hydroxy-tetrahydrodipicolinate synthase OS=Akkermansia muciniphila (strain ATCC BAA-835 / DSM 22959 / JCM 33894 / BCRC 81048 / CCUG 64013 / CIP 107961 / Muc) OX=349741 GN=dapA PE=3 SV=1	Amuc_0258	7	7	34.10	31.25	293	205.55	2.E+10	458	9.E+08
B2UNS9	tr|B2UNS9|B2UNS9_AKKM8 Methylmalonyl-CoA mutase OS=Akkermansia muciniphila (strain ATCC BAA-835 / DSM 22959 / JCM 33894 / BCRC 81048 / CCUG 64013 / CIP 107961 / Muc) OX=349741 GN=Amuc_1984 PE=4 SV=1	Amuc_1984	26	26	50.60	74.10	684	323.31	3.E+10	1054	9.E+08
B2UPB0	tr|B2UPB0|B2UPB0_AKKM8 Rubrerythrin OS=Akkermansia muciniphila (strain ATCC BAA-835 / DSM 22959 / JCM 33894 / BCRC 81048 / CCUG 64013 / CIP 107961 / Muc) OX=349741 GN=Amuc_2072 PE=4 SV=1	Amuc_2072	3	3	19.90	21.34	191	177.17	6.E+09	143	8.E+08
B2UQW7	tr|B2UQW7|B2UQW7_AKKM8 Peptidyl-prolyl cis-trans isomerase OS=Akkermansia muciniphila (strain ATCC BAA-835 / DSM 22959 / JCM 33894 / BCRC 81048 / CCUG 64013 / CIP 107961 / Muc) OX=349741 GN=Amuc_1026 PE=3 SV=1	Amuc_1026	5	5	47.90	17.92	167	260.88	6.E+09	192	8.E+08
B2UQ07	tr|B2UQ07|B2UQ07_AKKM8 2-oxoglutarate reductase OS=Akkermansia muciniphila (strain ATCC BAA-835 / DSM 22959 / JCM 33894 / BCRC 81048 / CCUG 64013 / CIP 107961 / Muc) OX=349741 GN=Amuc_0705 PE=3 SV=1	Amuc_0705	14	14	37.90	56.68	523	323.31	2.E+10	561	8.E+08
B2UMX5	tr|B2UMX5|B2UMX5_AKKM8 OsmC family protein OS=Akkermansia muciniphila (strain ATCC BAA-835 / DSM 22959 / JCM 33894 / BCRC 81048 / CCUG 64013 / CIP 107961 / Muc) OX=349741 GN=Amuc_0342 PE=4 SV=1	Amuc_0342	7	7	65.20	14.48	135	113.32	5.E+09	262	8.E+08
B2UNP8	tr|B2UNP8|B2UNP8_AKKM8 NADH:flavin oxidoreductase/NADH oxidase OS=Akkermansia muciniphila (strain ATCC BAA-835 / DSM 22959 / JCM 33894 / BCRC 81048 / CCUG 64013 / CIP 107961 / Muc) OX=349741 GN=Amuc_1951 PE=4 SV=1	Amuc_1951	16	16	49.90	40.82	367	323.31	1.E+10	578	7.E+08

B2UPU6	tr|B2UPU6|B2UPU6_AKKM8 Oligopeptidase A OS=Akkermansia muciniphila (strain ATCC BAA-835 / DSM 22959 / JCM 33894 / BCRC 81048 / CCUG 64013 / CIP 107961 / Muc) OX=349741 GN=Amuc_2167 PE=3 SV=1	Amuc_2167	18	18	38.40	79.32	700	323.31	3.E+10	871	7.E+08
B2URP8	tr|B2URP8|B2URP8_AKKM8 Aldose 1-epimerase OS=Akkermansia muciniphila (strain ATCC BAA-835 / DSM 22959 / JCM 33894 / BCRC 81048 / CCUG 64013 / CIP 107961 / Muc) OX=349741 GN=Amuc_1309 PE=3 SV=1	Amuc_1309	11	11	49.20	34.50	319	287.90	1.E+10	405	7.E+08
B2UL16	tr|B2UL16|B2UL16_AKKM8 Uncharacterized protein OS=Akkermansia muciniphila (strain ATCC BAA-835 / DSM 22959 / JCM 33894 / BCRC 81048 / CCUG 64013 / CIP 107961 / Muc) OX=349741 GN=Amuc_1467 PE=4 SV=1	Amuc_1467	4	4	16.50	22.00	194	98.66	6.E+09	160	7.E+08
B2UN39	tr|B2UN39|B2UN39_AKKM8 L-fucose isomerase OS=Akkermansia muciniphila (strain ATCC BAA-835 / DSM 22959 / JCM 33894 / BCRC 81048 / CCUG 64013 / CIP 107961 / Muc) OX=349741 GN=fucI PE=3 SV=1	Amuc_1832	20	20	39.20	64.70	592	323.31	2.E+10	765	7.E+08
B2UML6	sp|B2UML6|OTC_AKKM8 Ornithine carbamoyltransferase OS=Akkermansia muciniphila (strain ATCC BAA-835 / DSM 22959 / JCM 33894 / BCRC 81048 / CCUG 64013 / CIP 107961 / Muc) OX=349741 GN=arcB PE=3 SV=1	Amuc_1630	11	11	47.90	34.14	305	323.31	1.E+10	527	7.E+08
B2UMU5	sp|B2UMU5|GPMI_AKKM8 2,3-bisphosphoglycerate-independent phosphoglycerate mutase OS=Akkermansia muciniphila (strain ATCC BAA-835 / DSM 22959 / JCM 33894 / BCRC 81048 / CCUG 64013 / CIP 107961 / Muc) OX=349741 GN=gpmI PE=3 SV=1	Amuc_0309	14	14	37.70	56.14	514	323.31	2.E+10	554	7.E+08
B2UPJ1	tr|B2UPJ1|B2UPJ1_AKKM8 FAD-binding domain protein OS=Akkermansia muciniphila (strain ATCC BAA-835 / DSM 22959 / JCM 33894 / BCRC 81048 / CCUG 64013 / CIP 107961 / Muc) OX=349741 GN=Amuc_0631 PE=4 SV=1	Amuc_0631	13	13	53.50	41.44	376	323.31	1.E+10	521	7.E+08
B2UNK9	tr|B2UNK9|B2UNK9_AKKM8 Iron-containing alcohol dehydrogenase OS=Akkermansia muciniphila (strain ATCC BAA-835 / DSM 22959 / JCM 33894 / BCRC 81048 / CCUG 64013 / CIP 107961 / Muc) OX=349741 GN=Amuc_1911 PE=4 SV=1	Amuc_1911	9	9	37.60	41.35	386	175.54	9.E+09	482	7.E+08
B2UMC9	tr|B2UMC9|B2UMC9_AKKM8 Alpha-glucan phosphorylase OS=Akkermansia muciniphila (strain ATCC BAA-835 / DSM 22959 / JCM 33894 / BCRC 81048 / CCUG 64013 / CIP 107961 / Muc) OX=349741 GN=Amuc_0235 PE=3 SV=1	Amuc_0235	22	22	49.50	63.41	554	323.31	2.E+10	936	6.E+08
B2UPQ6	tr|B2UPQ6|B2UPQ6_AKKM8 Adenylosuccinate synthetase OS=Akkermansia muciniphila (strain ATCC BAA-835 / DSM 22959 / JCM 33894 / BCRC 81048 / CCUG 64013 / CIP 107961 / Muc) OX=349741 GN=purA PE=3 SV=1	Amuc_2125	10	10	28.80	46.05	423	219.74	1.E+10	788	6.E+08
B2UL87	tr|B2UL87|B2UL87_AKKM8 NAD-dependent epimerase/dehydratase OS=Akkermansia muciniphila (strain ATCC BAA-835 / DSM 22959 / JCM 33894 / BCRC 81048 / CCUG 64013 / CIP 107961 / Muc) OX=349741 GN=Amuc_0029 PE=4 SV=1	Amuc_0029	10	10	39.30	33.59	308	165.07	1.E+10	440	6.E+08
B2UP16	tr|B2UP16|B2UP16_AKKM8 MotA/TolQ/ExbB proton channel OS=Akkermansia muciniphila (strain ATCC BAA-835 / DSM 22959 / JCM 33894 / BCRC 81048 / CCUG 64013 / CIP 107961 / Muc) OX=349741 GN=Amuc_0548 PE=3 SV=1	Amuc_0548	2	2	11.80	28.34	263	12.58	3.E+09	44	6.E+08
B2UNS1	tr|B2UNS1|B2UNS1_AKKM8 Pyruvate, phosphate dikinase OS=Akkermansia muciniphila (strain ATCC BAA-835 / DSM 22959 / JCM 33894 / BCRC 81048 / CCUG 64013 / CIP 107961 / Muc) OX=349741 GN=Amuc_1974 PE=3 SV=1	Amuc_1974	22	22	30.60	100.12	914	323.31	3.E+10	1239	6.E+08
B2UKY9	tr|B2UKY9|B2UKY9_AKKM8 Nitroreductase OS=Akkermansia muciniphila (strain ATCC BAA-835 / DSM 22959 / JCM 33894 / BCRC 81048 / CCUG 64013 / CIP 107961 / Muc) OX=349741 GN=Amuc_1440 PE=3 SV=1	Amuc_1440	7	7	31.60	24.93	215	58.67	5.E+09	169	6.E+08
B2UQR6	tr|B2UQR6|B2UQR6_AKKM8 Galactokinase OS=Akkermansia muciniphila (strain ATCC BAA-835 / DSM 22959 / JCM 33894 / BCRC 81048 / CCUG 64013 / CIP 107961 / Muc) OX=349741 GN=Amuc_0969 PE=3 SV=1	Amuc_0969	9	9	29.30	42.15	392	223.84	1.E+10	418	5.E+08
B2UN63	tr|B2UN63|B2UN63_AKKM8 Formate--tetrahydrofolate ligase OS=Akkermansia muciniphila (strain ATCC BAA-835 / DSM 22959 / JCM 33894 / BCRC 81048 / CCUG 64013 / CIP 107961 / Muc) OX=349741 GN=fhs PE=3 SV=1	Amuc_1860	17	17	40.90	59.80	557	323.31	2.E+10	898	5.E+08
B2UMC7	tr|B2UMC7|B2UMC7_AKKM8 L-threonine 3-dehydrogenase OS=Akkermansia muciniphila (strain ATCC BAA-835 / DSM 22959 / JCM 33894 / BCRC 81048 / CCUG 64013 / CIP 107961 / Muc) OX=349741 GN=tdh PE=3 SV=1	Amuc_0233	7	7	31.00	37.54	345	250.40	7.E+09	294	5.E+08

B2UM06	tr|B2UM06|B2UM06_AKKM8 Serine hydroxymethyltransferase OS=Akkermansia muciniphila (strain ATCC BAA-835 / DSM 22959 / JCM 33894 / BCRC 81048 / CCUG 64013 / CIP 107961 / Muc) OX=349741 GN=glyA PE=3 SV=1	Amuc_1630	22	22	50.40	61.10	566	323.31	2.E+10	849	5.E+08
B2UP04	tr|B2UP04|B2UP04_AKKM8 Uncharacterized protein OS=Akkermansia muciniphila (strain ATCC BAA-835 / DSM 22959 / JCM 33894 / BCRC 81048 / CCUG 64013 / CIP 107961 / Muc) OX=349741 GN=Amuc_0536 PE=4 SV=1	Amuc_0536	6	6	34.00	28.70	259	195.84	6.E+09	190	5.E+08
B2UMA5	tr|B2UMA5|B2UMA5_AKKM8 6-phosphofructokinase OS=Akkermansia muciniphila (strain ATCC BAA-835 / DSM 22959 / JCM 33894 / BCRC 81048 / CCUG 64013 / CIP 107961 / Muc) OX=349741 GN=Amuc_0210 PE=4 SV=1	Amuc_0210	7	7	25.00	35.31	320	144.82	6.E+09	251	5.E+08
B2UQW6	tr|B2UQW6|B2UQW6_AKKM8 Disulfide bond chaperones of the HSP33 family-like protein OS=Akkermansia muciniphila (strain ATCC BAA-835 / DSM 22959 / JCM 33894 / BCRC 81048 / CCUG 64013 / CIP 107961 / Muc) OX=349741 GN=Amuc_1025 PE=4 SV=1	Amuc_1025	10	10	47.10	30.09	257	310.77	7.E+09	575	5.E+08
B2UNP3	tr|B2UNP3|B2UNP3_AKKM8 Dihydrodipicolinate synthetase OS=Akkermansia muciniphila (strain ATCC BAA-835 / DSM 22959 / JCM 33894 / BCRC 81048 / CCUG 64013 / CIP 107961 / Muc) OX=349741 GN=Amuc_1946 PE=3 SV=1	Amuc_1946	6	6	25.60	33.03	305	119.27	8.E+09	463	5.E+08
B2UPS4	tr|B2UPS4|B2UPS4_AKKM8 Fumarate hydratase class I OS=Akkermansia muciniphila (strain ATCC BAA-835 / DSM 22959 / JCM 33894 / BCRC 81048 / CCUG 64013 / CIP 107961 / Muc) OX=349741 GN=Amuc_2144 PE=3 SV=1	Amuc_2144	21	21	53.80	60.10	548	323.31	2.E+10	611	5.E+08
B2UN19	tr|B2UN19|B2UN19_AKKM8 Alpha amylase catalytic region OS=Akkermansia muciniphila (strain ATCC BAA-835 / DSM 22959 / JCM 33894 / BCRC 81048 / CCUG 64013 / CIP 107961 / Muc) OX=349741 GN=Amuc_1812 PE=3 SV=1	Amuc_1812	12	12	37.00	56.72	492	162.51	1.E+10	509	4.E+08
B2UQK1	tr|B2UQK1|B2UQK1_AKKM8 Aconitate hydratase OS=Akkermansia muciniphila (strain ATCC BAA-835 / DSM 22959 / JCM 33894 / BCRC 81048 / CCUG 64013 / CIP 107961 / Muc) OX=349741 GN=Amuc_0904 PE=3 SV=1	Amuc_0904	23	23	38.40	99.01	917	323.31	2.E+10	1077	4.E+08
B2URI9	tr|B2URI9|B2URI9_AKKM8 GDP-mannose 4,6-dehydratase OS=Akkermansia muciniphila (strain ATCC BAA-835 / DSM 22959 / JCM 33894 / BCRC 81048 / CCUG 64013 / CIP 107961 / Muc) OX=349741 GN=gmd PE=3 SV=1	Amuc_1249	14	14	51.00	40.86	355	219.04	9.E+09	591	4.E+08
B2UQE5	tr|B2UQE5|B2UQE5_AKKM8 Metal dependent phosphohydrolase OS=Akkermansia muciniphila (strain ATCC BAA-835 / DSM 22959 / JCM 33894 / BCRC 81048 / CCUG 64013 / CIP 107961 / Muc) OX=349741 GN=Amuc_0847 PE=4 SV=1	Amuc_0847	9	9	33.90	39.25	354	58.34	7.E+09	76	4.E+08
B2UMK3	tr|B2UMK3|B2UMK3_AKKM8 UTP--glucose-1-phosphate uridylyltransferase OS=Akkermansia muciniphila (strain ATCC BAA-835 / DSM 22959 / JCM 33894 / BCRC 81048 / CCUG 64013 / CIP 107961 / Muc) OX=349741 GN=Amuc_1741 PE=3 SV=1	Amuc_1741	15	15	42.50	50.44	461	291.62	9.E+09	394	4.E+08
B2UN83	tr|B2UN83|B2UN83_AKKM8 Isocitrate dehydrogenase [NADP] OS=Akkermansia muciniphila (strain ATCC BAA-835 / DSM 22959 / JCM 33894 / BCRC 81048 / CCUG 64013 / CIP 107961 / Muc) OX=349741 GN=Amuc_1880 PE=4 SV=1	Amuc_1880	18	18	50.10	45.31	415	166.12	9.E+09	552	4.E+08
B2UQ06	tr|B2UQ06|B2UQ06_AKKM8 Uncharacterized protein OS=Akkermansia muciniphila (strain ATCC BAA-835 / DSM 22959 / JCM 33894 / BCRC 81048 / CCUG 64013 / CIP 107961 / Muc) OX=349741 GN=Amuc_0704 PE=4 SV=1	Amuc_0704	9	9	26.40	47.48	417	313.47	8.E+09	349	4.E+08
B2UMM1	tr|B2UMM1|B2UMM1_AKKM8 Peptidase M20 OS=Akkermansia muciniphila (strain ATCC BAA-835 / DSM 22959 / JCM 33894 / BCRC 81048 / CCUG 64013 / CIP 107961 / Muc) OX=349741 GN=Amuc_1759 PE=4 SV=1	Amuc_1759	11	11	24.30	50.33	465	139.33	7.E+09	496	3.E+08
B2UR66	tr|B2UR66|B2UR66_AKKM8 UDP-glucose 4-epimerase OS=Akkermansia muciniphila (strain ATCC BAA-835 / DSM 22959 / JCM 33894 / BCRC 81048 / CCUG 64013 / CIP 107961 / Muc) OX=349741 GN=Amuc_1125 PE=3 SV=1	Amuc_1125	7	7	31.30	36.07	329	100.94	5.E+09	310	3.E+08
B2UKM2	tr|B2UKM2|B2UKM2_AKKM8 Alkyl hydroperoxide reductase/ Thiol specific antioxidant/ Mal allergen OS=Akkermansia muciniphila (strain ATCC BAA-835 / DSM 22959 / JCM 33894 / BCRC 81048 / CCUG 64013 / CIP 107961 / Muc) OX=349741 GN=Amuc_1321 PE=3 SV=1	Amuc_1321	5	5	36.20	22.83	207	126.45	3.E+09	170	3.E+08
B2UN36	tr|B2UN36|B2UN36_AKKM8 Class II aldolase/adducin family protein OS=Akkermansia muciniphila (strain ATCC BAA-835 / DSM 22959 / JCM 33894 / BCRC 81048 / CCUG 64013 / CIP 107961 / Muc) OX=349741 GN=Amuc_1829 PE=4 SV=1	Amuc_1829	7	7	39.90	29.81	271	200.78	4.E+09	313	3.E+08

B2UKP3	tr|B2UKP3|B2UKP3_AKKM8 Uncharacterized protein OS=Akkermansia muciniphila (strain ATCC BAA-835 / DSM 22959 / JCM 33894 / BCRC 81048 / CCUG 64013 / CIP 107961 / Muc) OX=349741 GN=Amuc_1342 PE=4 SV=1	Amuc_1342	4	4	17.30	34.98	312	90.38	5.E+09	235	3.E+08
B2UMG1	tr|B2UMG1|B2UMG1_AKKM8 Uncharacterized protein OS=Akkermansia muciniphila (strain ATCC BAA-835 / DSM 22959 / JCM 33894 / BCRC 81048 / CCUG 64013 / CIP 107961 / Muc) OX=349741 GN=Amuc_0267 PE=4 SV=1	Amuc_0267	5	5	30.10	18.11	163	118.25	3.E+09	255	3.E+08
B2UR47	tr|B2UR47|B2UR47_AKKM8 Peptidase M24 OS=Akkermansia muciniphila (strain ATCC BAA-835 / DSM 22959 / JCM 33894 / BCRC 81048 / CCUG 64013 / CIP 107961 / Muc) OX=349741 GN=Amuc_1106 PE=4 SV=1	Amuc_1106	11	11	26.00	48.19	427	134.55	6.E+09	465	3.E+08
B2ULA2	tr|B2ULA2|B2ULA2_AKKM8 Diaminopimelate epimerase OS=Akkermansia muciniphila (strain ATCC BAA-835 / DSM 22959 / JCM 33894 / BCRC 81048 / CCUG 64013 / CIP 107961 / Muc) OX=349741 GN=dapF PE=3 SV=1	Amuc_0044	6	6	28.50	29.30	274	140.20	4.E+09	193	3.E+08
B2UL47	tr|B2UL47|B2UL47_AKKM8 Uncharacterized protein OS=Akkermansia muciniphila (strain ATCC BAA-835 / DSM 22959 / JCM 33894 / BCRC 81048 / CCUG 64013 / CIP 107961 / Muc) OX=349741 GN=Amuc_1498 PE=3 SV=1	Amuc_1498	8	8	43.20	25.20	229	106.89	3.E+09	238	3.E+08
B2UPF7	tr|B2UPF7|B2UPF7_AKKM8 Flavin_Reduct domain-containing protein OS=Akkermansia muciniphila (strain ATCC BAA-835 / DSM 22959 / JCM 33894 / BCRC 81048 / CCUG 64013 / CIP 107961 / Muc) OX=349741 GN=Amuc_0597 PE=4 SV=1	Amuc_0597	4	4	30.40	19.05	168	60.60	2.E+09	220	3.E+08
B2UQT1	tr|B2UQT1|B2UQT1_AKKM8 Uncharacterized protein OS=Akkermansia muciniphila (strain ATCC BAA-835 / DSM 22959 / JCM 33894 / BCRC 81048 / CCUG 64013 / CIP 107961 / Muc) OX=349741 GN=Amuc_0987 PE=4 SV=1	Amuc_0987	5	5	67.80	9.56	90	65.07	2.E+09	167	3.E+08
B2URF8	tr|B2URF8|B2URF8_AKKM8 3-dehydroquinate synthase OS=Akkermansia muciniphila (strain ATCC BAA-835 / DSM 22959 / JCM 33894 / BCRC 81048 / CCUG 64013 / CIP 107961 / Muc) OX=349741 GN=aroB PE=3 SV=1	Amuc_1218	11	11	44.10	39.26	370	178.11	6.E+09	214	3.E+08
B2UR44	tr|B2UR44|B2UR44_AKKM8 Glutamyl aminopeptidase OS=Akkermansia muciniphila (strain ATCC BAA-835 / DSM 22959 / JCM 33894 / BCRC 81048 / CCUG 64013 / CIP 107961 / Muc) OX=349741 GN=Amuc_1103 PE=3 SV=1	Amuc_1103	8	8	29.50	38.13	359	70.29	5.E+09	169	3.E+08
B2UQ25	tr|B2UQ25|B2UQ25_AKKM8 Branched-chain-amino-acid aminotransferase OS=Akkermansia muciniphila (strain ATCC BAA-835 / DSM 22959 / JCM 33894 / BCRC 81048 / CCUG 64013 / CIP 107961 / Muc) OX=349741 GN=ilvE PE=3 SV=1	Amuc_0723	6	6	31.90	31.84	288	120.56	5.E+09	154	3.E+08
B2UKZ6	sp|B2UKZ6|ARGC_AKKM8 N-acetyl-gamma-glutamyl-phosphate reductase OS=Akkermansia muciniphila (strain ATCC BAA-835 / DSM 22959 / JCM 33894 / BCRC 81048 / CCUG 64013 / CIP 107961 / Muc) OX=349741 GN=argC PE=3 SV=1	Amuc_1447	7	7	34.60	37.59	347	137.05	4.E+09	246	3.E+08
B2UPN7	tr|B2UPN7|B2UPN7_AKKM8 Malate dehydrogenase (Oxaloacetate-decarboxylating) (NADP(+)) OS=Akkermansia muciniphila (strain ATCC BAA-835 / DSM 22959 / JCM 33894 / BCRC 81048 / CCUG 64013 / CIP 107961 / Muc) OX=349741 GN=Amuc_2106 PE=3 SV=1	Amuc_2106	12	12	45.00	47.52	436	166.38	6.E+09	271	3.E+08
B2UMD1	tr|B2UMD1|B2UMD1_AKKM8 Arsenate reductase and related OS=Akkermansia muciniphila (strain ATCC BAA-835 / DSM 22959 / JCM 33894 / BCRC 81048 / CCUG 64013 / CIP 107961 / Muc) OX=349741 GN=Amuc_0237 PE=3 SV=1	Amuc_0237	5	5	46.20	13.52	119	55.32	2.E+09	110	3.E+08
B2URB8	sp|B2URB8|ILVC_AKKM8 Ketol-acid reductoisomerase (NADP(+)) OS=Akkermansia muciniphila (strain ATCC BAA-835 / DSM 22959 / JCM 33894 / BCRC 81048 / CCUG 64013 / CIP 107961 / Muc) OX=349741 GN=ilvC PE=3 SV=1	Amuc_1178	10	10	44.80	35.14	326	175.89	4.E+09	297	3.E+08
B2UMZ5	tr|B2UMZ5|B2UMZ5_AKKM8 Uncharacterized protein OS=Akkermansia muciniphila (strain ATCC BAA-835 / DSM 22959 / JCM 33894 / BCRC 81048 / CCUG 64013 / CIP 107961 / Muc) OX=349741 GN=Amuc_0362 PE=4 SV=1	Amuc_0362	2	2	19.30	15.36	140	49.49	2.E+09	70	2.E+08
B2UMM9	tr|B2UMM9|B2UMM9_AKKM8 Uncharacterized protein OS=Akkermansia muciniphila (strain ATCC BAA-835 / DSM 22959 / JCM 33894 / BCRC 81048 / CCUG 64013 / CIP 107961 / Muc) OX=349741 GN=Amuc_1767 PE=4 SV=1	Amuc_1767	11	11	47.60	32.22	296	317.01	4.E+09	236	2.E+08
B2UP96	tr|B2UP96|B2UP96_AKKM8 Thioredoxin domain-containing protein OS=Akkermansia muciniphila (strain ATCC BAA-835 / DSM 22959 / JCM 33894 / BCRC 81048 / CCUG 64013 / CIP 107961 / Muc) OX=349741 GN=Amuc_2057 PE=4 SV=1	Amuc_2057	4	4	29.30	18.12	167	48.08	2.E+09	144	2.E+08

B2ULL3	tr|B2ULL3|B2ULL3_AKKM8 Nucleoside-diphosphate kinase OS=Akkermansia muciniphila (strain ATCC BAA-835 / DSM 22959 / JCM 33894 / BCRC 81048 / CCUG 64013 / CIP 107961 / Muc) OX=349741 GN=Amuc_1580 PE=3 SV=1	Amuc_1580	6	6	41.70	16.91	151	72.83	3.E+09	130	2.E+08
B2ULU7	tr|B2ULU7|B2ULU7_AKKM8 Thioredoxin domain OS=Akkermansia muciniphila (strain ATCC BAA-835 / DSM 22959 / JCM 33894 / BCRC 81048 / CCUG 64013 / CIP 107961 / Muc) OX=349741 GN=Amuc_0147 PE=4 SV=1	Amuc_0147	9	9	29.60	34.97	321	86.17	4.E+09	274	2.E+08
B2UP90	tr|B2UP90|B2UP90_AKKM8 Glutamate dehydrogenase OS=Akkermansia muciniphila (strain ATCC BAA-835 / DSM 22959 / JCM 33894 / BCRC 81048 / CCUG 64013 / CIP 107961 / Muc) OX=349741 GN=Amuc_2051 PE=3 SV=1	Amuc_2051	14	14	41.70	49.57	451	226.25	7.E+09	417	2.E+08
B2UQH2	tr|B2UQH2|B2UQH2_AKKM8 Elongation factor P OS=Akkermansia muciniphila (strain ATCC BAA-835 / DSM 22959 / JCM 33894 / BCRC 81048 / CCUG 64013 / CIP 107961 / Muc) OX=349741 GN=efp PE=3 SV=1	Amuc_0874	4	4	23.40	20.91	188	93.81	2.E+09	50	2.E+08
B2UPN4	tr|B2UPN4|B2UPN4_AKKM8 Methionine--tRNA ligase OS=Akkermansia muciniphila (strain ATCC BAA-835 / DSM 22959 / JCM 33894 / BCRC 81048 / CCUG 64013 / CIP 107961 / Muc) OX=349741 GN=Amuc_2103 PE=3 SV=1	Amuc_2103	9	9	19.30	62.13	550	235.75	5.E+09	320	2.E+08
B2UPY9	tr|B2UPY9|B2UPY9_AKKM8 Outer membrane autotransporter barrel domain protein OS=Akkermansia muciniphila (strain ATCC BAA-835 / DSM 22959 / JCM 33894 / BCRC 81048 / CCUG 64013 / CIP 107961 / Muc) OX=349741 GN=Amuc_0687 PE=4 SV=1	Amuc_0687	13	13	27.30	107.54	1044	323.31	8.E+09	284	2.E+08
B2UQ32	tr|B2UQ32|B2UQ32_AKKM8 ATP-grasp domain-containing protein OS=Akkermansia muciniphila (strain ATCC BAA-835 / DSM 22959 / JCM 33894 / BCRC 81048 / CCUG 64013 / CIP 107961 / Muc) OX=349741 GN=Amuc_0730 PE=4 SV=1	Amuc_0730	9	9	33.70	38.30	341	127.29	4.E+09	366	2.E+08
B2UQB4	tr|B2UQB4|B2UQB4_AKKM8 Beta sliding clamp OS=Akkermansia muciniphila (strain ATCC BAA-835 / DSM 22959 / JCM 33894 / BCRC 81048 / CCUG 64013 / CIP 107961 / Muc) OX=349741 GN=Amuc_0816 PE=3 SV=1	Amuc_0816	11	11	42.30	40.54	366	168.71	5.E+09	246	2.E+08
B2UQ98	tr|B2UQ98|B2UQ98_AKKM8 Pyruvate flavodoxin/ferredoxin oxidoreductase domain protein OS=Akkermansia muciniphila (strain ATCC BAA-835 / DSM 22959 / JCM 33894 / BCRC 81048 / CCUG 64013 / CIP 107961 / Muc) OX=349741 GN=Amuc_0799 PE=3 SV=1	Amuc_0799	22	22	27.50	129.66	1192	323.31	1.E+10	737	2.E+08
B2ULC0	tr|B2ULC0|B2ULC0_AKKM8 5-oxopent-3-ene-1,2,5-tricarboxylate decarboxylase OS=Akkermansia muciniphila (strain ATCC BAA-835 / DSM 22959 / JCM 33894 / BCRC 81048 / CCUG 64013 / CIP 107961 / Muc) OX=349741 GN=Amuc_0064 PE=4 SV=1	Amuc_0064	7	7	36.00	31.33	292	130.07	3.E+09	226	2.E+08
B2UN16	tr|B2UN16|B2UN16_AKKM8 NAD(P)H dehydrogenase (Quinone) OS=Akkermansia muciniphila (strain ATCC BAA-835 / DSM 22959 / JCM 33894 / BCRC 81048 / CCUG 64013 / CIP 107961 / Muc) OX=349741 GN=Amuc_1809 PE=4 SV=1	Amuc_1809	5	5	26.50	20.95	185	48.54	2.E+09	122	2.E+08
B2UQ84	tr|B2UQ84|B2UQ84_AKKM8 3-isopropylmalate dehydrogenase OS=Akkermansia muciniphila (strain ATCC BAA-835 / DSM 22959 / JCM 33894 / BCRC 81048 / CCUG 64013 / CIP 107961 / Muc) OX=349741 GN=leuB PE=3 SV=1	Amuc_0785	10	10	29.00	39.80	369	148.93	4.E+09	271	2.E+08
B2UNN8	tr|B2UNN8|B2UNN8_AKKM8 Adenylosuccinate lyase OS=Akkermansia muciniphila (strain ATCC BAA-835 / DSM 22959 / JCM 33894 / BCRC 81048 / CCUG 64013 / CIP 107961 / Muc) OX=349741 GN=Amuc_1941 PE=3 SV=1	Amuc_1941	12	12	33.50	52.42	474	272.31	5.E+09	378	2.E+08
B2UNE7	tr|B2UNE7|B2UNE7_AKKM8 Glutamine--tRNA ligase OS=Akkermansia muciniphila (strain ATCC BAA-835 / DSM 22959 / JCM 33894 / BCRC 81048 / CCUG 64013 / CIP 107961 / Muc) OX=349741 GN=glnS PE=3 SV=1	Amuc_0421	13	13	32.20	64.11	559	251.66	6.E+09	305	2.E+08
B2UMG2	tr|B2UMG2|B2UMG2_AKKM8 Histidine triad (HIT) protein OS=Akkermansia muciniphila (strain ATCC BAA-835 / DSM 22959 / JCM 33894 / BCRC 81048 / CCUG 64013 / CIP 107961 / Muc) OX=349741 GN=Amuc_0268 PE=4 SV=1	Amuc_0268	2	2	21.90	12.49	114	22.34	1.E+09	82	2.E+08
B2UM17	sp|B2UM17|FOLD_AKKM8 Bifunctional protein FolD OS=Akkermansia muciniphila (strain ATCC BAA-835 / DSM 22959 / JCM 33894 / BCRC 81048 / CCUG 64013 / CIP 107961 / Muc) OX=349741 GN=folD PE=3 SV=1	Amuc_1641	10	10	51.20	30.85	291	136.95	3.E+09	230	2.E+08
B2UPP6	sp|B2UPP6|SYS_AKKM8 Serine--tRNA ligase OS=Akkermansia muciniphila (strain ATCC BAA-835 / DSM 22959 / JCM 33894 / BCRC 81048 / CCUG 64013 / CIP 107961 / Muc) OX=349741 GN=serS PE=3 SV=1	Amuc_2115	7	7	21.60	47.90	426	128.81	3.E+09	208	2.E+08

B2UPN2	tr|B2UPN2|B2UPN2_AKKM8 D-ribose pyranase OS=Akkermansia muciniphila (strain ATCC BAA-835 / DSM 22959 / JCM 33894 / BCRC 81048 / CCUG 64013 / CIP 107961 / Muc) OX=349741 GN=Amuc_2101 PE=4 SV=1	Amuc_2101	4	4	36.70	15.08	139	39.18	8.E+08	69	2.E+08
B2UQ02	tr|B2UQ02|B2UQ02_AKKM8 Uncharacterized protein OS=Akkermansia muciniphila (strain ATCC BAA-835 / DSM 22959 / JCM 33894 / BCRC 81048 / CCUG 64013 / CIP 107961 / Muc) OX=349741 GN=Amuc_0700 PE=4 SV=1	Amuc_0700	2	2	13.70	15.12	139	51.76	5.E+08	26	2.E+08
B2UNE0	sp|B2UNE0|GMHA_AKKM8 Phosphoheptose isomerase OS=Akkermansia muciniphila (strain ATCC BAA-835 / DSM 22959 / JCM 33894 / BCRC 81048 / CCUG 64013 / CIP 107961 / Muc) OX=349741 GN=gmhA PE=3 SV=1	Amuc_0414	3	3	20.60	20.11	189	73.52	1.E+09	132	2.E+08
B2UQT2	tr|B2UQT2|B2UQT2_AKKM8 TatD-related deoxyribonuclease OS=Akkermansia muciniphila (strain ATCC BAA-835 / DSM 22959 / JCM 33894 / BCRC 81048 / CCUG 64013 / CIP 107961 / Muc) OX=349741 GN=Amuc_0988 PE=4 SV=1	Amuc_0988	6	6	32.50	30.79	271	102.11	3.E+09	192	2.E+08
B2UMM5	tr|B2UMM5|B2UMM5_AKKM8 Pyridoxal phosphate homeostasis protein OS=Akkermansia muciniphila (strain ATCC BAA-835 / DSM 22959 / JCM 33894 / BCRC 81048 / CCUG 64013 / CIP 107961 / Muc) OX=349741 GN=Amuc_1763 PE=3 SV=1	Amuc_1763	5	5	27.70	25.63	231	63.66	2.E+09	246	1.E+08
B2UN34	tr|B2UN34|B2UN34_AKKM8 Pirin domain protein OS=Akkermansia muciniphila (strain ATCC BAA-835 / DSM 22959 / JCM 33894 / BCRC 81048 / CCUG 64013 / CIP 107961 / Muc) OX=349741 GN=Amuc_1827 PE=3 SV=1	Amuc_1827	4	4	12.10	26.34	232	15.68	2.E+09	105	1.E+08
B2UNI4	tr|B2UNI4|B2UNI4_AKKM8 Glutamine amidotransferase class-II OS=Akkermansia muciniphila (strain ATCC BAA-835 / DSM 22959 / JCM 33894 / BCRC 81048 / CCUG 64013 / CIP 107961 / Muc) OX=349741 GN=Amuc_0458 PE=4 SV=1	Amuc_0458	18	18	33.30	72.22	639	236.68	4.E+09	410	1.E+08
B2UM77	tr|B2UM77|B2UM77_AKKM8 Ferritin OS=Akkermansia muciniphila (strain ATCC BAA-835 / DSM 22959 / JCM 33894 / BCRC 81048 / CCUG 64013 / CIP 107961 / Muc) OX=349741 GN=Amuc_1703 PE=3 SV=1	Amuc_1703	3	3	17.10	18.97	164	16.60	1.E+09	84	1.E+08
B2ULR5	tr|B2ULR5|B2ULR5_AKKM8 N5-carboxyaminoimidazole ribonucleotide mutase OS=Akkermansia muciniphila (strain ATCC BAA-835 / DSM 22959 / JCM 33894 / BCRC 81048 / CCUG 64013 / CIP 107961 / Muc) OX=349741 GN=purE PE=3 SV=1	Amuc_0114	4	4	40.40	14.14	136	30.20	8.E+08	69	1.E+08
B2ULP8	tr|B2ULP8|B2ULP8_AKKM8 ROK family protein OS=Akkermansia muciniphila (strain ATCC BAA-835 / DSM 22959 / JCM 33894 / BCRC 81048 / CCUG 64013 / CIP 107961 / Muc) OX=349741 GN=Amuc_0097 PE=4 SV=1	Amuc_0097	5	5	22.60	33.91	319	76.81	2.E+09	164	1.E+08
B2UME9	tr|B2UME9|B2UME9_AKKM8 3-methyl-2-oxobutanoate hydroxymethyltransferase OS=Akkermansia muciniphila (strain ATCC BAA-835 / DSM 22959 / JCM 33894 / BCRC 81048 / CCUG 64013 / CIP 107961 / Muc) OX=349741 GN=panB PE=3 SV=1	Amuc_0255	6	6	30.50	27.64	262	65.83	1.E+09	180	1.E+08
B2UR09	sp|B2UR09|METK_AKKM8 S-adenosylmethionine synthase OS=Akkermansia muciniphila (strain ATCC BAA-835 / DSM 22959 / JCM 33894 / BCRC 81048 / CCUG 64013 / CIP 107961 / Muc) OX=349741 GN=metK PE=3 SV=1	Amuc_1068	9	9	29.20	43.52	394	58.07	3.E+09	145	1.E+08
B2UQ68	tr|B2UQ68|B2UQ68_AKKM8 Ferripyochelin binding protein (Fbp) OS=Akkermansia muciniphila (strain ATCC BAA-835 / DSM 22959 / JCM 33894 / BCRC 81048 / CCUG 64013 / CIP 107961 / Muc) OX=349741 GN=Amuc_0768 PE=4 SV=1	Amuc_0768	2	2	14.00	19.07	178	8.97	5.E+08	108	1.E+08
B2UPW8	sp|B2UPW8|LEUC_AKKM8 3-isopropylmalate dehydratase large subunit OS=Akkermansia muciniphila (strain ATCC BAA-835 / DSM 22959 / JCM 33894 / BCRC 81048 / CCUG 64013 / CIP 107961 / Muc) OX=349741 GN=leuC PE=3 SV=1	Amuc_0666	9	9	27.00	50.53	470	94.34	3.E+09	166	1.E+08
B2UPA8	tr|B2UPA8|B2UPA8_AKKM8 Catalase OS=Akkermansia muciniphila (strain ATCC BAA-835 / DSM 22959 / JCM 33894 / BCRC 81048 / CCUG 64013 / CIP 107961 / Muc) OX=349741 GN=Amuc_2070 PE=3 SV=1	Amuc_2070	17	17	29.60	84.03	751	146.98	4.E+09	296	1.E+08
B2UR10	tr|B2UR10|B2UR10_AKKM8 Adenosylhomocysteinase OS=Akkermansia muciniphila (strain ATCC BAA-835 / DSM 22959 / JCM 33894 / BCRC 81048 / CCUG 64013 / CIP 107961 / Muc) OX=349741 GN=ahcY PE=3 SV=1	Amuc_1069	7	7	23.10	52.07	471	123.87	3.E+09	214	1.E+08
B2UPH1	tr|B2UPH1|B2UPH1_AKKM8 Phosphoribosyl-AMP cyclohydrolase OS=Akkermansia muciniphila (strain ATCC BAA-835 / DSM 22959 / JCM 33894 / BCRC 81048 / CCUG 64013 / CIP 107961 / Muc) OX=349741 GN=hisI PE=3 SV=1	Amuc_0611	3	3	23.30	17.86	163	9.66	8.E+08	52	1.E+08
B2UN13	tr|B2UN13|B2UN13_AKKM8 Carboxymuconolactone decarboxylase OS=Akkermansia muciniphila (strain ATCC BAA-835 / DSM 22959 / JCM 33894 /	Amuc_1806	6	6	29.40	28.21	255	94.40	2.E+09	161	1.E+08

	BCRC 81048 / CCUG 64013 / CIP 107961 / Muc) OX=349741 GN=Amuc_1806 PE=4 SV=1										
B2ULU8	tr|B2ULU8|B2ULU8_AKKM8 Saccharopine dehydrogenase OS=Akkermansia muciniphila (strain ATCC BAA-835 / DSM 22959 / JCM 33894 / BCRC 81048 / CCUG 64013 / CIP 107961 / Muc) OX=349741 GN=Amuc_0148 PE=4 SV=1	Amuc_0148	7	7	28.10	45.82	409	119.97	2.E+09	185	1.E+08
B2UKV6	sp|B2UKV6|DNAK_AKKM8 Chaperone protein DnaK OS=Akkermansia muciniphila (strain ATCC BAA-835 / DSM 22959 / JCM 33894 / BCRC 81048 / CCUG 64013 / CIP 107961 / Muc) OX=349741 GN=dnaK PE=3 SV=1	Amuc_1406	13	13	29.30	68.85	642	196.07	3.E+09	365	1.E+08
B2UR52	sp|B2UR52|ILVD_AKKM8 Dihydroxy-acid dehydratase OS=Akkermansia muciniphila (strain ATCC BAA-835 / DSM 22959 / JCM 33894 / BCRC 81048 / CCUG 64013 / CIP 107961 / Muc) OX=349741 GN=ilvD PE=3 SV=1	Amuc_1111	9	9	24.60	59.48	558	87.53	2.E+09	96	1.E+08
B2UPP1	tr|B2UPP1|B2UPP1_AKKM8 Orotate phosphoribosyltransferase OS=Akkermansia muciniphila (strain ATCC BAA-835 / DSM 22959 / JCM 33894 / BCRC 81048 / CCUG 64013 / CIP 107961 / Muc) OX=349741 GN=pyrE PE=3 SV=1	Amuc_2110	4	4	33.90	20.13	189	46.38	9.E+08	30	1.E+08
B2UQ23	tr|B2UQ23|B2UQ23_AKKM8 Fructose-bisphosphate aldolase OS=Akkermansia muciniphila (strain ATCC BAA-835 / DSM 22959 / JCM 33894 / BCRC 81048 / CCUG 64013 / CIP 107961 / Muc) OX=349741 GN=Amuc_0721 PE=3 SV=1	Amuc_0721	7	7	33.20	36.92	343	133.49	2.E+09	164	1.E+08
B2UPS6	tr|B2UPS6|B2UPS6_AKKM8 6-carboxy-5,6,7,8-tetrahydropterin synthase OS=Akkermansia muciniphila (strain ATCC BAA-835 / DSM 22959 / JCM 33894 / BCRC 81048 / CCUG 64013 / CIP 107961 / Muc) OX=349741 GN=Amuc_2146 PE=3 SV=1	Amuc_2146	4	4	32.70	17.24	150	29.24	9.E+08	74	1.E+08
B2UNH4	sp|B2UNH4|GCSP_AKKM8 Glycine dehydrogenase (decarboxylating) OS=Akkermansia muciniphila (strain ATCC BAA-835 / DSM 22959 / JCM 33894 / BCRC 81048 / CCUG 64013 / CIP 107961 / Muc) OX=349741 GN=gcvP PE=3 SV=1	Amuc_0448	12	12	15.80	102.65	948	167.26	4.E+09	140	1.E+08
B2UM95	tr|B2UM95|B2UM95_AKKM8 Methylmalonyl-CoA epimerase OS=Akkermansia muciniphila (strain ATCC BAA-835 / DSM 22959 / JCM 33894 / BCRC 81048 / CCUG 64013 / CIP 107961 / Muc) OX=349741 GN=Amuc_0200 PE=3 SV=1	Amuc_0200	2	2	19.30	15.05	140	16.76	8.E+08	49	1.E+08
B2UL88	tr|B2UL88|B2UL88_AKKM8 APH domain-containing protein OS=Akkermansia muciniphila (strain ATCC BAA-835 / DSM 22959 / JCM 33894 / BCRC 81048 / CCUG 64013 / CIP 107961 / Muc) OX=349741 GN=Amuc_0030 PE=4 SV=1	Amuc_0030	10	10	31.60	41.66	364	98.24	3.E+09	286	1.E+08
B2UQP6	tr|B2UQP6|B2UQP6_AKKM8 Peptidyl-prolyl cis-trans isomerase OS=Akkermansia muciniphila (strain ATCC BAA-835 / DSM 22959 / JCM 33894 / BCRC 81048 / CCUG 64013 / CIP 107961 / Muc) OX=349741 GN=Amuc_0949 PE=3 SV=1	Amuc_0949	2	2	12.70	20.97	197	47.03	6.E+08	127	1.E+08
B2UM88	tr|B2UM88|B2UM88_AKKM8 Cysteine desulfurase OS=Akkermansia muciniphila (strain ATCC BAA-835 / DSM 22959 / JCM 33894 / BCRC 81048 / CCUG 64013 / CIP 107961 / Muc) OX=349741 GN=Amuc_0193 PE=3 SV=1	Amuc_0193	10	10	37.30	44.14	408	88.82	2.E+09	151	1.E+08
B2UL59	tr|B2UL59|B2UL59_AKKM8 NUDIX hydrolase OS=Akkermansia muciniphila (strain ATCC BAA-835 / DSM 22959 / JCM 33894 / BCRC 81048 / CCUG 64013 / CIP 107961 / Muc) OX=349741 GN=Amuc_0001 PE=4 SV=1	Amuc_0001	3	3	27.40	20.60	186	60.00	7.E+08	60	9.E+07
B2UNG5	sp|B2UNG5|RS13_AKKM8 30S ribosomal protein S13 OS=Akkermansia muciniphila (strain ATCC BAA-835 / DSM 22959 / JCM 33894 / BCRC 81048 / CCUG 64013 / CIP 107961 / Muc) OX=349741 GN=rpsM PE=3 SV=1	Amuc_0439	3	3	31.20	14.21	125	31.36	6.E+08	29	9.E+07
B2UMU1	sp|B2UMU1|RS10_AKKM8 30S ribosomal protein S10 OS=Akkermansia muciniphila (strain ATCC BAA-835 / DSM 22959 / JCM 33894 / BCRC 81048 / CCUG 64013 / CIP 107961 / Muc) OX=349741 GN=rpsJ PE=3 SV=1	Amuc_0305	2	2	21.60	11.71	102	13.75	4.E+08	35	9.E+07
B2UN72	tr|B2UN72|B2UN72_AKKM8 Glycosyl transferase group 1 OS=Akkermansia muciniphila (strain ATCC BAA-835 / DSM 22959 / JCM 33894 / BCRC 81048 / CCUG 64013 / CIP 107961 / Muc) OX=349741 GN=Amuc_1869 PE=4 SV=1	Amuc_1869	12	12	30.10	47.87	432	120.81	2.E+09	139	9.E+07
B2UM63	tr|B2UM63|B2UM63_AKKM8 Dihydrolipoyl dehydrogenase OS=Akkermansia muciniphila (strain ATCC BAA-835 / DSM 22959 / JCM 33894 / BCRC 81048 / CCUG 64013 / CIP 107961 / Muc) OX=349741 GN=Amuc_1689 PE=3 SV=1	Amuc_1689	9	9	25.30	48.95	462	92.78	2.E+09	159	9.E+07

B2ULD3	tr|B2ULD3|B2ULD3_AKKM8 2-amino-3-ketobutyrate coenzyme A ligase OS=Akkermansia muciniphila (strain ATCC BAA-835 / DSM 22959 / JCM 33894 / BCRC 81048 / CCUG 64013 / CIP 107961 / Muc) OX=349741 GN=kbl PE=3 SV=1	Amuc_0077	8	8	27.60	43.36	399	102.03	2.E+09	156	9.E+07
B2UQY3	sp|B2UQY3|RL7_AKKM8 50S ribosomal protein L7/L12 OS=Akkermansia muciniphila (strain ATCC BAA-835 / DSM 22959 / JCM 33894 / BCRC 81048 / CCUG 64013 / CIP 107961 / Muc) OX=349741 GN=rplL PE=3 SV=1	Amuc_1042	3	3	27.40	12.64	124	57.71	6.E+08	60	9.E+07
B2UP74	tr|B2UP74|B2UP74_AKKM8 Uncharacterized protein OS=Akkermansia muciniphila (strain ATCC BAA-835 / DSM 22959 / JCM 33894 / BCRC 81048 / CCUG 64013 / CIP 107961 / Muc) OX=349741 GN=Amuc_2035 PE=4 SV=1	Amuc_2035	4	4	22.30	27.43	251	116.43	1.E+09	59	9.E+07
B2UNZ8	tr|B2UNZ8|B2UNZ8_AKKM8 Transketolase OS=Akkermansia muciniphila (strain ATCC BAA-835 / DSM 22959 / JCM 33894 / BCRC 81048 / CCUG 64013 / CIP 107961 / Muc) OX=349741 GN=Amuc_0530 PE=3 SV=1	Amuc_0530	17	17	33.50	72.86	677	157.84	3.E+09	296	8.E+07
B2UP35	tr|B2UP35|B2UP35_AKKM8 Tryptophanase OS=Akkermansia muciniphila (strain ATCC BAA-835 / DSM 22959 / JCM 33894 / BCRC 81048 / CCUG 64013 / CIP 107961 / Muc) OX=349741 GN=Amuc_1995 PE=3 SV=1	Amuc_1995	7	7	17.90	54.00	487	107.46	2.E+09	195	8.E+07
B2UP77	tr|B2UP77|B2UP77_AKKM8 PUR-alpha/beta/gamma DNA/RNA-binding OS=Akkermansia muciniphila (strain ATCC BAA-835 / DSM 22959 / JCM 33894 / BCRC 81048 / CCUG 64013 / CIP 107961 / Muc) OX=349741 GN=Amuc_2038 PE=4 SV=1	Amuc_2038	4	4	52.90	8.11	70	11.84	2.E+08	51	8.E+07
B2URK3	tr|B2URK3|B2URK3_AKKM8 Quinolinate phosphoribosyltransferase [decarboxylating] OS=Akkermansia muciniphila (strain ATCC BAA-835 / DSM 22959 / JCM 33894 / BCRC 81048 / CCUG 64013 / CIP 107961 / Muc) OX=349741 GN=Amuc_1263 PE=3 SV=1	Amuc_1263	3	3	13.90	30.91	287	48.31	8.E+08	108	8.E+07
B2UPP7	tr|B2UPP7|B2UPP7_AKKM8 Alcohol dehydrogenase zinc-binding domain protein OS=Akkermansia muciniphila (strain ATCC BAA-835 / DSM 22959 / JCM 33894 / BCRC 81048 / CCUG 64013 / CIP 107961 / Muc) OX=349741 GN=Amuc_2116 PE=3 SV=1	Amuc_2116	7	7	26.80	40.38	373	64.36	1.E+09	184	8.E+07
B2UKV8	sp|B2UKV8|CH60_AKKM8 60 kDa chaperonin OS=Akkermansia muciniphila (strain ATCC BAA-835 / DSM 22959 / JCM 33894 / BCRC 81048 / CCUG 64013 / CIP 107961 / Muc) OX=349741 GN=groL PE=3 SV=1	Amuc_1408	11	11	29.10	58.44	550	157.91	3.E+09	247	7.E+07
B2UM62	tr|B2UM62|B2UM62_AKKM8 Amidohydrolase OS=Akkermansia muciniphila (strain ATCC BAA-835 / DSM 22959 / JCM 33894 / BCRC 81048 / CCUG 64013 / CIP 107961 / Muc) OX=349741 GN=Amuc_1688 PE=4 SV=1	Amuc_1688	4	4	15.20	47.60	440	55.44	1.E+09	55	7.E+07
B2UPW3	tr|B2UPW3|B2UPW3_AKKM8 D-alanine--D-alanine ligase OS=Akkermansia muciniphila (strain ATCC BAA-835 / DSM 22959 / JCM 33894 / BCRC 81048 / CCUG 64013 / CIP 107961 / Muc) OX=349741 GN=ddl PE=3 SV=1	Amuc_0661	3	3	10.30	33.30	311	23.44	1.E+09	89	7.E+07
B2UNP4	tr|B2UNP4|B2UNP4_AKKM8 N-acylglucosamine 2-epimerase OS=Akkermansia muciniphila (strain ATCC BAA-835 / DSM 22959 / JCM 33894 / BCRC 81048 / CCUG 64013 / CIP 107961 / Muc) OX=349741 GN=Amuc_1947 PE=4 SV=1	Amuc_1947	6	6	20.60	45.60	393	31.79	1.E+09	124	6.E+07
B2URQ1	tr|B2URQ1|B2URQ1_AKKM8 Peptidase M16 domain protein OS=Akkermansia muciniphila (strain ATCC BAA-835 / DSM 22959 / JCM 33894 / BCRC 81048 / CCUG 64013 / CIP 107961 / Muc) OX=349741 GN=Amuc_1312 PE=4 SV=1	Amuc_1312	9	9	14.90	93.01	840	150.12	2.E+09	211	6.E+07
B2UQL1	tr|B2UQL1|B2UQL1_AKKM8 Cupin 2 conserved barrel domain protein OS=Akkermansia muciniphila (strain ATCC BAA-835 / DSM 22959 / JCM 33894 / BCRC 81048 / CCUG 64013 / CIP 107961 / Muc) OX=349741 GN=Amuc_0914 PE=4 SV=1	Amuc_0914	2	2	18.80	12.31	112	11.16	3.E+08	34	6.E+07
B2UNJ7	tr|B2UNJ7|B2UNJ7_AKKM8 Flavodoxin/nitric oxide synthase OS=Akkermansia muciniphila (strain ATCC BAA-835 / DSM 22959 / JCM 33894 / BCRC 81048 / CCUG 64013 / CIP 107961 / Muc) OX=349741 GN=Amuc_1899 PE=4 SV=1	Amuc_1899	2	2	14.60	15.84	144	16.15	3.E+08	31	6.E+07
B2UN86	tr|B2UN86|B2UN86_AKKM8 Beta-lactamase domain protein OS=Akkermansia muciniphila (strain ATCC BAA-835 / DSM 22959 / JCM 33894 / BCRC 81048 / CCUG 64013 / CIP 107961 / Muc) OX=349741 GN=Amuc_1883 PE=4 SV=1	Amuc_1883	7	7	20.20	50.21	456	38.43	1.E+09	90	6.E+07
B2UPT0	tr|B2UPT0|B2UPT0_AKKM8 Quinolinate synthase OS=Akkermansia muciniphila (strain ATCC BAA-835 / DSM 22959 / JCM 33894 / BCRC 81048 / CCUG 64013 / CIP 107961 / Muc) OX=349741 GN=Amuc_2150 PE=4 SV=1	Amuc_2150	3	3	15.80	34.45	311	47.91	1.E+09	59	6.E+07

B2UKP4	tr|B2UKP4|B2UKP4_AKKM8 Uncharacterized protein OS=Akkermansia muciniphila (strain ATCC BAA-835 / DSM 22959 / JCM 33894 / BCRC 81048 / CCUG 64013 / CIP 107961 / Muc) OX=349741 GN=Amuc_1343 PE=4 SV=1	Amuc_1343	2	2	13.30	20.32	188	12.68	4.E+08	41	5.E+07
B2UNX2	tr|B2UNX2|B2UNX2_AKKM8 DUF5069 domain-containing protein OS=Akkermansia muciniphila (strain ATCC BAA-835 / DSM 22959 / JCM 33894 / BCRC 81048 / CCUG 64013 / CIP 107961 / Muc) OX=349741 GN=Amuc_0504 PE=4 SV=1	Amuc_0504	2	2	14.40	21.45	187	13.69	5.E+08	100	5.E+07
B2UMI8	tr|B2UMI8|B2UMI8_AKKM8 UDP-glucuronate decarboxylase OS=Akkermansia muciniphila (strain ATCC BAA-835 / DSM 22959 / JCM 33894 / BCRC 81048 / CCUG 64013 / CIP 107961 / Muc) OX=349741 GN=Amuc_1726 PE=3 SV=1	Amuc_1726	6	6	20.60	35.03	310	47.48	8.E+08	163	5.E+07
B2UPX3	tr|B2UPX3|B2UPX3_AKKM8 3-dehydroquinate synthase OS=Akkermansia muciniphila (strain ATCC BAA-835 / DSM 22959 / JCM 33894 / BCRC 81048 / CCUG 64013 / CIP 107961 / Muc) OX=349741 GN=Amuc_0671 PE=4 SV=1	Amuc_0671	4	4	13.20	42.15	387	47.64	1.E+09	121	5.E+07
B2URI5	tr|B2URI5|B2URI5_AKKM8 Mannose-6-phosphate isomerase OS=Akkermansia muciniphila (strain ATCC BAA-835 / DSM 22959 / JCM 33894 / BCRC 81048 / CCUG 64013 / CIP 107961 / Muc) OX=349741 GN=Amuc_1245 PE=3 SV=1	Amuc_1245	8	8	32.70	34.83	306	39.99	7.E+08	60	5.E+07
B2UNZ3	tr|B2UNZ3|B2UNZ3_AKKM8 Cycloartenol synthase-like protein OS=Akkermansia muciniphila (strain ATCC BAA-835 / DSM 22959 / JCM 33894 / BCRC 81048 / CCUG 64013 / CIP 107961 / Muc) OX=349741 GN=Amuc_0525 PE=4 SV=1	Amuc_0525	8	8	30.40	43.36	392	42.09	1.E+09	69	5.E+07
B2UM18	tr|B2UM18|B2UM18_AKKM8 Enoyl-[acyl-carrier-protein] reductase [NADH] OS=Akkermansia muciniphila (strain ATCC BAA-835 / DSM 22959 / JCM 33894 / BCRC 81048 / CCUG 64013 / CIP 107961 / Muc) OX=349741 GN=Amuc_1642 PE=3 SV=1	Amuc_1642	5	5	19.60	27.88	260	26.48	8.E+08	75	5.E+07
B2UKX4	tr|B2UKX4|B2UKX4_AKKM8 Kelch repeat-containing protein OS=Akkermansia muciniphila (strain ATCC BAA-835 / DSM 22959 / JCM 33894 / BCRC 81048 / CCUG 64013 / CIP 107961 / Muc) OX=349741 GN=Amuc_1425 PE=4 SV=1	Amuc_1425	2	2	9.20	35.13	327	29.39	7.E+08	56	5.E+07
B2UQX4	tr|B2UQX4|B2UQX4_AKKM8 Sulfatase OS=Akkermansia muciniphila (strain ATCC BAA-835 / DSM 22959 / JCM 33894 / BCRC 81048 / CCUG 64013 / CIP 107961 / Muc) OX=349741 GN=Amuc_1033 PE=3 SV=1	Amuc_1033	9	9	18.20	67.00	581	42.47	2.E+09	103	5.E+07
B2UM28	tr|B2UM28|B2UM28_AKKM8 Ribulose-phosphate 3-epimerase OS=Akkermansia muciniphila (strain ATCC BAA-835 / DSM 22959 / JCM 33894 / BCRC 81048 / CCUG 64013 / CIP 107961 / Muc) OX=349741 GN=rpe PE=3 SV=1	Amuc_1653	3	3	12.40	23.63	217	15.85	2.E+08	12	5.E+07
B2UPD6	tr|B2UPD6|B2UPD6_AKKM8 Peptidase M16 domain protein OS=Akkermansia muciniphila (strain ATCC BAA-835 / DSM 22959 / JCM 33894 / BCRC 81048 / CCUG 64013 / CIP 107961 / Muc) OX=349741 GN=Amuc_0576 PE=3 SV=1	Amuc_0576	22	22	16.90	160.48	1442	199.61	4.E+09	264	5.E+07
B2UQ36	tr|B2UQ36|B2UQ36_AKKM8 YD repeat protein OS=Akkermansia muciniphila (strain ATCC BAA-835 / DSM 22959 / JCM 33894 / BCRC 81048 / CCUG 64013 / CIP 107961 / Muc) OX=349741 GN=Amuc_0735 PE=4 SV=1	Amuc_0735	23	23	19.20	212.82	1938	161.17	4.E+09	172	4.E+07
B2UQY5	tr|B2UQY5|B2UQY5_AKKM8 50S ribosomal protein L1 OS=Akkermansia muciniphila (strain ATCC BAA-835 / DSM 22959 / JCM 33894 / BCRC 81048 / CCUG 64013 / CIP 107961 / Muc) OX=349741 GN=rplA PE=3 SV=1	Amuc_1044	4	4	26.60	24.67	233	41.16	5.E+08	34	4.E+07
B2UQJ3	tr|B2UQJ3|B2UQJ3_AKKM8 PepSY_like domain-containing protein OS=Akkermansia muciniphila (strain ATCC BAA-835 / DSM 22959 / JCM 33894 / BCRC 81048 / CCUG 64013 / CIP 107961 / Muc) OX=349741 GN=Amuc_0895 PE=4 SV=1	Amuc_0895	3	3	24.30	16.87	152	8.63	3.E+08	24	4.E+07
B2UMU3	sp|B2UMU3|RS7_AKKM8 30S ribosomal protein S7 OS=Akkermansia muciniphila (strain ATCC BAA-835 / DSM 22959 / JCM 33894 / BCRC 81048 / CCUG 64013 / CIP 107961 / Muc) OX=349741 GN=rpsG PE=3 SV=1	Amuc_0307	2	2	25.50	17.94	157	28.58	3.E+08	42	4.E+07
B2UL08	tr|B2UL08|B2UL08_AKKM8 Protein GrpE OS=Akkermansia muciniphila (strain ATCC BAA-835 / DSM 22959 / JCM 33894 / BCRC 81048 / CCUG 64013 / CIP 107961 / Muc) OX=349741 GN=grpE PE=3 SV=1	Amuc_1459	2	2	17.90	20.67	184	32.19	2.E+08	16	4.E+07
B2UP30	tr|B2UP30|B2UP30_AKKM8 Pyrroline-5-carboxylate reductase OS=Akkermansia muciniphila (strain ATCC BAA-835 / DSM 22959 / JCM 33894 / BCRC 81048 / CCUG 64013 / CIP 107961 / Muc) OX=349741 GN=proC PE=3 SV=1	Amuc_1990	3	3	15.70	27.92	268	30.52	5.E+08	43	4.E+07
B2URK5	tr|B2URK5|B2URK5_AKKM8 Aspartate-semialdehyde dehydrogenase OS=Akkermansia muciniphila (strain ATCC BAA-835 / DSM 22959 / JCM 33894 / BCRC 81048 / CCUG 64013 / CIP 107961 / Muc) OX=349741 GN=asd PE=3 SV=1	Amuc_1265	4	4	16.70	36.06	336	98.04	7.E+08	38	4.E+07

B2UKZ7	tr|B2UKZ7|B2UKZ7_AKKM8 Arginine biosynthesis bifunctional protein ArgJ OS=Akkermansia muciniphila (strain ATCC BAA-835 / DSM 22959 / JCM 33894 / BCRC 81048 / CCUG 64013 / CIP 107961 / Muc) OX=349741 GN=argJ PE=3 SV=1	Amuc_1448	6	6	19.70	44.44	417	35.58	9.E+08	51	4.E+07
B2UQI1	tr|B2UQI1|B2UQI1_AKKM8 Uncharacterized protein OS=Akkermansia muciniphila (strain ATCC BAA-835 / DSM 22959 / JCM 33894 / BCRC 81048 / CCUG 64013 / CIP 107961 / Muc) OX=349741 GN=Amuc_0883 PE=4 SV=1	Amuc_0883	2	2	21.00	6.74	62	29.80	8.E+07	4	4.E+07
B2UN37	tr|B2UN37|B2UN37_AKKM8 L-fuculokinase OS=Akkermansia muciniphila (strain ATCC BAA-835 / DSM 22959 / JCM 33894 / BCRC 81048 / CCUG 64013 / CIP 107961 / Muc) OX=349741 GN=fucK PE=3 SV=1	Amuc_1830	6	6	20.50	51.25	474	46.09	7.E+08	85	4.E+07
B2UP85	tr|B2UP85|B2UP85_AKKM8 Uncharacterized protein OS=Akkermansia muciniphila (strain ATCC BAA-835 / DSM 22959 / JCM 33894 / BCRC 81048 / CCUG 64013 / CIP 107961 / Muc) OX=349741 GN=Amuc_2046 PE=4 SV=1	Amuc_2046	4	4	27.70	22.81	206	27.75	5.E+08	93	4.E+07
B2UNN9	tr|B2UNN9|B2UNN9_AKKM8 Peptidase T OS=Akkermansia muciniphila (strain ATCC BAA-835 / DSM 22959 / JCM 33894 / BCRC 81048 / CCUG 64013 / CIP 107961 / Muc) OX=349741 GN=pepT PE=3 SV=1	Amuc_1942	5	5	17.70	45.12	417	37.50	6.E+08	48	4.E+07
B2URI3	sp|B2URI3|ISPF_AKKM8 2-C-methyl-D-erythritol 2,4-cyclodiphosphate synthase OS=Akkermansia muciniphila (strain ATCC BAA-835 / DSM 22959 / JCM 33894 / BCRC 81048 / CCUG 64013 / CIP 107961 / Muc) OX=349741 GN=ispF PE=3 SV=1	Amuc_1243	3	3	21.30	17.69	169	15.18	3.E+08	15	4.E+07
B2UL30	tr|B2UL30|B2UL30_AKKM8 ATP-dependent 6-phosphofructokinase OS=Akkermansia muciniphila (strain ATCC BAA-835 / DSM 22959 / JCM 33894 / BCRC 81048 / CCUG 64013 / CIP 107961 / Muc) OX=349741 GN=pfkA PE=3 SV=1	Amuc_1481	5	5	15.90	37.95	359	14.12	7.E+08	53	4.E+07
B2UMH5	sp|B2UMH5|SUCC_AKKM8 Succinate--CoA ligase [ADP-forming] subunit beta OS=Akkermansia muciniphila (strain ATCC BAA-835 / DSM 22959 / JCM 33894 / BCRC 81048 / CCUG 64013 / CIP 107961 / Muc) OX=349741 GN=sucC PE=3 SV=1	Amuc_1713	6	6	20.40	42.34	393	102.02	1.E+09	77	4.E+07
B2UMH4	tr|B2UMH4|B2UMH4_AKKM8 Succinate--CoA ligase [ADP-forming] subunit alpha OS=Akkermansia muciniphila (strain ATCC BAA-835 / DSM 22959 / JCM 33894 / BCRC 81048 / CCUG 64013 / CIP 107961 / Muc) OX=349741 GN=sucD PE=3 SV=1	Amuc_1712	4	4	18.60	30.57	295	20.75	5.E+08	39	4.E+07
B2UP52	tr|B2UP52|B2UP52_AKKM8 O-acetylhomoserine/O-acetylserine sulfhydrylase OS=Akkermansia muciniphila (strain ATCC BAA-835 / DSM 22959 / JCM 33894 / BCRC 81048 / CCUG 64013 / CIP 107961 / Muc) OX=349741 GN=Amuc_2013 PE=3 SV=1	Amuc_2013	3	3	8.40	46.41	428	32.68	7.E+08	51	4.E+07
B2UQI4	tr|B2UQI4|B2UQI4_AKKM8 Transketolase central region OS=Akkermansia muciniphila (strain ATCC BAA-835 / DSM 22959 / JCM 33894 / BCRC 81048 / CCUG 64013 / CIP 107961 / Muc) OX=349741 GN=Amuc_0886 PE=4 SV=1	Amuc_0886	7	7	30.90	35.59	324	53.97	6.E+08	81	4.E+07
B2UQG5	tr|B2UQG5|B2UQG5_AKKM8 Peptidase M42 family protein OS=Akkermansia muciniphila (strain ATCC BAA-835 / DSM 22959 / JCM 33894 / BCRC 81048 / CCUG 64013 / CIP 107961 / Muc) OX=349741 GN=Amuc_0867 PE=3 SV=1	Amuc_0867	5	5	19.10	41.20	382	37.51	7.E+08	38	3.E+07
B2UNI3	tr|B2UNI3|B2UNI3_AKKM8 AIR synthase OS=Akkermansia muciniphila (strain ATCC BAA-835 / DSM 22959 / JCM 33894 / BCRC 81048 / CCUG 64013 / CIP 107961 / Muc) OX=349741 GN=Amuc_0457 PE=3 SV=1	Amuc_0457	5	5	20.10	36.49	333	66.15	6.E+08	55	3.E+07
B2UN30;B2UQW1	tr|B2UN30|B2UN30_AKKM8 Uncharacterized protein OS=Akkermansia muciniphila (strain ATCC BAA-835 / DSM 22959 / JCM 33894 / BCRC 81048 / CCUG 64013 / CIP 107961 / Muc) OX=349741 GN=Amuc_1823 PE=4 SV=1;tr|B2UQW1|B2UQW1_AKKM8 Uncharacterized protein OS=Akkerman	Amuc_1823	6	6	15.40	61.69	545;546	132.90	9.E+08	75	3.E+07
B2UQC2	tr|B2UQC2|B2UQC2_AKKM8 Lactase OS=Akkermansia muciniphila (strain ATCC BAA-835 / DSM 22959 / JCM 33894 / BCRC 81048 / CCUG 64013 / CIP 107961 / Muc) OX=349741 GN=Amuc_0824 PE=3 SV=1	Amuc_0824	20	20	18.00	142.05	1264	124.73	2.E+09	273	3.E+07
B2UNT6	tr|B2UNT6|B2UNT6_AKKM8 Transcriptional regulator, DeoR family OS=Akkermansia muciniphila (strain ATCC BAA-835 / DSM 22959 / JCM 33894 / BCRC 81048 / CCUG 64013 / CIP 107961 / Muc) OX=349741 GN=Amuc_0468 PE=4 SV=1	Amuc_0468	3	3	14.10	27.12	249	15.20	4.E+08	30	3.E+07
B2UQG3	tr|B2UQG3|B2UQG3_AKKM8 Rubrerythrin OS=Akkermansia muciniphila (strain ATCC BAA-835 / DSM 22959 / JCM 33894 / BCRC 81048 / CCUG 64013 / CIP 107961 / Muc) OX=349741 GN=Amuc_0865 PE=4 SV=1	Amuc_0865	4	4	29.70	19.53	182	35.11	3.E+08	31	3.E+07
B2URJ1	tr|B2URJ1|B2URJ1_AKKM8 Carbamoyl-phosphate synthase large chain OS=Akkermansia muciniphila (strain ATCC BAA-835 / DSM 22959 / JCM 33894 / BCRC 81048 / CCUG 64013 / CIP 107961 / Muc) OX=349741 GN=carB PE=3 SV=1	Amuc_1251	16	16	21.00	117.47	1067	120.87	2.E+09	230	3.E+07

B2UQE2	tr|B2UQE2|B2UQE2_AKKM8 Enolase OS=Akkermansia muciniphila (strain ATCC BAA-835 / DSM 22959 / JCM 33894 / BCRC 81048 / CCUG 64013 / CIP 107961 / Muc) OX=349741 GN=eno PE=3 SV=1	Amuc_0844	6	4	19.30	45.97	429	25.17	5.E+08	26	3.E+07
B2UP25	sp|B2UP25|RS14_AKKM8 30S ribosomal protein S14 OS=Akkermansia muciniphila (strain ATCC BAA-835 / DSM 22959 / JCM 33894 / BCRC 81048 / CCUG 64013 / CIP 107961 / Muc) OX=349741 GN=rpsN PE=3 SV=1	Amuc_0557	2	2	25.80	10.28	89	7.50	6.E+07	15	3.E+07
B2UL21	sp|B2UL21|RL25_AKKM8 50S ribosomal protein L25 OS=Akkermansia muciniphila (strain ATCC BAA-835 / DSM 22959 / JCM 33894 / BCRC 81048 / CCUG 64013 / CIP 107961 / Muc) OX=349741 GN=rplY PE=3 SV=1	Amuc_1472	2	2	15.40	21.01	201	14.15	2.E+08	31	3.E+07
B2UN14	tr|B2UN14|B2UN14_AKKM8 Aldo/keto reductase OS=Akkermansia muciniphila (strain ATCC BAA-835 / DSM 22959 / JCM 33894 / BCRC 81048 / CCUG 64013 / CIP 107961 / Muc) OX=349741 GN=Amuc_1807 PE=4 SV=1	Amuc_1807	7	7	33.40	34.57	314	25.97	5.E+08	86	3.E+07
B2UQC0	tr|B2UQC0|B2UQC0_AKKM8 TatD-related deoxyribonuclease OS=Akkermansia muciniphila (strain ATCC BAA-835 / DSM 22959 / JCM 33894 / BCRC 81048 / CCUG 64013 / CIP 107961 / Muc) OX=349741 GN=Amuc_0822 PE=4 SV=1	Amuc_0822	4	4	19.20	30.49	271	25.97	5.E+08	44	3.E+07
B2UQQ6	tr|B2UQQ6|B2UQQ6_AKKM8 dITP/XTP pyrophosphatase OS=Akkermansia muciniphila (strain ATCC BAA-835 / DSM 22959 / JCM 33894 / BCRC 81048 / CCUG 64013 / CIP 107961 / Muc) OX=349741 GN=Amuc_0959 PE=3 SV=1	Amuc_0959	3	3	21.00	23.54	214	12.43	2.E+08	34	3.E+07
B2UNA9	sp|B2UNA9|PNP_AKKM8 Polyribonucleotide nucleotidyltransferase OS=Akkermansia muciniphila (strain ATCC BAA-835 / DSM 22959 / JCM 33894 / BCRC 81048 / CCUG 64013 / CIP 107961 / Muc) OX=349741 GN=pnp PE=3 SV=1	Amuc_0382	8	8	15.80	77.48	714	35.83	9.E+08	84	3.E+07
B2URG3	tr|B2URG3|B2URG3_AKKM8 Orotidine-5-phosphate decarboxylase OS=Akkermansia muciniphila (strain ATCC BAA-835 / DSM 22959 / JCM 33894 / BCRC 81048 / CCUG 64013 / CIP 107961 / Muc) OX=349741 GN=Amuc_1223 PE=4 SV=1	Amuc_1223	4	4	22.70	28.31	264	47.74	4.E+08	49	3.E+07
B2UN11	tr|B2UN11|B2UN11_AKKM8 Flavodoxin-like domain-containing protein OS=Akkermansia muciniphila (strain ATCC BAA-835 / DSM 22959 / JCM 33894 / BCRC 81048 / CCUG 64013 / CIP 107961 / Muc) OX=349741 GN=Amuc_1804 PE=4 SV=1	Amuc_1804	2	2	14.00	19.22	178	8.61	3.E+08	16	3.E+07
B2URD0	tr|B2URD0|B2URD0_AKKM8 Alcohol dehydrogenase zinc-binding domain protein OS=Akkermansia muciniphila (strain ATCC BAA-835 / DSM 22959 / JCM 33894 / BCRC 81048 / CCUG 64013 / CIP 107961 / Muc) OX=349741 GN=Amuc_1190 PE=4 SV=1	Amuc_1190	6	6	25.50	36.08	333	43.43	5.E+08	62	3.E+07
B2ULH7	tr|B2ULH7|B2ULH7_AKKM8 Formate C-acetyltransferase OS=Akkermansia muciniphila (strain ATCC BAA-835 / DSM 22959 / JCM 33894 / BCRC 81048 / CCUG 64013 / CIP 107961 / Muc) OX=349741 GN=Amuc_1543 PE=3 SV=1	Amuc_1543	11	11	20.70	85.23	755	80.99	1.E+09	158	2.E+07
B2ULM1	tr|B2ULM1|B2ULM1_AKKM8 Uncharacterized protein OS=Akkermansia muciniphila (strain ATCC BAA-835 / DSM 22959 / JCM 33894 / BCRC 81048 / CCUG 64013 / CIP 107961 / Muc) OX=349741 GN=Amuc_1588 PE=4 SV=1	Amuc_1588	4	4	27.80	18.54	169	21.44	2.E+08	29	2.E+07
B2UQI5	tr|B2UQI5|B2UQI5_AKKM8 Dehydrogenase E1 component OS=Akkermansia muciniphila (strain ATCC BAA-835 / DSM 22959 / JCM 33894 / BCRC 81048 / CCUG 64013 / CIP 107961 / Muc) OX=349741 GN=Amuc_0887 PE=4 SV=1	Amuc_0887	7	7	23.70	33.92	312	28.90	4.E+08	50	2.E+07
B2ULE0	tr|B2ULE0|B2ULE0_AKKM8 PA14 domain-containing protein OS=Akkermansia muciniphila (strain ATCC BAA-835 / DSM 22959 / JCM 33894 / BCRC 81048 / CCUG 64013 / CIP 107961 / Muc) OX=349741 GN=Amuc_0084 PE=4 SV=1	Amuc_0084	3	3	9.90	61.62	547	47.40	7.E+08	49	2.E+07
B2URI2	tr|B2URI2|B2URI2_AKKM8 Inositol-1-monophosphatase OS=Akkermansia muciniphila (strain ATCC BAA-835 / DSM 22959 / JCM 33894 / BCRC 81048 / CCUG 64013 / CIP 107961 / Muc) OX=349741 GN=Amuc_1242 PE=3 SV=1	Amuc_1242	3	3	14.20	28.52	254	11.42	2.E+08	27	2.E+07
B2UKU8	tr|B2UKU8|B2UKU8_AKKM8 Histidine--tRNA ligase OS=Akkermansia muciniphila (strain ATCC BAA-835 / DSM 22959 / JCM 33894 / BCRC 81048 / CCUG 64013 / CIP 107961 / Muc) OX=349741 GN=Amuc_1397 PE=3 SV=1	Amuc_1397	7	7	20.50	47.05	424	40.52	5.E+08	66	2.E+07
B2UN97	sp|B2UN97|AROA_AKKM8 3-phosphoshikimate 1-carboxyvinyltransferase OS=Akkermansia muciniphila (strain ATCC BAA-835 / DSM 22959 / JCM 33894 / BCRC 81048 / CCUG 64013 / CIP 107961 / Muc) OX=349741 GN=aroA PE=3 SV=1	Amuc_1894	3	3	11.50	46.11	435	19.31	3.E+08	17	2.E+07

B2UL94	tr|B2UL94|B2UL94_AKKM8 YD repeat protein OS=Akkermansia muciniphila (strain ATCC BAA-835 / DSM 22959 / JCM 33894 / BCRC 81048 / CCUG 64013 / CIP 107961 / Muc) OX=349741 GN=Amuc_0036 PE=4 SV=1	Amuc_0036	26	26	19.70	214.69	1957	147.05	2.E+09	211	2.E+07
B2UR15	tr|B2UR15|B2UR15_AKKM8 Sulfatase OS=Akkermansia muciniphila (strain ATCC BAA-835 / DSM 22959 / JCM 33894 / BCRC 81048 / CCUG 64013 / CIP 107961 / Muc) OX=349741 GN=Amuc_1074 PE=3 SV=1	Amuc_1074	5	5	10.10	62.82	552	18.05	5.E+08	74	2.E+07
B2UN71	tr|B2UN71|B2UN71_AKKM8 Glycoside hydrolase family 57 OS=Akkermansia muciniphila (strain ATCC BAA-835 / DSM 22959 / JCM 33894 / BCRC 81048 / CCUG 64013 / CIP 107961 / Muc) OX=349741 GN=Amuc_1868 PE=4 SV=1	Amuc_1868	5	5	15.70	45.10	395	13.43	3.E+08	14	2.E+07
B2UPB5	tr|B2UPB5|B2UPB5_AKKM8 Polysaccharide export protein OS=Akkermansia muciniphila (strain ATCC BAA-835 / DSM 22959 / JCM 33894 / BCRC 81048 / CCUG 64013 / CIP 107961 / Muc) OX=349741 GN=Amuc_2077 PE=4 SV=1	Amuc_2077	4	4	20.40	28.03	260	35.78	3.E+08	35	2.E+07
B2UM84	tr|B2UM84|B2UM84_AKKM8 Isoleucine--tRNA ligase OS=Akkermansia muciniphila (strain ATCC BAA-835 / DSM 22959 / JCM 33894 / BCRC 81048 / CCUG 64013 / CIP 107961 / Muc) OX=349741 GN=ileS PE=3 SV=1	Amuc_0189	12	12	16.80	102.39	909	47.91	9.E+08	189	2.E+07
B2UNL2	tr|B2UNL2|B2UNL2_AKKM8 Site-specific DNA-methyltransferase (adenine-specific) OS=Akkermansia muciniphila (strain ATCC BAA-835 / DSM 22959 / JCM 33894 / BCRC 81048 / CCUG 64013 / CIP 107961 / Muc) OX=349741 GN=Amuc_1915 PE=3 SV=1	Amuc_1915	12	12	15.00	95.77	853	40.70	8.E+08	86	2.E+07
B2ULS7	tr|B2ULS7|B2ULS7_AKKM8 Transposase IS3/IS911 family protein OS=Akkermansia muciniphila (strain ATCC BAA-835 / DSM 22959 / JCM 33894 / BCRC 81048 / CCUG 64013 / CIP 107961 / Muc) OX=349741 GN=Amuc_0127 PE=4 SV=1	Amuc_0127	3	3	31.70	13.95	126	16.53	1.E+08	9	2.E+07
B2URJ0	sp|B2URJ0|CARA_AKKM8 Carbamoyl-phosphate synthase small chain OS=Akkermansia muciniphila (strain ATCC BAA-835 / DSM 22959 / JCM 33894 / BCRC 81048 / CCUG 64013 / CIP 107961 / Muc) OX=349741 GN=carA PE=3 SV=1	Amuc_1250	4	4	17.70	40.19	372	13.16	2.E+08	34	2.E+07
B2UPP9	tr|B2UPP9|B2UPP9_AKKM8 Nitrilase/cyanide hydratase and apolipoprotein N-acyltransferase OS=Akkermansia muciniphila (strain ATCC BAA-835 / DSM 22959 / JCM 33894 / BCRC 81048 / CCUG 64013 / CIP 107961 / Muc) OX=349741 GN=Amuc_2118 PE=4 SV=1	Amuc_2118	3	3	11.90	32.04	285	14.89	2.E+08	23	2.E+07
B2UL26	tr|B2UL26|B2UL26_AKKM8 4-hydroxythreonine-4-phosphate dehydrogenase OS=Akkermansia muciniphila (strain ATCC BAA-835 / DSM 22959 / JCM 33894 / BCRC 81048 / CCUG 64013 / CIP 107961 / Muc) OX=349741 GN=Amuc_1477 PE=4 SV=1	Amuc_1477	3	3	17.30	31.16	289	16.71	2.E+08	27	2.E+07
B2UPT2;B2UQS7	tr|B2UPT2|B2UPT2_AKKM8 YD repeat protein OS=Akkermansia muciniphila (strain ATCC BAA-835 / DSM 22959 / JCM 33894 / BCRC 81048 / CCUG 64013 / CIP 107961 / Muc) OX=349741 GN=Amuc_2152 PE=4 SV=1;tr|B2UQS7|B2UQS7_AKKM8 YD repeat protein OS=Akkermansia muciniph	Amuc_2152	19	19	14.20	208.44	1912;1929	76.75	1.E+09	112	2.E+07
B2UPC8	tr|B2UPC8|B2UPC8_AKKM8 Thioesterase superfamily protein OS=Akkermansia muciniphila (strain ATCC BAA-835 / DSM 22959 / JCM 33894 / BCRC 81048 / CCUG 64013 / CIP 107961 / Muc) OX=349741 GN=Amuc_0568 PE=3 SV=1	Amuc_0568	2	2	18.90	16.61	143	14.15	1.E+08	6	1.E+07
B2UM07	tr|B2UM07|B2UM07_AKKM8 Carboxyl-terminal protease OS=Akkermansia muciniphila (strain ATCC BAA-835 / DSM 22959 / JCM 33894 / BCRC 81048 / CCUG 64013 / CIP 107961 / Muc) OX=349741 GN=Amuc_1631 PE=3 SV=1	Amuc_1631	8	8	13.60	83.86	748	47.61	4.E+08	31	1.E+07
B2UP66	tr|B2UP66|B2UP66_AKKM8 Dihydroorotase OS=Akkermansia muciniphila (strain ATCC BAA-835 / DSM 22959 / JCM 33894 / BCRC 81048 / CCUG 64013 / CIP 107961 / Muc) OX=349741 GN=pyrC PE=3 SV=1	Amuc_2027	5	5	16.80	36.40	333	11.33	2.E+08	14	1.E+07
B2UN04	tr|B2UN04|B2UN04_AKKM8 Uncharacterized protein OS=Akkermansia muciniphila (strain ATCC BAA-835 / DSM 22959 / JCM 33894 / BCRC 81048 / CCUG 64013 / CIP 107961 / Muc) OX=349741 GN=Amuc_1797 PE=4 SV=1	Amuc_1797	2	2	9.70	33.10	290	6.38	2.E+08	33	1.E+07
B2UR35	tr|B2UR35|B2UR35_AKKM8 ROK family protein OS=Akkermansia muciniphila (strain ATCC BAA-835 / DSM 22959 / JCM 33894 / BCRC 81048 / CCUG 64013 / CIP 107961 / Muc) OX=349741 GN=Amuc_1094 PE=4 SV=1	Amuc_1094	2	2	8.70	35.76	332	12.46	2.E+08	34	1.E+07
B2UMD2	tr|B2UMD2|B2UMD2_AKKM8 Beta-lactamase domain protein OS=Akkermansia muciniphila (strain ATCC BAA-835 / DSM 22959 / JCM 33894 / BCRC 81048 / CCUG 64013 / CIP 107961 / Muc) OX=349741 GN=Amuc_0238 PE=4 SV=1	Amuc_0238	5	5	13.00	52.42	468	14.04	3.E+08	43	1.E+07

B2ULD1	tr|B2ULD1|B2ULD1_AKKM8 PfkB domain protein OS=Akkermansia muciniphila (strain ATCC BAA-835 / DSM 22959 / JCM 33894 / BCRC 81048 / CCUG 64013 / CIP 107961 / Muc) OX=349741 GN=Amuc_0075 PE=4 SV=1	Amuc_0075	3	3	11.80	39.38	355	15.84	2.E+08	7	1.E+07
B2UQN1	tr|B2UQN1|B2UQN1_AKKM8 Adenylate kinase OS=Akkermansia muciniphila (strain ATCC BAA-835 / DSM 22959 / JCM 33894 / BCRC 81048 / CCUG 64013 / CIP 107961 / Muc) OX=349741 GN=adk PE=3 SV=1	Amuc_0934	2	2	12.00	24.69	216	8.73	1.E+08	22	1.E+07
B2URF3	tr|B2URF3|B2URF3_AKKM8 Uncharacterized protein OS=Akkermansia muciniphila (strain ATCC BAA-835 / DSM 22959 / JCM 33894 / BCRC 81048 / CCUG 64013 / CIP 107961 / Muc) OX=349741 GN=Amuc_1213 PE=4 SV=1	Amuc_1213	2	2	19.70	16.13	147	5.45	7.E+07	2	9.E+06
B2UL75	sp|B2UL75|G1091_AKKM8 Glycosyl hydrolase family 109 protein 1 OS=Akkermansia muciniphila (strain ATCC BAA-835 / DSM 22959 / JCM 33894 / BCRC 81048 / CCUG 64013 / CIP 107961 / Muc) OX=349741 GN=Amuc_0017 PE=3 SV=1	Amuc_0017	4	4	11.40	53.80	481	43.96	2.E+08	14	9.E+06
B2UQ57	tr|B2UQ57|B2UQ57_AKKM8 ADP-L-glycero-D-manno-heptose-6-epimerase OS=Akkermansia muciniphila (strain ATCC BAA-835 / DSM 22959 / JCM 33894 / BCRC 81048 / CCUG 64013 / CIP 107961 / Muc) OX=349741 GN=hldD PE=3 SV=1	Amuc_0756	4	4	16.20	36.86	328	14.77	2.E+08	13	9.E+06
B2UNI9	tr|B2UNI9|B2UNI9_AKKM8 Phosphopantothenoylcysteine decarboxylase OS=Akkermansia muciniphila (strain ATCC BAA-835 / DSM 22959 / JCM 33894 / BCRC 81048 / CCUG 64013 / CIP 107961 / Muc) OX=349741 GN=Amuc_0463 PE=4 SV=1	Amuc_0463	2	2	14.00	19.18	179	7.73	6.E+07	13	9.E+06
B2ULS2	tr|B2ULS2|B2ULS2_AKKM8 Sulfatase OS=Akkermansia muciniphila (strain ATCC BAA-835 / DSM 22959 / JCM 33894 / BCRC 81048 / CCUG 64013 / CIP 107961 / Muc) OX=349741 GN=Amuc_0121 PE=3 SV=1	Amuc_0121	6	6	16.20	57.46	526	22.83	3.E+08	36	9.E+06
B2UN08	tr|B2UN08|B2UN08_AKKM8 Peptidase S15 OS=Akkermansia muciniphila (strain ATCC BAA-835 / DSM 22959 / JCM 33894 / BCRC 81048 / CCUG 64013 / CIP 107961 / Muc) OX=349741 GN=Amuc_1801 PE=4 SV=1	Amuc_1801	5	5	20.10	39.75	359	23.41	2.E+08	18	9.E+06
B2UN18	tr|B2UN18|B2UN18_AKKM8 Uncharacterized protein OS=Akkermansia muciniphila (strain ATCC BAA-835 / DSM 22959 / JCM 33894 / BCRC 81048 / CCUG 64013 / CIP 107961 / Muc) OX=349741 GN=Amuc_1811 PE=4 SV=1	Amuc_1811	3	3	22.00	17.22	150	9.25	8.E+07	15	9.E+06
B2UKV7	tr|B2UKV7|B2UKV7_AKKM8 10 kDa chaperonin OS=Akkermansia muciniphila (strain ATCC BAA-835 / DSM 22959 / JCM 33894 / BCRC 81048 / CCUG 64013 / CIP 107961 / Muc) OX=349741 GN=groS PE=3 SV=1	Amuc_1407	2	2	29.20	10.31	96	7.25	4.E+07	3	8.E+06
B2UQQ5	tr|B2UQQ5|B2UQQ5_AKKM8 Exodeoxyribonuclease III OS=Akkermansia muciniphila (strain ATCC BAA-835 / DSM 22959 / JCM 33894 / BCRC 81048 / CCUG 64013 / CIP 107961 / Muc) OX=349741 GN=Amuc_0958 PE=3 SV=1	Amuc_0958	2	2	7.10	28.58	253	6.92	1.E+08	18	8.E+06
B2UKW1	tr|B2UKW1|B2UKW1_AKKM8 Uncharacterized protein OS=Akkermansia muciniphila (strain ATCC BAA-835 / DSM 22959 / JCM 33894 / BCRC 81048 / CCUG 64013 / CIP 107961 / Muc) OX=349741 GN=Amuc_1411 PE=4 SV=1	Amuc_1411	2	2	16.10	21.91	224	5.25	4.E+07	2	7.E+06
B2UMT3	sp|B2UMT3|RS3_AKKM8 30S ribosomal protein S3 OS=Akkermansia muciniphila (strain ATCC BAA-835 / DSM 22959 / JCM 33894 / BCRC 81048 / CCUG 64013 / CIP 107961 / Muc) OX=349741 GN=rpsC PE=3 SV=1	Amuc_0297	3	3	18.80	25.87	229	19.13	1.E+08	29	7.E+06
B2UNL1	tr|B2UNL1|B2UNL1_AKKM8 Restriction modification system DNA specificity domain OS=Akkermansia muciniphila (strain ATCC BAA-835 / DSM 22959 / JCM 33894 / BCRC 81048 / CCUG 64013 / CIP 107961 / Muc) OX=349741 GN=Amuc_1914 PE=4 SV=1	Amuc_1914	2	2	9.80	43.90	386	11.72	1.E+08	12	6.E+06
B2URC9	sp|B2URC9|RL28_AKKM8 50S ribosomal protein L28 OS=Akkermansia muciniphila (strain ATCC BAA-835 / DSM 22959 / JCM 33894 / BCRC 81048 / CCUG 64013 / CIP 107961 / Muc) OX=349741 GN=rpmB PE=3 SV=1	Amuc_1189	2	2	23.00	9.85	87	4.41	2.E+07	3	6.E+06
B2UKX6	tr|B2UKX6|B2UKX6_AKKM8 Histidinol-phosphatase OS=Akkermansia muciniphila (strain ATCC BAA-835 / DSM 22959 / JCM 33894 / BCRC 81048 / CCUG 64013 / CIP 107961 / Muc) OX=349741 GN=Amuc_1427 PE=3 SV=1	Amuc_1427	2	2	6.30	30.87	269	2.45	7.E+07	2	5.E+06
B2UQK6	tr|B2UQK6|B2UQK6_AKKM8 3-phosphate/5-hydroxy nucleic acid ligase OS=Akkermansia muciniphila (strain ATCC BAA-835 / DSM 22959 / JCM 33894 / BCRC 81048 / CCUG 64013 / CIP 107961 / Muc) OX=349741 GN=Amuc_0909 PE=3 SV=1	Amuc_0909	4	4	13.70	41.25	371	8.23	1.E+08	10	5.E+06
B2UNM5	tr|B2UNM5|B2UNM5_AKKM8 Periplasmic binding protein OS=Akkermansia muciniphila (strain ATCC BAA-835 / DSM 22959 / JCM 33894 / BCRC 81048 / CCUG 64013 / CIP 107961 / Muc) OX=349741 GN=Amuc_1928 PE=4 SV=1	Amuc_1928	3	3	14.00	32.90	307	6.88	1.E+08	22	5.E+06

B2URM0	tr|B2URM0|B2URM0_AKKM8 Aminopeptidase OS=Akkermansia muciniphila (strain ATCC BAA-835 / DSM 22959 / JCM 33894 / BCRC 81048 / CCUG 64013 / CIP 107961 / Muc) OX=349741 GN=Amuc_1280 PE=4 SV=1	Amuc_1280	2	2	8.40	40.49	369	7.98	9.E+07	5	5.E+06
B2UQ85	tr|B2UQ85|B2UQ85_AKKM8 FAD dependent oxidoreductase OS=Akkermansia muciniphila (strain ATCC BAA-835 / DSM 22959 / JCM 33894 / BCRC 81048 / CCUG 64013 / CIP 107961 / Muc) OX=349741 GN=Amuc_0786 PE=4 SV=1	Amuc_0786	2	2	10.10	38.46	346	6.14	9.E+07	17	5.E+06
B2UPK9	tr|B2UPK9|B2UPK9_AKKM8 Transcriptional regulator MraZ OS=Akkermansia muciniphila (strain ATCC BAA-835 / DSM 22959 / JCM 33894 / BCRC 81048 / CCUG 64013 / CIP 107961 / Muc) OX=349741 GN=mraZ PE=3 SV=1	Amuc_0649	3	3	24.50	17.05	151	5.24	5.E+07	9	5.E+06
B2UPI5	tr|B2UPI5|B2UPI5_AKKM8 Exo-alpha-sialidase OS=Akkermansia muciniphila (strain ATCC BAA-835 / DSM 22959 / JCM 33894 / BCRC 81048 / CCUG 64013 / CIP 107961 / Muc) OX=349741 GN=Amuc_0625 PE=4 SV=1	Amuc_0625	2	2	5.70	45.59	419	3.33	1.E+08	6	5.E+06
B2UQH0	tr|B2UQH0|B2UQH0_AKKM8 Acetylornithine and succinylornithine aminotransferase OS=Akkermansia muciniphila (strain ATCC BAA-835 / DSM 22959 / JCM 33894 / BCRC 81048 / CCUG 64013 / CIP 107961 / Muc) OX=349741 GN=Amuc_0872 PE=3 SV=1	Amuc_0872	4	4	12.20	45.36	418	12.23	1.E+08	23	5.E+06
B2URI8	tr|B2URI8|B2URI8_AKKM8 GDP-L-fucose synthase OS=Akkermansia muciniphila (strain ATCC BAA-835 / DSM 22959 / JCM 33894 / BCRC 81048 / CCUG 64013 / CIP 107961 / Muc) OX=349741 GN=fcl PE=3 SV=1	Amuc_1248	3	3	13.50	40.65	362	10.52	7.E+07	12	4.E+06
B2URJ3	tr|B2URJ3|B2URJ3_AKKM8 Glutamate synthase (Ferredoxin) OS=Akkermansia muciniphila (strain ATCC BAA-835 / DSM 22959 / JCM 33894 / BCRC 81048 / CCUG 64013 / CIP 107961 / Muc) OX=349741 GN=Amuc_1253 PE=3 SV=1	Amuc_1253	6	6	5.70	161.75	1479	44.35	2.E+08	17	4.E+06
B2URE6	tr|B2URE6|B2URE6_AKKM8 Bifunctional purine biosynthesis protein PurH OS=Akkermansia muciniphila (strain ATCC BAA-835 / DSM 22959 / JCM 33894 / BCRC 81048 / CCUG 64013 / CIP 107961 / Muc) OX=349741 GN=purH PE=3 SV=1	Amuc_1206	2	2	4.50	56.36	515	5.65	1.E+08	16	3.E+06
B2UL15	tr|B2UL15|B2UL15_AKKM8 FAD linked oxidase domain protein OS=Akkermansia muciniphila (strain ATCC BAA-835 / DSM 22959 / JCM 33894 / BCRC 81048 / CCUG 64013 / CIP 107961 / Muc) OX=349741 GN=Amuc_1466 PE=4 SV=1	Amuc_1466	4	4	12.90	48.88	458	8.03	8.E+07	10	3.E+06
B2UMH6	tr|B2UMH6|B2UMH6_AKKM8 Histidinol dehydrogenase OS=Akkermansia muciniphila (strain ATCC BAA-835 / DSM 22959 / JCM 33894 / BCRC 81048 / CCUG 64013 / CIP 107961 / Muc) OX=349741 GN=Amuc_1714 PE=3 SV=1	Amuc_1714	2	2	5.30	46.29	432	8.97	6.E+07	7	3.E+06
B2UKT9	sp|B2UKT9|ISPG_AKKM8 4-hydroxy-3-methylbut-2-en-1-yl diphosphate synthase (flavodoxin) OS=Akkermansia muciniphila (strain ATCC BAA-835 / DSM 22959 / JCM 33894 / BCRC 81048 / CCUG 64013 / CIP 107961 / Muc) OX=349741 GN=ispG PE=3 SV=1	Amuc_1388	5	5	13.20	64.49	585	15.11	1.E+08	26	3.E+06
B2ULH3	tr|B2ULH3|B2ULH3_AKKM8 Homoserine O-acetyltransferase OS=Akkermansia muciniphila (strain ATCC BAA-835 / DSM 22959 / JCM 33894 / BCRC 81048 / CCUG 64013 / CIP 107961 / Muc) OX=349741 GN=metXA PE=3 SV=1	Amuc_1539	2	2	7.50	42.86	387	13.92	6.E+07	21	3.E+06
B2UQU9	tr|B2UQU9|B2UQU9_AKKM8 Glycoside hydrolase family 31 OS=Akkermansia muciniphila (strain ATCC BAA-835 / DSM 22959 / JCM 33894 / BCRC 81048 / CCUG 64013 / CIP 107961 / Muc) OX=349741 GN=Amuc_1008 PE=3 SV=1	Amuc_1008	4	4	5.30	146.26	1311	11.39	2.E+08	15	3.E+06
B2UND5	tr|B2UND5|B2UND5_AKKM8 Putative substrate-binding protein of aliphatic sulfonate ABC transporter OS=Akkermansia muciniphila (strain ATCC BAA-835 / DSM 22959 / JCM 33894 / BCRC 81048 / CCUG 64013 / CIP 107961 / Muc) OX=349741 GN=Amuc_0409 PE=4 SV=1	Amuc_0409	2	2	8.30	38.40	348	3.16	5.E+07	13	3.E+06
B2UQE7	sp|B2UQE7|MIAB_AKKM8 tRNA-2-methylthio-N(6)-dimethylallyladenosine synthase OS=Akkermansia muciniphila (strain ATCC BAA-835 / DSM 22959 / JCM 33894 / BCRC 81048 / CCUG 64013 / CIP 107961 / Muc) OX=349741 GN=miaB PE=3 SV=1	Amuc_0849	2	2	6.80	51.96	458	6.71	7.E+07	16	3.E+06
B2ULC6	tr|B2ULC6|B2ULC6_AKKM8 Tryptophan--tRNA ligase OS=Akkermansia muciniphila (strain ATCC BAA-835 / DSM 22959 / JCM 33894 / BCRC 81048 / CCUG 64013 / CIP 107961 / Muc) OX=349741 GN=trpS PE=3 SV=1	Amuc_0070	2	2	10.00	35.86	321	6.09	4.E+07	3	2.E+06
B2UQX2	tr|B2UQX2|B2UQX2_AKKM8 Cysteine--tRNA ligase OS=Akkermansia muciniphila (strain ATCC BAA-835 / DSM 22959 / JCM 33894 / BCRC 81048 / CCUG 64013 / CIP 107961 / Muc) OX=349741 GN=cysS PE=3 SV=1	Amuc_1031	3	3	7.00	53.02	471	8.35	5.E+07	16	2.E+06

B2UN50	tr|B2UN50|B2UN50_AKKM8 PDZ/DHR/GLGF domain protein OS=Akkermansia muciniphila (strain ATCC BAA-835 / DSM 22959 / JCM 33894 / BCRC 81048 / CCUG 64013 / CIP 107961 / Muc) OX=349741 GN=Amuc_1844 PE=4 SV=1	Amuc_1844	2	2	10.00	34.08	319	5.79	2.E+07	3	1.E+06
B2ULN0	tr|B2ULN0|B2ULN0_AKKM8 Polyprenyl synthetase OS=Akkermansia muciniphila (strain ATCC BAA-835 / DSM 22959 / JCM 33894 / BCRC 81048 / CCUG 64013 / CIP 107961 / Muc) OX=349741 GN=Amuc_1597 PE=3 SV=1	Amuc_1597	2	2	5.70	37.33	336	2.80	2.E+07	10	1.E+06
B2UQJ6	tr|B2UQJ6|B2UQJ6_AKKM8 DNA topoisomerase OS=Akkermansia muciniphila (strain ATCC BAA-835 / DSM 22959 / JCM 33894 / BCRC 81048 / CCUG 64013 / CIP 107961 / Muc) OX=349741 GN=Amuc_0898 PE=3 SV=1	Amuc_0898	4	4	4.90	97.19	869	7.12	5.E+07	8	9.E+05
B2ULZ9	sp|B2ULZ9|RIMO_AKKM8 Ribosomal protein S12 methylthiotransferase RimO OS=Akkermansia muciniphila (strain ATCC BAA-835 / DSM 22959 / JCM 33894 / BCRC 81048 / CCUG 64013 / CIP 107961 / Muc) OX=349741 GN=rimO PE=3 SV=1	Amuc_1623	4	4	10.00	50.86	452	5.59	2.E+07	5	9.E+05
B2UQE3	sp|B2UQE3|END4_AKKM8 Probable endonuclease 4 OS=Akkermansia muciniphila (strain ATCC BAA-835 / DSM 22959 / JCM 33894 / BCRC 81048 / CCUG 64013 / CIP 107961 / Muc) OX=349741 GN=nfo PE=3 SV=1	Amuc_0845	3	3	14.10	30.78	277	4.26	1.E+07	1	8.E+05
B2UN91	sp|B2UN91|SYE_AKKM8 Glutamate--tRNA ligase OS=Akkermansia muciniphila (strain ATCC BAA-835 / DSM 22959 / JCM 33894 / BCRC 81048 / CCUG 64013 / CIP 107961 / Muc) OX=349741 GN=gltX PE=3 SV=1	Amuc_1888	2	2	7.60	48.61	433	6.33	2.E+07	7	8.E+05
B2UNY2	tr|B2UNY2|B2UNY2_AKKM8 Peptidoglycan glycosyltransferase OS=Akkermansia muciniphila (strain ATCC BAA-835 / DSM 22959 / JCM 33894 / BCRC 81048 / CCUG 64013 / CIP 107961 / Muc) OX=349741 GN=Amuc_0514 PE=4 SV=1	Amuc_0514	2	2	4.10	89.28	822	7.04	3.E+07	3	7.E+05
B2UR84	tr|B2UR84|B2UR84_AKKM8 YD repeat protein OS=Akkermansia muciniphila (strain ATCC BAA-835 / DSM 22959 / JCM 33894 / BCRC 81048 / CCUG 64013 / CIP 107961 / Muc) OX=349741 GN=Amuc_1143 PE=4 SV=1	Amuc_1143	3	3	2.40	217.62	1991	2.39	6.E+07	4	7.E+05
B2UKU3	tr|B2UKU3|B2UKU3_AKKM8 Beta-ketoacyl synthase OS=Akkermansia muciniphila (strain ATCC BAA-835 / DSM 22959 / JCM 33894 / BCRC 81048 / CCUG 64013 / CIP 107961 / Muc) OX=349741 GN=Amuc_1392 PE=3 SV=1	Amuc_1392	2	2	6.40	40.31	390	2.93	4.E+06	1	2.E+05
B2URL5	tr|B2URL5|B2URL5_AKKM8 Uncharacterized protein OS=Akkermansia muciniphila (strain ATCC BAA-835 / DSM 22959 / JCM 33894 / BCRC 81048 / CCUG 64013 / CIP 107961 / Muc) OX=349741 GN=Amuc_1275 PE=4 SV=1	Amuc_1275	2	2	6.60	38.86	348	3.84	2.E+06	2	1.E+05
B2UL46	tr|B2UL46|B2UL46_AKKM8 Uncharacterized protein OS=Akkermansia muciniphila (strain ATCC BAA-835 / DSM 22959 / JCM 33894 / BCRC 81048 / CCUG 64013 / CIP 107961 / Muc) OX=349741 GN=Amuc_1497 PE=4 SV=1	Amuc_1497	2	2	6.60	38.92	349	2.63	0.E+00	2	0.E+00

Supplementary Table 3. List of signal peptide-containing proteins identified in secretome of *A. muciniphila* cultivated under basal medium.

Majority protein IDs	Fasta headers	Gene locus	Peptides	Unique peptides	Sequence coverage [%]	Mol. weight [kDa]	Score	Intensity	MS/MS count	iBAQ	SignalP		Secretoem P (Probability)	PSORTb 3.0 (subcellular localization)
											Signal peptide	Probability		
B2UMR7	tr|B2UMR7|B2UMR7_AKKM8 Histone family protein DNA-binding protein OS=Akkermansia muciniphila (strain ATCC BAA-835 / DSM 22959 / JCM 33894 / BCRC 81048 / CCUG 64013 / CIP 107961 / Muc) OX=349741 GN=Amuc_0281 PE=3 SV=1	Amuc_0281	4	4	50.50	11.25	210.98	6.E+10	587	2.E+10	-	-	0.540	Cytoplasm
B2URF0	tr|B2URF0|B2URF0_AKKM8 Phosphoenolpyruvate carboxykinase [GTP] OS=Akkermansia muciniphila (strain ATCC BAA-835 / DSM 22959 / JCM 33894 / BCRC 81048 / CCUG 64013 / CIP 107961 / Muc) OX=349741 GN=pckG PE=3 SV=1	Amuc_1210	22	22	48.40	67.52	323.31	2.E+11	2108	8.E+09	-	-	0.817	Cytoplasm
B2UKV9	tr|B2UKV9|B2UKV9_AKKM8 PepSY_like domain-containing protein OS=Akkermansia muciniphila (strain ATCC BAA-835 / DSM 22959 / JCM 33894 / BCRC 81048 / CCUG 64013 / CIP 107961 / Muc) OX=349741 GN=Amuc_1409 PE=4 SV=1	Amuc_1409	8	8	52.00	16.52	245.16	4.E+10	538	5.E+09	Signal peptide (Sec/SPI)	0.998	-	Unknown
B2UL96	sp|B2UL96|GLSA_AKKM8 Glutaminase OS=Akkermansia muciniphila (strain ATCC BAA-835 / DSM 22959 / JCM 33894 / BCRC 81048 / CCUG 64013 / CIP 107961 / Muc) OX=349741 GN=glsA PE=3 SV=1	Amuc_0038	7	7	31.00	33.13	186.41	3.E+10	391	3.E+09	-	-	0.875	Cytoplasm
B2UKY5	sp|B2UKY5|MDH_AKKM8 Malate dehydrogenase OS=Akkermansia muciniphila (strain ATCC BAA-835 / DSM 22959 / JCM 33894 / BCRC 81048 / CCUG 64013 / CIP 107961 / Muc) OX=349741 GN=mdh PE=3 SV=1	Amuc_1436	13	13	47.10	35.10	323.31	4.E+10	548	2.E+09	-	-	0.571	Cytoplasm
B2UQW7	tr|B2UQW7|B2UQW7_AKKM8 Peptidyl-prolyl cis-trans isomerase OS=Akkermansia muciniphila (strain ATCC BAA-835 / DSM 22959 / JCM 33894 / BCRC 81048 / CCUG 64013 / CIP 107961 / Muc) OX=349741 GN=Amuc_1026 PE=3 SV=1	Amuc_1026	5	5	47.90	17.92	260.88	6.E+09	192	8.E+08	-	-	0.888	Cytoplasm
B2UMX5	tr|B2UMX5|B2UMX5_AKKM8 OsmC family protein OS=Akkermansia muciniphila (strain ATCC BAA-835 / DSM 22959 / JCM 33894 / BCRC 81048 / CCUG 64013 / CIP 107961 / Muc) OX=349741 GN=Amuc_0342 PE=4 SV=1	Amuc_0342	7	7	65.20	14.48	113.32	5.E+09	262	8.E+08	-	-	0.660	Unknown
B2UPJ1	tr|B2UPJ1|B2UPJ1_AKKM8 FAD-binding domain protein OS=Akkermansia muciniphila (strain ATCC BAA-835 / DSM 22959 / JCM 33894 / BCRC 81048 / CCUG 64013 / CIP 107961 / Muc) OX=349741 GN=Amuc_0631 PE=4 SV=1	Amuc_0631	13	13	53.50	41.44	323.31	1.E+10	521	7.E+08	-	-	0.614	Cytoplasmic Membrane
B2UP16	tr|B2UP16|B2UP16_AKKM8 MotA/TolQ/ExbB proton channel OS=Akkermansia muciniphila (strain ATCC BAA-835 / DSM 22959 / JCM 33894 / BCRC 81048 / CCUG 64013 / CIP 107961 / Muc) OX=349741 GN=Amuc_0548 PE=3 SV=1	Amuc_0548	2	2	11.80	28.34	12.58	3.E+09	44	6.E+08	Signal peptide (Sec/SPI)	0.964	-	Cytoplasmic Membrane

B2UNP3	tr|B2UNP3|B2UNP3_AKKM8 Dihydrodipicolinate synthetase OS=Akkermansia muciniphila (strain ATCC BAA-835 / DSM 22959 / JCM 33894 / BCRC 81048 / CCUG 64013 / CIP 107961 / Muc) OX=349741 GN=Amuc_1946 PE=3 SV=1	Amuc_1946	6	6	25.60	33.03	119.27	8.E+09	463	5.E+08	-	-	0.881	Cytoplasm
B2UQK1	tr|B2UQK1|B2UQK1_AKKM8 Aconitate hydratase OS=Akkermansia muciniphila (strain ATCC BAA-835 / DSM 22959 / JCM 33894 / BCRC 81048 / CCUG 64013 / CIP 107961 / Muc) OX=349741 GN=Amuc_0904 PE=3 SV=1	Amuc_0904	23	23	38.40	99.01	323.31	2.E+10	1077	4.E+08	-	-	0.575	Cytoplasm
B2UKP3	tr|B2UKP3|B2UKP3_AKKM8 Uncharacterized protein OS=Akkermansia muciniphila (strain ATCC BAA-835 / DSM 22959 / JCM 33894 / BCRC 81048 / CCUG 64013 / CIP 107961 / Muc) OX=349741 GN=Amuc_1342 PE=4 SV=1	Amuc_1342	4	4	17.30	34.98	90.38	5.E+09	235	3.E+08	-	-	0.743	Cytoplasm
B2UMM9	tr|B2UMM9|B2UMM9_AKKM8 Uncharacterized protein OS=Akkermansia muciniphila (strain ATCC BAA-835 / DSM 22959 / JCM 33894 / BCRC 81048 / CCUG 64013 / CIP 107961 / Muc) OX=349741 GN=Amuc_1767 PE=4 SV=1	Amuc_1767	11	11	47.60	32.22	317.01	4.E+09	236	2.E+08	Signal peptide (Sec/SPI)	0.982	-	Unknown
B2UP96	tr|B2UP96|B2UP96_AKKM8 Thioredoxin domain-containing protein OS=Akkermansia muciniphila (strain ATCC BAA-835 / DSM 22959 / JCM 33894 / BCRC 81048 / CCUG 64013 / CIP 107961 / Muc) OX=349741 GN=Amuc_2057 PE=4 SV=1	Amuc_2057	4	4	29.30	18.12	48.08	2.E+09	144	2.E+08	Signal peptide (Sec/SPI)	0.936	0.934	Unknown
B2ULU7	tr|B2ULU7|B2ULU7_AKKM8 Thioredoxin domain OS=Akkermansia muciniphila (strain ATCC BAA-835 / DSM 22959 / JCM 33894 / BCRC 81048 / CCUG 64013 / CIP 107961 / Muc) OX=349741 GN=Amuc_0147 PE=4 SV=1	Amuc_0147	9	9	29.60	34.97	86.17	4.E+09	274	2.E+08	Signal peptide (Sec/SPI)	0.981	0.914	Unknown
B2UPY9	tr|B2UPY9|B2UPY9_AKKM8 Outer membrane autotransporter barrel domain protein OS=Akkermansia muciniphila (strain ATCC BAA-835 / DSM 22959 / JCM 33894 / BCRC 81048 / CCUG 64013 / CIP 107961 / Muc) OX=349741 GN=Amuc_0687 PE=4 SV=1	Amuc_0687	13	13	27.30	107.54	323.31	8.E+09	284	2.E+08	Signal peptide (Sec/SPI)	0.996	0.950	OuterMembrane
B2ULC0	tr|B2ULC0|B2ULC0_AKKM8 5-oxopent-3-ene-1,2,5-tricarboxylate decarboxylase OS=Akkermansia muciniphila (strain ATCC BAA-835 / DSM 22959 / JCM 33894 / BCRC 81048 / CCUG 64013 / CIP 107961 / Muc) OX=349741 GN=Amuc_0064 PE=4 SV=1	Amuc_0064	7	7	36.00	31.33	130.07	3.E+09	226	2.E+08	-	-	0.763	Cytoplasm
B2UQ02	tr|B2UQ02|B2UQ02_AKKM8 Uncharacterized protein OS=Akkermansia muciniphila (strain ATCC BAA-835 / DSM 22959 / JCM 33894 / BCRC 81048 / CCUG 64013 / CIP 107961 / Muc) OX=349741 GN=Amuc_0700 PE=4 SV=1	Amuc_0700	2	2	13.70	15.12	51.76	5.E+08	26	2.E+08	Lipoprotein signal peptide (Sec/SPII)	0.999	0.934	Unknown
B2UPA8	tr|B2UPA8|B2UPA8_AKKM8 Catalase OS=Akkermansia muciniphila (strain ATCC BAA-835 / DSM 22959 / JCM 33894 / BCRC 81048 / CCUG 64013 / CIP 107961 / Muc) OX=349741 GN=Amuc_2070 PE=3 SV=1	Amuc_2070	17	17	29.60	84.03	146.98	4.E+09	296	1.E+08	-	-	0.842	Cytoplasm

B2UQP6	tr|B2UQP6|B2UQP6_AKKM8 Peptidyl-prolyl cis-trans isomerase OS=Akkermansia muciniphila (strain ATCC BAA-835 / DSM 22959 / JCM 33894 / BCRC 81048 / CCUG 64013 / CIP 107961 / Muc) OX=349741 GN=Amuc_0949 PE=3 SV=1	Amuc_0949	2	2	12.70	20.97	47.03	6.E+08	127	1.E+08	Signal peptide (Sec/SPI)	0.807	0.786	Unknown
B2UP74	tr|B2UP74|B2UP74_AKKM8 Uncharacterized protein OS=Akkermansia muciniphila (strain ATCC BAA-835 / DSM 22959 / JCM 33894 / BCRC 81048 / CCUG 64013 / CIP 107961 / Muc) OX=349741 GN=Amuc_2035 PE=4 SV=1	Amuc_2035	4	4	22.30	27.43	116.43	1.E+09	59	9.E+07	-	-	0.891	Periplasmic space
B2UPW3	tr|B2UPW3|B2UPW3_AKKM8 D-alanine--D-alanine ligase OS=Akkermansia muciniphila (strain ATCC BAA-835 / DSM 22959 / JCM 33894 / BCRC 81048 / CCUG 64013 / CIP 107961 / Muc) OX=349741 GN=ddl PE=3 SV=1	Amuc_0661	3	3	10.30	33.30	23.44	1.E+09	89	7.E+07	-	-	0.515	Cytoplasm
B2UKP4	tr|B2UKP4|B2UKP4_AKKM8 Uncharacterized protein OS=Akkermansia muciniphila (strain ATCC BAA-835 / DSM 22959 / JCM 33894 / BCRC 81048 / CCUG 64013 / CIP 107961 / Muc) OX=349741 GN=Amuc_1343 PE=4 SV=1	Amuc_1343	2	2	13.30	20.32	12.68	4.E+08	41	5.E+07	Signal peptide (Sec/SPI)	0.946	-	Cytoplasmic Membrane
B2UNZ3	tr|B2UNZ3|B2UNZ3_AKKM8 Cycloartenol synthase-like protein OS=Akkermansia muciniphila (strain ATCC BAA-835 / DSM 22959 / JCM 33894 / BCRC 81048 / CCUG 64013 / CIP 107961 / Muc) OX=349741 GN=Amuc_0525 PE=4 SV=1	Amuc_0525	8	8	30.40	43.36	42.09	1.E+09	69	5.E+07	Signal peptide (Sec/SPI)	0.967	0.936	Unknown
B2UKX4	tr|B2UKX4|B2UKX4_AKKM8 Kelch repeat-containing protein OS=Akkermansia muciniphila (strain ATCC BAA-835 / DSM 22959 / JCM 33894 / BCRC 81048 / CCUG 64013 / CIP 107961 / Muc) OX=349741 GN=Amuc_1425 PE=4 SV=1	Amuc_1425	2	2	9.20	35.13	29.39	7.E+08	56	5.E+07	-	-	0.910	Unknown
B2UQX4	tr|B2UQX4|B2UQX4_AKKM8 Sulfatase OS=Akkermansia muciniphila (strain ATCC BAA-835 / DSM 22959 / JCM 33894 / BCRC 81048 / CCUG 64013 / CIP 107961 / Muc) OX=349741 GN=Amuc_1033 PE=3 SV=1	Amuc_1033	9	9	18.20	67.00	42.47	2.E+09	103	5.E+07	Signal peptide (Sec/SPI)	0.745	0.922	Periplasmic space
B2UPD6	tr|B2UPD6|B2UPD6_AKKM8 Peptidase M16 domain protein OS=Akkermansia muciniphila (strain ATCC BAA-835 / DSM 22959 / JCM 33894 / BCRC 81048 / CCUG 64013 / CIP 107961 / Muc) OX=349741 GN=Amuc_0576 PE=3 SV=1	Amuc_0576	22	22	16.90	160.48	199.61	4.E+09	264	5.E+07	-	-	0.794	Unknown
B2UQ36	tr|B2UQ36|B2UQ36_AKKM8 YD repeat protein OS=Akkermansia muciniphila (strain ATCC BAA-835 / DSM 22959 / JCM 33894 / BCRC 81048 / CCUG 64013 / CIP 107961 / Muc) OX=349741 GN=Amuc_0735 PE=4 SV=1	Amuc_0735	23	23	19.20	212.82	161.17	4.E+09	172	4.E+07	-	-	0.932	Unknown
B2UQY5	tr|B2UQY5|B2UQY5_AKKM8 50S ribosomal protein L1 OS=Akkermansia muciniphila (strain ATCC BAA-835 / DSM 22959 / JCM 33894 / BCRC 81048 / CCUG 64013 / CIP 107961 / Muc) OX=349741 GN=rplA PE=3 SV=1	Amuc_1044	4	4	26.60	24.67	41.16	5.E+08	34	4.E+07	-	-	0.956	Cytoplasm
B2UQJ3	tr|B2UQJ3|B2UQJ3_AKKM8 PepSY_like domain-containing protein OS=Akkermansia muciniphila (strain ATCC BAA-835 / DSM 22959 / JCM 33894 / BCRC 81048 / CCUG	Amuc_0895	3	3	24.30	16.87	8.63	3.E+08	24	4.E+07	Signal peptide (Sec/SPI)	0.982	0.591	Unknown

	64013 / CIP 107961 / Muc) OX=349741 GN=Amuc_0895 PE=4 SV=1													
B2UQI1	tr|B2UQI1|B2UQI1_AKKM8 Uncharacterized protein OS=Akkermansia muciniphila (strain ATCC BAA-835 / DSM 22959 / JCM 33894 / BCRC 81048 / CCUG 64013 / CIP 107961 / Muc) OX=349741 GN=Amuc_0883 PE=4 SV=1	Amuc_0883	2	2	21.00	6.74	29.80	8.E+07	4	4.E+07	-	-	0.683	Unknown
B2UMH4	tr|B2UMH4|B2UMH4_AKKM8 Succinate--CoA ligase [ADP-forming] subunit alpha OS=Akkermansia muciniphila (strain ATCC BAA-835 / DSM 22959 / JCM 33894 / BCRC 81048 / CCUG 64013 / CIP 107961 / Muc) OX=349741 GN=sucD PE=3 SV=1	Amuc_1712	4	4	18.60	30.57	20.75	5.E+08	39	4.E+07	-	-	0.805	Cytoplasm
B2UQC2	tr|B2UQC2|B2UQC2_AKKM8 Lactase OS=Akkermansia muciniphila (strain ATCC BAA-835 / DSM 22959 / JCM 33894 / BCRC 81048 / CCUG 64013 / CIP 107961 / Muc) OX=349741 GN=Amuc_0824 PE=3 SV=1	Amuc_0824	20	20	18.00	142.05	124.73	2.E+09	273	3.E+07	Lipoprotein signal peptide (Sec/SPII)	0.712	0.936	Periplasmic space
B2URJ1	tr|B2URJ1|B2URJ1_AKKM8 Carbamoyl-phosphate synthase large chain OS=Akkermansia muciniphila (strain ATCC BAA-835 / DSM 22959 / JCM 33894 / BCRC 81048 / CCUG 64013 / CIP 107961 / Muc) OX=349741 GN=carB PE=3 SV=1	Amuc_1251	16	16	21.00	117.47	120.87	2.E+09	230	3.E+07	Lipoprotein signal peptide (Sec/SPII)	0.713	-	Unknown
B2UP25	sp|B2UP25|RS14_AKKM8 30S ribosomal protein S14 OS=Akkermansia muciniphila (strain ATCC BAA-835 / DSM 22959 / JCM 33894 / BCRC 81048 / CCUG 64013 / CIP 107961 / Muc) OX=349741 GN=rpsN PE=3 SV=1	Amuc_0557	2	2	25.80	10.28	7.50	6.E+07	15	3.E+07	-	-	0.568	Cytoplasm
B2UN14	tr|B2UN14|B2UN14_AKKM8 Aldo/keto reductase OS=Akkermansia muciniphila (strain ATCC BAA-835 / DSM 22959 / JCM 33894 / BCRC 81048 / CCUG 64013 / CIP 107961 / Muc) OX=349741 GN=Amuc_1807 PE=4 SV=1	Amuc_1807	7	7	33.40	34.57	25.97	5.E+08	86	3.E+07	Signal peptide (Sec/SPI)	0.507	-	Cytoplasm
B2UN11	tr|B2UN11|B2UN11_AKKM8 Flavodoxin-like domain-containing protein OS=Akkermansia muciniphila (strain ATCC BAA-835 / DSM 22959 / JCM 33894 / BCRC 81048 / CCUG 64013 / CIP 107961 / Muc) OX=349741 GN=Amuc_1804 PE=4 SV=1	Amuc_1804	2	2	14.00	19.22	8.61	3.E+08	16	3.E+07	-	-	0.813	Unknown
B2ULE0	tr|B2ULE0|B2ULE0_AKKM8 PA14 domain-containing protein OS=Akkermansia muciniphila (strain ATCC BAA-835 / DSM 22959 / JCM 33894 / BCRC 81048 / CCUG 64013 / CIP 107961 / Muc) OX=349741 GN=Amuc_0084 PE=4 SV=1	Amuc_0084	3	3	9.90	61.62	47.40	7.E+08	49	2.E+07	-	-	0.771	Cytoplasm
B2UL94	tr|B2UL94|B2UL94_AKKM8 YD repeat protein OS=Akkermansia muciniphila (strain ATCC BAA-835 / DSM 22959 / JCM 33894 / BCRC 81048 / CCUG 64013 / CIP 107961 / Muc) OX=349741 GN=Amuc_0036 PE=4 SV=1	Amuc_0036	26	26	19.70	214.69	147.05	2.E+09	211	2.E+07	-	-	0.937	Unknown
B2UR15	tr|B2UR15|B2UR15_AKKM8 Sulfatase OS=Akkermansia muciniphila (strain ATCC BAA-835 / DSM 22959 / JCM 33894 / BCRC 81048 / CCUG 64013 / CIP 107961 / Muc) OX=349741 GN=Amuc_1074 PE=3 SV=1	Amuc_1074	5	5	10.10	62.82	18.05	5.E+08	74	2.E+07	Signal peptide (Sec/SPI)	0.982	0.930	Periplasmic space

B2UPB5	tr|B2UPB5|B2UPB5_AKKM8 Polysaccharide export protein OS=Akkermansia muciniphila (strain ATCC BAA-835 / DSM 22959 / JCM 33894 / BCRC 81048 / CCUG 64013 / CIP 107961 / Muc) OX=349741 GN=Amuc_2077 PE=4 SV=1	Amuc_2077	4	4	20.40	28.03	35.78	3.E+08	35	2.E+07	Lipoprotein signal peptide (Sec/SPII)	0.960	0.726	Unknown
B2ULS7	tr|B2ULS7|B2ULS7_AKKM8 Transposase IS3/IS911 family protein OS=Akkermansia muciniphila (strain ATCC BAA-835 / DSM 22959 / JCM 33894 / BCRC 81048 / CCUG 64013 / CIP 107961 / Muc) OX=349741 GN=Amuc_0127 PE=4 SV=1	Amuc_0127	3	3	31.70	13.95	16.53	1.E+08	9	2.E+07	-	-	0.739	Unknown
B2UPT2;B2UQS7	tr|B2UPT2|B2UPT2_AKKM8 YD repeat protein OS=Akkermansia muciniphila (strain ATCC BAA-835 / DSM 22959 / JCM 33894 / BCRC 81048 / CCUG 64013 / CIP 107961 / Muc) OX=349741 GN=Amuc_2152 PE=4 SV=1;tr|B2UQS7|B2UQS7_AKKM8 YD repeat protein OS=Akkermansia muciniph	Amuc_2152	19	19	14.20	208.44	76.75	1.E+09	112	2.E+07	-	-	0.940	OuterMembrane
B2UM07	tr|B2UM07|B2UM07_AKKM8 Carboxyl-terminal protease OS=Akkermansia muciniphila (strain ATCC BAA-835 / DSM 22959 / JCM 33894 / BCRC 81048 / CCUG 64013 / CIP 107961 / Muc) OX=349741 GN=Amuc_1631 PE=3 SV=1	Amuc_1631	8	8	13.60	83.86	47.61	4.E+08	31	1.E+07	Lipoprotein signal peptide (Sec/SPII)	0.524	-	Cytoplasmic Membrane
B2UP66	tr|B2UP66|B2UP66_AKKM8 Dihydroorotase OS=Akkermansia muciniphila (strain ATCC BAA-835 / DSM 22959 / JCM 33894 / BCRC 81048 / CCUG 64013 / CIP 107961 / Muc) OX=349741 GN=pyrC PE=3 SV=1	Amuc_2027	5	5	16.80	36.40	11.33	2.E+08	14	1.E+07	TAT signal peptide (Tat/SPI)	0.811	-	Cytoplasm
B2URF3	tr|B2URF3|B2URF3_AKKM8 Uncharacterized protein OS=Akkermansia muciniphila (strain ATCC BAA-835 / DSM 22959 / JCM 33894 / BCRC 81048 / CCUG 64013 / CIP 107961 / Muc) OX=349741 GN=Amuc_1213 PE=4 SV=1	Amuc_1213	2	2	19.70	16.13	5.45	7.E+07	2	9.E+06	Signal peptide (Sec/SPI)	0.989	-	Unknown
B2UL75	sp|B2UL75|G1091_AKKM8 Glycosyl hydrolase family 109 protein 1 OS=Akkermansia muciniphila (strain ATCC BAA-835 / DSM 22959 / JCM 33894 / BCRC 81048 / CCUG 64013 / CIP 107961 / Muc) OX=349741 GN=Amuc_0017 PE=3 SV=1	Amuc_0017	4	4	11.40	53.80	43.96	2.E+08	14	9.E+06	TAT signal peptide (Tat/SPI)	0.999	-	Unknown
B2UNI9	tr|B2UNI9|B2UNI9_AKKM8 Phosphopantothenoylcysteine decarboxylase OS=Akkermansia muciniphila (strain ATCC BAA-835 / DSM 22959 / JCM 33894 / BCRC 81048 / CCUG 64013 / CIP 107961 / Muc) OX=349741 GN=Amuc_0463 PE=4 SV=1	Amuc_0463	2	2	14.00	19.18	7.73	6.E+07	13	9.E+06	Signal peptide (Sec/SPI)	0.530	-	Cytoplasm
B2ULS2	tr|B2ULS2|B2ULS2_AKKM8 Sulfatase OS=Akkermansia muciniphila (strain ATCC BAA-835 / DSM 22959 / JCM 33894 / BCRC 81048 / CCUG 64013 / CIP 107961 / Muc) OX=349741 GN=Amuc_0121 PE=3 SV=1	Amuc_0121	6	6	16.20	57.46	22.83	3.E+08	36	9.E+06	Lipoprotein signal peptide (Sec/SPII)	0.765	0.779	Unknown
B2UN08	tr|B2UN08|B2UN08_AKKM8 Peptidase S15 OS=Akkermansia muciniphila (strain ATCC BAA-835 / DSM 22959 / JCM 33894 / BCRC 81048 / CCUG 64013 / CIP 107961 / Muc) OX=349741 GN=Amuc_1801 PE=4 SV=1	Amuc_1801	5	5	20.10	39.75	23.41	2.E+08	18	9.E+06	Signal peptide (Sec/SPI)	0.810	0.609	Unknown

B2UKW1	tr|B2UKW1|B2UKW1_AKKM8 Uncharacterized protein OS=Akkermansia muciniphila (strain ATCC BAA-835 / DSM 22959 / JCM 33894 / BCRC 81048 / CCUG 64013 / CIP 107961 / Muc) OX=349741 GN=Amuc_1411 PE=4 SV=1	Amuc_1411	2	2	16.10	21.91	5.25	4.E+07	2	7.E+06	Signal peptide (Sec/SPI)	0.964	0.950	Unknown
B2URC9	sp|B2URC9|RL28_AKKM8 50S ribosomal protein L28 OS=Akkermansia muciniphila (strain ATCC BAA-835 / DSM 22959 / JCM 33894 / BCRC 81048 / CCUG 64013 / CIP 107961 / Muc) OX=349741 GN=rpmB PE=3 SV=1	Amuc_1189	2	2	23.00	9.85	4.41	2.E+07	3	6.E+06	-	-	0.549	Unknown
B2UNM5	tr|B2UNM5|B2UNM5_AKKM8 Periplasmic binding protein OS=Akkermansia muciniphila (strain ATCC BAA-835 / DSM 22959 / JCM 33894 / BCRC 81048 / CCUG 64013 / CIP 107961 / Muc) OX=349741 GN=Amuc_1928 PE=4 SV=1	Amuc_1928	3	3	14.00	32.90	6.88	1.E+08	22	5.E+06	Signal peptide (Sec/SPI)	0.996	-	Unknown
B2UPI5	tr|B2UPI5|B2UPI5_AKKM8 Exo-alpha-sialidase OS=Akkermansia muciniphila (strain ATCC BAA-835 / DSM 22959 / JCM 33894 / BCRC 81048 / CCUG 64013 / CIP 107961 / Muc) OX=349741 GN=Amuc_0625 PE=4 SV=1	Amuc_0625	2	2	5.70	45.59	3.33	1.E+08	6	5.E+06	Signal peptide (Sec/SPI)	0.917	0.945	Periplasmic space
B2UQU9	tr|B2UQU9|B2UQU9_AKKM8 Glycoside hydrolase family 31 OS=Akkermansia muciniphila (strain ATCC BAA-835 / DSM 22959 / JCM 33894 / BCRC 81048 / CCUG 64013 / CIP 107961 / Muc) OX=349741 GN=Amuc_1008 PE=3 SV=1	Amuc_1008	4	4	5.30	146.26	11.39	2.E+08	15	3.E+06	Signal peptide (Sec/SPI)	0.856	0.860	Unknown
B2UND5	tr|B2UND5|B2UND5_AKKM8 Putative substrate-binding protein of aliphatic sulfonate ABC transporter OS=Akkermansia muciniphila (strain ATCC BAA-835 / DSM 22959 / JCM 33894 / BCRC 81048 / CCUG 64013 / CIP 107961 / Muc) OX=349741 GN=Amuc_0409 PE=4 SV=1	Amuc_0409	2	2	8.30	38.40	3.16	5.E+07	13	3.E+06	Lipoprotein signal peptide (Sec/SPII)	0.997	-	Cytoplasm
B2UN50	tr|B2UN50|B2UN50_AKKM8 PDZ/DHR/GLGF domain protein OS=Akkermansia muciniphila (strain ATCC BAA-835 / DSM 22959 / JCM 33894 / BCRC 81048 / CCUG 64013 / CIP 107961 / Muc) OX=349741 GN=Amuc_1844 PE=4 SV=1	Amuc_1844	2	2	10.00	34.08	5.79	2.E+07	3	1.E+06	Lipoprotein signal peptide (Sec/SPII)	0.632	0.951	Unknown
B2UNY2	tr|B2UNY2|B2UNY2_AKKM8 Peptidoglycan glycosyltransferase OS=Akkermansia muciniphila (strain ATCC BAA-835 / DSM 22959 / JCM 33894 / BCRC 81048 / CCUG 64013 / CIP 107961 / Muc) OX=349741 GN=Amuc_0514 PE=4 SV=1	Amuc_0514	2	2	4.10	89.28	7.04	3.E+07	3	7.E+05	Signal peptide (Sec/SPI)	0.861	0.823	Cytoplasmic Membrane
B2UR84	tr|B2UR84|B2UR84_AKKM8 YD repeat protein OS=Akkermansia muciniphila (strain ATCC BAA-835 / DSM 22959 / JCM 33894 / BCRC 81048 / CCUG 64013 / CIP 107961 / Muc) OX=349741 GN=Amuc_1143 PE=4 SV=1	Amuc_1143	3	3	2.40	217.62	2.39	6.E+07	4	7.E+05	-	-	0.943	Unknown
B2URL5	tr|B2URL5|B2URL5_AKKM8 Uncharacterized protein OS=Akkermansia muciniphila (strain ATCC BAA-835 / DSM 22959 / JCM 33894 / BCRC 81048 / CCUG 64013 / CIP 107961 / Muc) OX=349741 GN=Amuc_1275 PE=4 SV=1	Amuc_1275	2	2	6.60	38.86	3.84	2.E+06	2	1.E+05	Signal peptide (Sec/SPI)	0.786	-	Unknown

Supplementary Table 4. List of putative extracellular proteins secreted from *A. muciniphila* cultivated under basal medium.

Majority protein IDs	Fasta headers	Gene locus	Peptides	Unique peptides	Sequence coverage [%]	Mol. weight [kDa]	Score	Intensity	MS/MS count	iBAQ	SignalP		Secretoem P (Probability)	PSORTb 3.0 (subcellular localization)
											Signal peptide	Probability		
B2UKV9	tr|B2UKV9|B2UKV9_AKKM8 PepSY_like domain-containing protein OS=Akkermansia muciniphila (strain ATCC BAA-835 / DSM 22959 / JCM 33894 / BCRC 81048 / CCUG 64013 / CIP 107961 / Muc) OX=349741 GN=Amuc_1409 PE=4 SV=1	Amuc_1409	8	8	52.00	16.52	245.16	4.E+10	538	5.E+09	Signal peptide (Sec/SPI)	0.998	-	Unknown
B2UMX5	tr|B2UMX5|B2UMX5_AKKM8 OsmC family protein OS=Akkermansia muciniphila (strain ATCC BAA-835 / DSM 22959 / JCM 33894 / BCRC 81048 / CCUG 64013 / CIP 107961 / Muc) OX=349741 GN=Amuc_0342 PE=4 SV=1	Amuc_0342	7	7	65.20	14.48	113.32	5.E+09	262	8.E+08	-	-	0.660	Unknown
B2UMM9	tr|B2UMM9|B2UMM9_AKKM8 Uncharacterized protein OS=Akkermansia muciniphila (strain ATCC BAA-835 / DSM 22959 / JCM 33894 / BCRC 81048 / CCUG 64013 / CIP 107961 / Muc) OX=349741 GN=Amuc_1767 PE=4 SV=1	Amuc_1767	11	11	47.60	32.22	317.01	4.E+09	236	2.E+08	Signal peptide (Sec/SPI)	0.982	-	Unknown
B2UP96	tr|B2UP96|B2UP96_AKKM8 Thioredoxin domain-containing protein OS=Akkermansia muciniphila (strain ATCC BAA-835 / DSM 22959 / JCM 33894 / BCRC 81048 / CCUG 64013 / CIP 107961 / Muc) OX=349741 GN=Amuc_2057 PE=4 SV=1	Amuc_2057	4	4	29.30	18.12	48.08	2.E+09	144	2.E+08	Signal peptide (Sec/SPI)	0.936	0.934	Unknown
B2ULU7	tr|B2ULU7|B2ULU7_AKKM8 Thioredoxin domain OS=Akkermansia muciniphila (strain ATCC BAA-835 / DSM 22959 / JCM 33894 / BCRC 81048 / CCUG 64013 / CIP 107961 / Muc) OX=349741 GN=Amuc_0147 PE=4 SV=1	Amuc_0147	9	9	29.60	34.97	86.17	4.E+09	274	2.E+08	Signal peptide (Sec/SPI)	0.981	0.914	Unknown
B2UQ02	tr|B2UQ02|B2UQ02_AKKM8 Uncharacterized protein OS=Akkermansia muciniphila (strain ATCC BAA-835 / DSM 22959 / JCM 33894 / BCRC 81048 / CCUG 64013 / CIP 107961 / Muc) OX=349741 GN=Amuc_0700 PE=4 SV=1	Amuc_0700	2	2	13.70	15.12	51.76	5.E+08	26	2.E+08	Lipoprotein signal peptide (Sec/SPII)	0.999	0.934	Unknown
B2UQP6	tr|B2UQP6|B2UQP6_AKKM8 Peptidyl-prolyl cis-trans isomerase OS=Akkermansia muciniphila (strain ATCC BAA-835 / DSM 22959 / JCM 33894 / BCRC 81048 / CCUG 64013 / CIP 107961 / Muc) OX=349741 GN=Amuc_0949 PE=3 SV=1	Amuc_0949	2	2	12.70	20.97	47.03	6.E+08	127	1.E+08	Signal peptide (Sec/SPI)	0.807	0.786	Unknown
B2UNZ3	tr|B2UNZ3|B2UNZ3_AKKM8 Cycloartenol synthase-like protein OS=Akkermansia muciniphila (strain ATCC BAA-835 / DSM 22959 / JCM 33894 / BCRC 81048 / CCUG 64013 / CIP 107961 / Muc) OX=349741 GN=Amuc_0525 PE=4 SV=1	Amuc_0525	8	8	30.40	43.36	42.09	1.E+09	69	5.E+07	Signal peptide (Sec/SPI)	0.967	0.936	Unknown
B2UKX4	tr|B2UKX4|B2UKX4_AKKM8 Kelch repeat-containing protein OS=Akkermansia muciniphila (strain ATCC BAA-835 / DSM 22959 / JCM 33894 / BCRC 81048 / CCUG 64013 / CIP 107961 / Muc) OX=349741 GN=Amuc_1425 PE=4 SV=1	Amuc_1425	2	2	9.20	35.13	29.39	7.E+08	56	5.E+07	-	-	0.910	Unknown
B2UPD6	tr|B2UPD6|B2UPD6_AKKM8 Peptidase M16 domain protein OS=Akkermansia muciniphila (strain ATCC BAA-835 / DSM 22959 / JCM 33894 / BCRC 81048 / CCUG 64013 / CIP 107961 / Muc) OX=349741 GN=Amuc_0576 PE=3 SV=1	Amuc_0576	22	22	16.90	160.48	199.61	4.E+09	264	5.E+07	-	-	0.794	Unknown
B2UQ36	tr|B2UQ36|B2UQ36_AKKM8 YD repeat protein OS=Akkermansia muciniphila (strain ATCC BAA-835 / DSM 22959 / JCM 33894 / BCRC 81048 / CCUG 64013 / CIP 107961 / Muc) OX=349741 GN=Amuc_0735 PE=4 SV=1	Amuc_0735	23	23	19.20	212.82	161.17	4.E+09	172	4.E+07	-	-	0.932	Unknown

B2UQJ3	tr|B2UQJ3|B2UQJ3_AKKM8 PepSY_like domain-containing protein OS=Akkermansia muciniphila (strain ATCC BAA-835 / DSM 22959 / JCM 33894 / BCRC 81048 / CCUG 64013 / CIP 107961 / Muc) OX=349741 GN=Amuc_0895 PE=4 SV=1	Amuc_0895	3	3	24.30	16.87	8.63	3.E+08	24	4.E+07	Signal peptide (Sec/SPI)	0.982	0.591	Unknown
B2UQI1	tr|B2UQI1|B2UQI1_AKKM8 Uncharacterized protein OS=Akkermansia muciniphila (strain ATCC BAA-835 / DSM 22959 / JCM 33894 / BCRC 81048 / CCUG 64013 / CIP 107961 / Muc) OX=349741 GN=Amuc_0883 PE=4 SV=1	Amuc_0883	2	2	21.00	6.74	29.80	8.E+07	4	4.E+07	-	-	0.683	Unknown
B2URJ1	tr|B2URJ1|B2URJ1_AKKM8 Carbamoyl-phosphate synthase large chain OS=Akkermansia muciniphila (strain ATCC BAA-835 / DSM 22959 / JCM 33894 / BCRC 81048 / CCUG 64013 / CIP 107961 / Muc) OX=349741 GN=carB PE=3 SV=1	Amuc_1251	16	16	21.00	117.47	120.87	2.E+09	230	3.E+07	Lipoprotein signal peptide (Sec/SPII)	0.713	-	Unknown
B2UN11	tr|B2UN11|B2UN11_AKKM8 Flavodoxin-like domain-containing protein OS=Akkermansia muciniphila (strain ATCC BAA-835 / DSM 22959 / JCM 33894 / BCRC 81048 / CCUG 64013 / CIP 107961 / Muc) OX=349741 GN=Amuc_1804 PE=4 SV=1	Amuc_1804	2	2	14.00	19.22	8.61	3.E+08	16	3.E+07	-	-	0.813	Unknown
B2UL94	tr|B2UL94|B2UL94_AKKM8 YD repeat protein OS=Akkermansia muciniphila (strain ATCC BAA-835 / DSM 22959 / JCM 33894 / BCRC 81048 / CCUG 64013 / CIP 107961 / Muc) OX=349741 GN=Amuc_0036 PE=4 SV=1	Amuc_0036	26	26	19.70	214.69	147.05	2.E+09	211	2.E+07	-	-	0.937	Unknown
B2UPB5	tr|B2UPB5|B2UPB5_AKKM8 Polysaccharide export protein OS=Akkermansia muciniphila (strain ATCC BAA-835 / DSM 22959 / JCM 33894 / BCRC 81048 / CCUG 64013 / CIP 107961 / Muc) OX=349741 GN=Amuc_2077 PE=4 SV=1	Amuc_2077	4	4	20.40	28.03	35.78	3.E+08	35	2.E+07	Lipoprotein signal peptide (Sec/SPII)	0.960	0.726	Unknown
B2ULS7	tr|B2ULS7|B2ULS7_AKKM8 Transposase IS3/IS911 family protein OS=Akkermansia muciniphila (strain ATCC BAA-835 / DSM 22959 / JCM 33894 / BCRC 81048 / CCUG 64013 / CIP 107961 / Muc) OX=349741 GN=Amuc_0127 PE=4 SV=1	Amuc_0127	3	3	31.70	13.95	16.53	1.E+08	9	2.E+07	-	-	0.739	Unknown
B2URF3	tr|B2URF3|B2URF3_AKKM8 Uncharacterized protein OS=Akkermansia muciniphila (strain ATCC BAA-835 / DSM 22959 / JCM 33894 / BCRC 81048 / CCUG 64013 / CIP 107961 / Muc) OX=349741 GN=Amuc_1213 PE=4 SV=1	Amuc_1213	2	2	19.70	16.13	5.45	7.E+07	2	9.E+06	Signal peptide (Sec/SPI)	0.989	-	Unknown
B2UL75	sp|B2UL75|G1091_AKKM8 Glycosyl hydrolase family 109 protein 1 OS=Akkermansia muciniphila (strain ATCC BAA-835 / DSM 22959 / JCM 33894 / BCRC 81048 / CCUG 64013 / CIP 107961 / Muc) OX=349741 GN=Amuc_0017 PE=3 SV=1	Amuc_0017	4	4	11.40	53.80	43.96	2.E+08	14	9.E+06	TAT signal peptide (Tat/SPI)	0.999	-	Unknown
B2ULS2	tr|B2ULS2|B2ULS2_AKKM8 Sulfatase OS=Akkermansia muciniphila (strain ATCC BAA-835 / DSM 22959 / JCM 33894 / BCRC 81048 / CCUG 64013 / CIP 107961 / Muc) OX=349741 GN=Amuc_0121 PE=3 SV=1	Amuc_0121	6	6	16.20	57.46	22.83	3.E+08	36	9.E+06	Lipoprotein signal peptide (Sec/SPII)	0.765	0.779	Unknown
B2UN08	tr|B2UN08|B2UN08_AKKM8 Peptidase S15 OS=Akkermansia muciniphila (strain ATCC BAA-835 / DSM 22959 / JCM 33894 / BCRC 81048 / CCUG 64013 / CIP 107961 / Muc) OX=349741 GN=Amuc_1801 PE=4 SV=1	Amuc_1801	5	5	20.10	39.75	23.41	2.E+08	18	9.E+06	Signal peptide (Sec/SPI)	0.810	0.609	Unknown
B2UKW1	tr|B2UKW1|B2UKW1_AKKM8 Uncharacterized protein OS=Akkermansia muciniphila (strain ATCC BAA-835 / DSM 22959 / JCM 33894 / BCRC 81048 / CCUG 64013 / CIP 107961 / Muc) OX=349741 GN=Amuc_1411 PE=4 SV=1	Amuc_1411	2	2	16.10	21.91	5.25	4.E+07	2	7.E+06	Signal peptide (Sec/SPI)	0.964	0.950	Unknown

B2URC9	sp|B2URC9|RL28_AKKM8 50S ribosomal protein L28 OS=Akkermansia muciniphila (strain ATCC BAA-835 / DSM 22959 / JCM 33894 / BCRC 81048 / CCUG 64013 / CIP 107961 / Muc) OX=349741 GN=rpmB PE=3 SV=1	Amuc_1189	2	2	23.00	9.85	4.41	2.E+07	3	6.E+06	-	-	0.549	Unknown
B2UNM5	tr|B2UNM5|B2UNM5_AKKM8 Periplasmic binding protein OS=Akkermansia muciniphila (strain ATCC BAA-835 / DSM 22959 / JCM 33894 / BCRC 81048 / CCUG 64013 / CIP 107961 / Muc) OX=349741 GN=Amuc_1928 PE=4 SV=1	Amuc_1928	3	3	14.00	32.90	6.88	1.E+08	22	5.E+06	Signal peptide (Sec/SPI)	0.996	-	Unknown
B2UQU9	tr|B2UQU9|B2UQU9_AKKM8 Glycoside hydrolase family 31 OS=Akkermansia muciniphila (strain ATCC BAA-835 / DSM 22959 / JCM 33894 / BCRC 81048 / CCUG 64013 / CIP 107961 / Muc) OX=349741 GN=Amuc_1008 PE=3 SV=1	Amuc_1008	4	4	5.30	146.26	11.39	2.E+08	15	3.E+06	Signal peptide (Sec/SPI)	0.856	0.860	Unknown
B2UN50	tr|B2UN50|B2UN50_AKKM8 PDZ/DHR/GLGF domain protein OS=Akkermansia muciniphila (strain ATCC BAA-835 / DSM 22959 / JCM 33894 / BCRC 81048 / CCUG 64013 / CIP 107961 / Muc) OX=349741 GN=Amuc_1844 PE=4 SV=1	Amuc_1844	2	2	10.00	34.08	5.79	2.E+07	3	1.E+06	Lipoprotein signal peptide (Sec/SPII)	0.632	0.951	Unknown
B2UR84	tr|B2UR84|B2UR84_AKKM8 YD repeat protein OS=Akkermansia muciniphila (strain ATCC BAA-835 / DSM 22959 / JCM 33894 / BCRC 81048 / CCUG 64013 / CIP 107961 / Muc) OX=349741 GN=Amuc_1143 PE=4 SV=1	Amuc_1143	3	3	2.40	217.62	2.39	6.E+07	4	7.E+05	-	-	0.943	Unknown
B2URL5	tr|B2URL5|B2URL5_AKKM8 Uncharacterized protein OS=Akkermansia muciniphila (strain ATCC BAA-835 / DSM 22959 / JCM 33894 / BCRC 81048 / CCUG 64013 / CIP 107961 / Muc) OX=349741 GN=Amuc_1275 PE=4 SV=1	Amuc_1275	2	2	6.60	38.86	3.84	2.E+06	2	1.E+05	Signal peptide (Sec/SPI)	0.786	-	Unknown

Supplementary Table 5. List of proteins identified in secretome of *A. muciniphila* cultivated under BHI medium.

Majority protein IDs	Fasta headers	Gene locus	Peptides	Unique peptides	Sequence coverage [%]	Mol. weight [kDa]	Sequence lengths	Score	Intensity	MS/MS count	iBAQ
B2UKV9	tr|B2UKV9|B2UKV9_AKKM8 PepSY_like domain-containing protein OS=Akkermansia muciniphila (strain ATCC BAA-835 / DSM 22959 / JCM 33894 / BCRC 81048 / CCUG 64013 / CIP 107961 / Muc) OX=349741 GN=Amuc_1409 PE=4 SV=1	Amuc_1409	5	5	32.7	16.523	150	122.63	3.E+08	70	5.E+07
B2UP96	tr|B2UP96|B2UP96_AKKM8 Thioredoxin domain-containing protein OS=Akkermansia muciniphila (strain ATCC BAA-835 / DSM 22959 / JCM 33894 / BCRC 81048 / CCUG 64013 / CIP 107961 / Muc) OX=349741 GN=Amuc_2057 PE=4 SV=1	Amuc_2057	3	3	22.8	18.123	167	53.52	2.E+08	62	3.E+07
B2UKW7	tr|B2UKW7|B2UKW7_AKKM8 Glyceraldehyde-3-phosphate dehydrogenase OS=Akkermansia muciniphila (strain ATCC BAA-835 / DSM 22959 / JCM 33894 / BCRC 81048 / CCUG 64013 / CIP 107961 / Muc) OX=349741 GN=Amuc_1417 PE=3 SV=1	Amuc_1417	6	6	30.5	36.743	341	208.11	4.E+08	56	2.E+07
B2UQS2	sp|B2UQS2|ACP_AKKM8 Acyl carrier protein OS=Akkermansia muciniphila (strain ATCC BAA-835 / DSM 22959 / JCM 33894 / BCRC 81048 / CCUG 64013 / CIP 107961 / Muc) OX=349741 GN=acpP PE=3 SV=1	Amuc_0975	2	2	32.5	8.8206	80	138.88	2.E+08	53	8.E+07
B2URH0	tr|B2URH0|B2URH0_AKKM8 Uncharacterized protein OS=Akkermansia muciniphila (strain ATCC BAA-835 / DSM 22959 / JCM 33894 / BCRC 81048 / CCUG 64013 / CIP 107961 / Muc) OX=349741 GN=Amuc_1230 PE=4 SV=1	Amuc_1883	1	1	3.3	30.038	271	4.4739	3.E+08	40	3.E+07
B2UPZ3	tr|B2UPZ3|B2UPZ3_AKKM8 Thioredoxin OS=Akkermansia muciniphila (strain ATCC BAA-835 / DSM 22959 / JCM 33894 / BCRC 81048 / CCUG 64013 / CIP 107961 / Muc) OX=349741 GN=Amuc_0691 PE=3 SV=1	Amuc_0691	3	3	38.1	11.49	105	225.24	7.E+08	22	1.E+08
B2UMF9	sp|B2UMF9|PLSX_AKKM8 Phosphate acyltransferase OS=Akkermansia muciniphila (strain ATCC BAA-835 / DSM 22959 / JCM 33894 / BCRC 81048 / CCUG 64013 / CIP 107961 / Muc) OX=349741 GN=plsX PE=3 SV=1	Amuc_0265	1	1	4.3	37.054	349	20.287	3.E+08	18	2.E+07
B2URF3	tr|B2URF3|B2URF3_AKKM8 Uncharacterized protein OS=Akkermansia muciniphila (strain ATCC BAA-835 / DSM 22959 / JCM 33894 / BCRC 81048 / CCUG 64013 / CIP 107961 / Muc) OX=349741 GN=Amuc_1213 PE=4 SV=1	Amuc_1213	1	1	5.4	16.131	147	38.257	6.E+07	18	9.E+06
B2UR84	tr|B2UR84|B2UR84_AKKM8 YD repeat protein OS=Akkermansia muciniphila (strain ATCC BAA-835 / DSM 22959 / JCM 33894 / BCRC 81048 / CCUG 64013 / CIP 107961 / Muc) OX=349741 GN=Amuc_1143 PE=4 SV=1	Amuc_1143	2	2	1	217.62	1991	8.7575	4.E+07	17	5.E+05
B2ULB8	tr|B2ULB8|B2ULB8_AKKM8 Uncharacterized protein OS=Akkermansia muciniphila (strain ATCC BAA-835 / DSM 22959 / JCM 33894 / BCRC 81048 / CCUG 64013 / CIP 107961 / Muc) OX=349741 GN=Amuc_0062 PE=4 SV=1	Amuc_0062	1	1	3.4	26.955	235	4.9961	8.E+07	15	6.E+06
B2UMU2	tr|B2UMU2|B2UMU2_AKKM8 Elongation factor G OS=Akkermansia muciniphila (strain ATCC BAA-835 / DSM 22959 / JCM 33894 / BCRC 81048 / CCUG 64013 / CIP 107961 / Muc) OX=349741 GN=fusA PE=3 SV=1	Amuc_0306	1	1	3.8	78.782	715	85.686	7.E+07	8	2.E+06
B2UN03	tr|B2UN03|B2UN03_AKKM8 Uncharacterized protein OS=Akkermansia muciniphila (strain ATCC BAA-835 / DSM 22959 / JCM 33894 / BCRC 81048 / CCUG 64013 / CIP 107961 / Muc) OX=349741 GN=Amuc_0370 PE=4 SV=1	Amuc_0370	1	1	5.1	20.257	176	3.5135	3.E+07	7	3.E+06
B2UKP3	tr|B2UKP3|B2UKP3_AKKM8 Uncharacterized protein OS=Akkermansia muciniphila (strain ATCC BAA-835 / DSM 22959 / JCM 33894 / BCRC 81048 / CCUG 64013 / CIP 107961 / Muc) OX=349741 GN=Amuc_1342 PE=4 SV=1	Amuc_1342	1	1	5.4	34.981	312	9.6821	4.E+07	5	3.E+06
B2UPI3	tr|B2UPI3|B2UPI3_AKKM8 Glycosyl hydrolase BNR repeat-containing protein OS=Akkermansia muciniphila (strain ATCC BAA-835 / DSM 22959 / JCM 33894 / BCRC 81048 / CCUG 64013 / CIP 107961 / Muc) OX=349741 GN=Amuc_0623 PE=4 SV=1	Amuc_0623	1	1	2.1	48.372	436	3.2438	3.E+07	5	2.E+06
B2UQY2	tr|B2UQY2|B2UQY2_AKKM8 DNA-directed RNA polymerase subunit beta OS=Akkermansia muciniphila (strain ATCC BAA-835 / DSM 22959 / JCM 33894 / BCRC 81048 / CCUG 64013 / CIP 107961 / Muc) OX=349741 GN=rpoB PE=3 SV=1	Amuc_1041	1	1	1.8	146.23	1312	323.31	6.E+07	5	9.E+05
B2ULW5	tr|B2ULW5|B2ULW5_AKKM8 Twitching motility protein OS=Akkermansia muciniphila (strain ATCC BAA-835 / DSM 22959 / JCM 33894 / BCRC 81048 / CCUG 64013 / CIP 107961 / Muc) OX=349741 GN=Amuc_0165 PE=3 SV=1	Amuc_0165	1	1	8.1	40.417	360	163.34	4.E+07	3	2.E+06
B2UM02	tr|B2UM02|B2UM02_AKKM8 RNA polymerase sigma factor SigA OS=Akkermansia muciniphila (strain ATCC BAA-835 / DSM 22959 / JCM 33894 / BCRC 81048 / CCUG 64013 / CIP 107961 / Muc) OX=349741 GN=sigA PE=3 SV=1	Amuc_1626	2	2	2.6	78.262	688	3.328	3.E+07	3	1.E+06
B2UN86	tr|B2UN86|B2UN86_AKKM8 Beta-lactamase domain protein OS=Akkermansia muciniphila (strain ATCC BAA-835 / DSM 22959 / JCM 33894 / BCRC 81048 / CCUG 64013 / CIP 107961 / Muc) OX=349741 GN=Amuc_1883 PE=4 SV=1	Amuc_1883	1	1	4.2	50.21	456	3.422	7.E+08	2	4.E+07

B2UN30	tr|B2UN30|B2UN30_AKKM8 Uncharacterized protein OS=Akkermansia muciniphila (strain ATCC BAA-835 / DSM 22959 / JCM 33894 / BCRC 81048 / CCUG 64013 / CIP 107961 / Muc) OX=349741 GN=Amuc_1823 PE=4 SV=1;tr|B2UQW1|B2UQW1_AKKM8 Uncharacterized protein OS=Akkerman	Amuc_1823	1	1	3.7	61.69	545;546	40.295	9.E+06	2	3.E+05
B2UL03	tr|B2UL03|B2UL03_AKKM8 Uncharacterized protein OS=Akkermansia muciniphila (strain ATCC BAA-835 / DSM 22959 / JCM 33894 / BCRC 81048 / CCUG 64013 / CIP 107961 / Muc) OX=349741 GN=Amuc_1454 PE=4 SV=1	Amuc_1454	1	1	21.5	20.058	195	223.29	4.E+07	1	4.E+06
B2UMN9	tr|B2UMN9|B2UMN9_AKKM8 von Willebrand factor type A OS=Akkermansia muciniphila (strain ATCC BAA-835 / DSM 22959 / JCM 33894 / BCRC 81048 / CCUG 64013 / CIP 107961 / Muc) OX=349741 GN=Amuc_1777 PE=4 SV=1	Amuc_1777	1	1	2.1	85.513	754	3.3214	3.E+07	1	1.E+06
B2ULW6	tr|B2ULW6|B2ULW6_AKKM8 Twitching motility protein OS=Akkermansia muciniphila (strain ATCC BAA-835 / DSM 22959 / JCM 33894 / BCRC 81048 / CCUG 64013 / CIP 107961 / Muc) OX=349741 GN=Amuc_0166 PE=3 SV=1	Amuc_0166	1	1	8	39.925	364	46.086	1.E+07	1	7.E+05

Supplementary Table 6. List of putative extracellular proteins secreted from *A. muciniphila* cultivated under BHI medium.

Majority protein IDs	Fasta headers	Gene locus	Peptides	Unique peptides	Sequence coverage [%]	Mol. weight [kDa]	Sequence lengths	Score	Intensity	MS/MS count	iBAQ	SignalP		Secretoem P (Probability)	PSORTb 3.0 (subcellular localization)
												Signal peptide	Probability		
B2UKV9	tr|B2UKV9|B2UKV9_AKKM8 PepSY_like domain-containing protein OS=Akkermansia muciniphila (strain ATCC BAA-835 / DSM 22959 / JCM 33894 / BCRC 81048 / CCUG 64013 / CIP 107961 / Muc) OX=349741 GN=Amuc_1409 PE=4 SV=1	Amuc_1409	5	5	32.7	16.523	150	122.63	3.E+08	70	5.E+07	Signal peptide (Sec/SPI)	0.998	-	Unknown
B2UP96	tr|B2UP96|B2UP96_AKKM8 Thioredoxin domain-containing protein OS=Akkermansia muciniphila (strain ATCC BAA-835 / DSM 22959 / JCM 33894 / BCRC 81048 / CCUG 64013 / CIP 107961 / Muc) OX=349741 GN=Amuc_2057 PE=4 SV=1	Amuc_2057	3	3	22.8	18.123	167	53.52	2.E+08	62	3.E+07	Signal peptide (Sec/SPI)	0.936	0.934	Unknown
B2UR84	tr|B2UR84|B2UR84_AKKM8 YD repeat protein OS=Akkermansia muciniphila (strain ATCC BAA-835 / DSM 22959 / JCM 33894 / BCRC 81048 / CCUG 64013 / CIP 107961 / Muc) OX=349741 GN=Amuc_1143 PE=4 SV=1	Amuc_1143	2	2	1	217.62	1991	8.7575	4.E+07	17	5.E+05	-	-	0.943	Unknown

Supplementary Table 7. Sequences of qRT-PCR primers used in this study.

Gene	Primer seqence (5'-3')	
Mist1	Forward	AGAGCAATGAGCGAGAGAGG
	Reverse	GTGAGGGTCTCGATCTTGGA
Atoh1	Forward	GCCAGTTAGGAAGGCAACAG
	Reverse	ACAACGATCACCACAGACCA
Dll1	Forward	GTTGTCTCCATGGCACCTG
	Reverse	TGCACGGCTTATGGTGAGTA
Tph1	Forward	GTCCTGTGGCTGGTTACCTC
	Reverse	GCTCTGGAGTGTAGAGGGGA
Chga	Forward	CCCACTGCAGCATCCAGTT
	Reverse	CCGACTGACCATCATCTTTCTG
Gpr119	Forward	CCGTGGCTGATACCTTGATT
	Reverse	AGAGGCAGCTGCAGAAGAAG
Gpr120	Forward	AGAGGCTTACGCTGAGCTTG
	Reverse	GAAGGAAACCATGAGCAGGA
Prox1	Forward	TGAATCCCCAAGGTTCAGAG
	Reverse	AAAGGCATCATGGCATCTTC
Lyz1	Forward	ATGGCTACCGTGGTGTCAAG
	Reverse	ATCCCATAGTCGGTGCTTCG
Defa24	Forward	TGTAGAGCAAGAGGCTGCAA
	Reverse	CAGCATCAGTGGCCTCAGTA
Cd24a	Forward	CTTCTGGCACTGCTCCTACC
	Reverse	TACTTGGATTTGGGGAAGCA
Sox9	Forward	CGACTACGCTGACCATCAGA
	Reverse	AGACTGGTTGTTCCCAGTGC
Spdef	Forward	TTGGATGAGCACTCGCTAGA
	Reverse	AAAAGCCACTTCTGCACGTT
Tff3	Forward	GATTACGTTGGCCTGTGTCC
	Reverse	CAGGGCACATTTGGGATACT
Agr2	Forward	CAAATCTGGAGCCAAAAAGG
	Reverse	CCATCAAGGGTCTGTTGCTT
Manf	Forward	CCACCATATCCCTGTGGAAA
	Reverse	CGTCCAGGATCTTCTTCAGC
Muc2	Forward	CTGACCAAGAGCGAACACAA
	Reverse	CATGACTGGAAGCAACTGGA
Muc3	Forward	GCTGGCTTTCATCCTCCACT
	Reverse	GCTGTCGTCTTGGGTGCTAT
Muc4	Forward	GAGGGCTACTGTCACAATGGAGGC
	Reverse	AGGGTTCCGAAGAGGATCCCGTAG

Muc13	Forward	CTGCAACCCTAACCCCTGTA
	Reverse	CGTTCCTTTCACACATGACG
Fabp1	Forward	ATTCATGAAGGCAATAGGTCTG
	Reverse	TCATGCACGATTTCTGACAC
Fabp2	Forward	GTGGAAAGTAGACCGGAACGA
	Reverse	CCATCCTGTGTGATTGTCAGTT
Apoc3	Forward	CGTAGGTGCCATGCAGCCCC
	Reverse	CAGCTCGGGCAGATGCCAGG
Alpi	Forward	ATCATCTTCCTGGGAGACGG
	Reverse	CGCCGATGGTCTTGTAGTTG
Ki67	Forward	ATCCAGATGATGGAGCCAAG
	Reverse	ATTTCTGCAGCTGGTTTGCT
Pcna	Forward	CAAAGACCTCATCAATGAGG
	Reverse	GAGGTTCACGCCCATGGCTA
Ccnd1	Forward	CCAGCTCCTGTGCTGCGAAG
	Reverse	CATGGATGGCACAATCTCCT
Lgr5	Forward	CAGGTCAATACCGGAGCGAG
	Reverse	GCGAGGCACCATTCAAAGTC
Olfm4	Forward	GCCAGATCTTGGCTCTGAAG
	Reverse	GCCAGTTGAGCTGAATCACA
Msi1	Forward	GAGGACTCAGTTGGCAGACC
	Reverse	CGCCTGGTCCATGAAAGTGA
Bmi1	Forward	TGCTGGAGAGCTGGAAAGTG
	Reverse	GTGAGGGAACTGTGGGTGAG
Hopx	Forward	ACCAGGTGGAGATCCTGGAGTA
	Reverse	CCAGGCGCTGCTTAAACCAT
Axin2	Forward	CTCCCCACCTTGAATGAAGA
	Reverse	ACT GGGTCGCTTCTCTTGAA
Myc	Forward	GCTGTTTGAAGGCTGGATTTC
	Reverse	GATGAAATAGGGCTGTACGGAG
Ctnnb1	Forward	CCCAGTCCTTCACGCAAGAG
	Reverse	CATCTAGCGTCTCAGGGAACA
Prom1	Forward	GAAAAGTTGCTCTGCGAACC
	Reverse	TCTCAAGCTGAAAAGCAGCA
Tert	Forward	ACTCAGCAACCTCCAGCCTA
	Reverse	CATATTGGCACTCTGCATGG
Cd44	Forward	GTGGGCAGAAGAAAAAGCTG
	Reverse	TGATGGTTCCTTGTTCACCA
Ephb3	Forward	CGTGAAAGTGGACACCATTG
	Reverse	CCAAGTAGAAGCCAGCCTTG

Rnf43	Forward	ATGTAACCTCGTGGGTCTGC
	Reverse	GCCAACTTTCTGCTCCACTC
Znrf3	Forward	AGAAGCCATCGACCAGCTAA
	Reverse	AAGTACTCGGTGGGTTGTCG
18s rRNA	Forward	GACACGGACAGGATTGACAGATTGATAG
	Reverse	GTTAGCATGCCAGAGTCTCGTTCGTT
MKI67	Forward	TGACCCTGATGAGAAAGCTCAA
	Reverse	CCCTGAGCAACACTGTCTTTT
LGR5	Forward	TGCTCTTCACCAACTGCATC
	Reverse	CTCAGGCTCACCAGATCCTC
LRIG1	Forward	GACCCTTTCTGACCGACAA
	Reverse	CGCTTTCCACGGCTCTTT
CD166	Forward	TCAAGGTGTTCAAGCAACCA
	Reverse	CTGAAATGCAGTCACCCAAC
ASCL2	Forward	CGTGAAGCTGGTGAACTTGG
	Reverse	GGATGTACTCCACGGCTGAG
OLFM4	Forward	ACCTTTCCCGTGGACAGAGT
	Reverse	TGGACATATTCCCTCACTTTGGA
AXIN2	Forward	CTCCCCACCTTGAATGAAGA
	Reverse	GTTTCCGTGGACCTCACACT
CTNNB	Forward	TCTGAGGACAAGCCACAAGATTACA
	Reverse	TGGGCACCAATATCAAGTCCAA
SOX9	Forward	GGAGAGCGAGGAGGACAAGTTC
	Reverse	TTGAAGATGGCGTTGGGGG
TERT	Forward	CTACTCCTCAGGCGACAAGG
	Reverse	TGGAACCCAGAAAGATGGTC
WNT3	Forward	CGCCTCGGAGATGGTAGTAG
	Reverse	AAAGTTGGGGGAGTTCTCGT
CCND1	Forward	TGTTTGCAAGCAGGACTTTG
	Reverse	TCATCCTGGCAATGTGAGAA
GAPDH	Forward	GAAGGTGAAGGTCGGAGTC
	Reverse	GAAGATGGTGATGGGATTTC

Supplementary Table 8. List of antibodies used in this study.

Name of Antibody	Catalog No.	Company	Dilution used
E-Cadherin	BD-610181	BD Biosciences	1:1000 WB
Frizzled 7	ab64636	Abcam	1:1000 WB
LRP5 (D80F2)	5731S	Cell Signaling	1:1000 WB
LRP6 (C47E12)	3395S	Cell Signaling	1:1000 WB
β-catenin	BD-610153	BD Biosciences	1:1000 WB
Active β-catenin	19807S	Cell Signaling	1:1000 WB
β-Actin	4967S	Cell Signaling	1:1000 WB
6X His tag	ab9108	Abcam	1:1000 WB
Strep-tag	34850	QIAGEN	1:3000 WB
Amuc_1409	in-house-made	AbClon	1:1000 WB
Ki67	ab15580	Abcam	1:100 IF, 1:200 IHC
Chromogranin A	ab199192	Abcam	1:200 IF
Lysozyme	ab108508	Abcam	1:200 IF
MUC2	GTX100664	GeneTex	1:100 IF
Ki67	AB9260	Millipore	1:100 IF
Ki67	556003	BD Biosciences	1:100 IF
Brdu	B2531	Sigma Aldrich	1:100 IF
E-Cadherin	610182	BD Biosciences	1:200 IF
E-Cadherin	AF648	R&D systems	1:200 IF
ASCL2	MAB4418	Millipore	1:100 IF
GFP	ab13970	Abcam	1:100 IF
OLFM4	ab85046	Abcam	1:100 IF
OLFM4 (D6Y5A)	39141S	Cell Signaling	1:100 IHC
Western blot (WB), Immunofluorescence (IF), Immunohistochemistry (IHC)			

Supplementary Table 9. List of primer sequence used for E-cadherin cloning.

Primers	Primer seqence (5'-3')*
CDH1-F-SacII	TCCCCGCGGTCCAGGAGCCGGAG
CDH1-R-XhoI	CCGCTCGAGGGCTAGTCGTCCTCGCC
CDH1-EC-F-SacII	TCCCCGCGGTCGACTGGGTTATT
CDH1-EC-R-XhoI	CCGCTCGAGGGCTACAATCCTGCTTC
CDH1-IC-F-SacII	TCCCCGCGGTCAGAGCGGTGGTC
CDH1-IC-R-XhoI	CCGCTCGAGGGCTAGTCGTCCTCGCC

*Sites for restrictive endonucleases are underlined.
